# *NAT2* variants and toxicity related to anti-tuberculosis agents: a systematic review and meta-analysis

**DOI:** 10.5588/ijtld.18.0324

**Published:** 2019-03

**Authors:** M. Richardson, J. Kirkham, K. Dwan, D. J. Sloan, G. Davies, A. L. Jorgensen

**Affiliations:** * Department of Biostatistics, University of Liverpool, Liverpool; † Cochrane Editorial Unit, London; ‡ School of Medicine, University of St Andrews, St Andrews; § Department of Clinical Infection, Microbiology and Immunology, University of Liverpool, Liverpool, UK

**Keywords:** tuberculosis, pharmacogenetics, adverse events, evidence synthesis

## Abstract

**BACKGROUND:**

Tuberculosis (TB) patients receiving anti-tuberculosis treatment may experience serious adverse drug reactions (ADRs) such as hepatotoxicity. Variants of the *N*-acetyltransferase 2 (*NAT2*) gene may increase the risk of experiencing such toxicity events.

**OBJECTIVE:**

To provide a comprehensive evaluation of the evidence base for associations between *NAT2* variants and anti-tuberculosis drug-related toxicity.

**METHOD:**

This was a systematic review and meta-analysis. We searched for studies in Medline, PubMed, EMBASE, BIOSIS and Web of Science. We included data from 41 articles (39 distinct cohorts of patients). We pooled effect estimates for each genotype on each outcome using meta-analyses stratified by country.

**RESULTS:**

We assessed the quality of the included studies, which was variable, with many areas of concern. Slow/intermediate *NAT2* acetylators were statistically significantly more likely to experience hepatotoxicity than rapid acetylators (OR 1.59, 95%CI 1.26–2.01). Heterogeneity was not detected in the overall pooled analysis (*I*^2^ = 0%). *NAT2* acetylator status was significantly associated with the likelihood of experiencing anti-tuberculosis drug-related hepatotoxicity.

**CONCLUSION:**

We encountered several challenges in performing robust syntheses of data from pharmacogenetic studies, and we outline recommendations for the future reporting of pharmacogenetic studies to enable high-quality systematic reviews and meta-analyses to be performed.

## Abstract (FR)

**CADRE:**

Les patients tuberculeux recevant un traitement antituberculeux peuvent souffrir d'effets secondaires graves aux médicaments, comme la toxicité hépatique. Les variants génétique du gène *NAT2* pourraient accroître le risque de ces effets secondaires de la toxicité des médicaments.

**OBJECTIF:**

Fournir une évaluation complète des éléments en faveur de l'association entre les variants génétiques de *NAT2* et la toxicité des antituberculeux.

**MÉTHODE:**

Ceci est une revue systématique et méta-analyse. Nous avons recherché des études sur Medline, EMBASE, BIOSIS et le Web of Science. Nous avons inclus des données de 41 articles (39 cohortes distinctes de patients). Nous avons groupé l'estimation des effets pour chaque génotype et pour chaque résultat avec une méta-analyse stratifiée par pays. Nous avons réalisé une évaluation qualitative de la qualité des études incluses.

**RÉSULTATS:**

La qualité des études incluses a été variable, avec de nombreux sujets de préoccupation. Les acétyleurs lents/intermédiaires de *NAT2* ont été statistiquement significativement plus susceptibles de souffrir d'une hépatotoxicité que les acétyleurs rapides (OR 1,59 ; IC95% 1,26–2,01). Aucune hétérogénéité n'a été détectée dans l'ensemble de l'analyse regroupée (*I*^2^ = 0%). Le statut d'acétyleur de *NAT2* est significativement associé à la probabilité de toxicité hépatique liée aux médicaments antituberculeux.

**CONCLUSION:**

Nous avons rencontré de nombreux défis dans la réalisation d'une synthèse solide des données des études pharmacogénétiques et nous présentons des recommandations pour les futurs rapports des études pharmacogénétiques afin de permettre de réaliser des revues systématiques et des méta-analyses de bonne qualité.

## Abstract (ES)

**MARCO DE REFERENCIA:**

Los pacientes que reciben tratamiento antituberculoso pueden presentar reacciones adversas graves a los medicamentos, por ejemplo la hepatotoxicidad. Los polimorfismos genéticos de *NAT2* pueden aumentar el riesgo de aparición de este tipo de reacciones adversas.

**OBJETIVO:**

Aportar una evaluación exhaustiva de la base de datos probatorios sobre la asociación entre los polimorfismos genéticos de *NAT2* y la toxicidad atribuida a los medicamentos antituberculosos.

**MÉTODO:**

Esta es una revisión sistemática con metanálisis. Se buscaron artículos en las bases de datos Medline, EMBASE, BIOSIS y Web of Science. Se incluyeron datos de 41 artículos (39 cohortes de pacientes diferentes). Se combinaron las estimaciones del efecto para cada genotipo con cada resultado, mediante un metanálisis estratificado por países. Se realizó una evaluación cualitativa de los estudios incluidos.

**RESULTADOS:**

La calidad de los estudios incluidos fue variable, con muchos aspectos que son fuente de preocupación. La probabilidad de hepatotoxicidad en los acetiladores lentos e intermedios de *NAT2* fue significativamente mayor que en los acetiladores rápidos (OR 1,59; IC95% 1,26–2,01). No se detectó heterogeneidad en el análisis global combinado (*I*^2^ = 0%). El fenotipo acetilador de *NAT2* se asoció de manera significativa con la probabilidad de aparición de hepatotoxicidad debida a los medicamentos antituberculosos.

**CONCLUSIÓN:**

Se encontraron dificultades importantes al tratar de realizar una síntesis sólida de los datos de estudios farmacogenéticos, por lo cual se proponen recomendaciones sobre la comunicación de los resultados de futuros estudios farmacogenéticos, que faciliten la realización de revisiones sistemáticas y metanálisis de gran calidad.

TUBERCULOSIS (TB) is one of the most important challenges in global health. There were an estimated 1.3 million TB deaths in 2016 among human immunodeficiency virus (HIV) negative people and 374 000 deaths among HIV-positive people.^1^ The World Health Organization (WHO) recommends a combination of four first-line drugs for individuals with drug-susceptible TB: isoniazid (INH), rifampicin (RMP), ethambutol (EMB) and pyrazinamide (PZA).^1^

TB patients receiving a combination of these drugs may experience adverse drug reactions (ADRs), the most serious of which is anti-tuberculosis drug-induced hepatotoxicity (ATDH). Reported incidence rates of ATDH among patients treated with standard multidrug treatment vary from 2% to 28%, depending on the regimen given, definition of ATDH and patient characteristics such as age, race and sex.^2^ ATDH can be fatal, with reported mortality rates of 6–12% if drugs are not promptly stopped.^3^ ATDH and other anti-tuberculosis drug-related adverse effects also contribute to non-adherence, eventually leading to treatment failure, relapse and the emergence of drug resistance.^2^

The proposed genetic risk factors for ATDH include polymorphisms of the *N*-acetyltransferase 2 (*NAT2*) gene, which codes for the drug-metabolising enzyme, NAT2.^4^,^5^ *NAT2* polymorphisms may affect the activity of the NAT2 enzyme, altering the chemical modification of anti-tuberculosis drugs and their metabolites in the liver, leading to hepatic adverse reactions.^6^ Toxic metabolites may also cause other toxicity events, such as peripheral neuropathy and maculopapular eruption, although the majority of evidence on the pharmacogenetics of anti-tuberculosis drugs focuses on hepatotoxicity.

INH is the anti-tuberculosis drug for which the genetic contribution to ATDH has been most widely studied and is best understood. Specifically, it is thought that *NAT2* acetylator status may be associated with INH-related hepatotoxicity because NAT2 is one of the main enzymes involved in INH metabolism in the liver. There are three phenotypes of acetylator status. Individuals who are slow NAT2 acetylators have higher plasma drug concentrations. This may be beneficial for treatment efficacy, but may also cause an accumulation of toxic metabolites as part of the metabolic activation of acetylhydrazine to harmless diacetylhydrazine. INH suppresses the acetylation of acetylhydrazine to produce more toxic metabolites, which contributes to the increased risk of hepatitis.^7^ Fast acetylators have lower plasma drug concentrations, and so treatment may be less effective, but also less toxic. Intermediate acetylators fall between these two extremes.

RMP and PZA have also been reported to be hepatotoxic;^8^ however, the mechanisms for RMP-and PZA-induced hepatotoxicity are not known.^9^ The OATP1B1^*^15 haplotype has been reported to be a predictor of RMP-induced liver injury;^10^ no research into the genetic predictors of PZA-induced hepatotoxicity has been reported.^11^ No hepatotoxicity has been described for EMB.^8^

The objective of this systematic review and meta-analysis was to evaluate evidence on the effect of NAT2 on anti-tuberculosis drug-related toxicity in TB patients receiving anti-tuberculosis treatment. Meta-analyses investigating the effect of NAT2 on toxicity outcomes have been published,^6^,^12–15^ but the conclusions from these have been conflicting. Our review and meta-analysis updates and adds to the evidence base on associations between NAT2 and anti-tuberculosis drug-related toxicity.

## METHODS

This review was conducted in line with the methods outlined in our protocol (PROSPERO registration number: CRD42017068448).^16^ A search strategy and study selection process enabled identification of studies that investigated the association between any genetic variant and anti-tuberculosis drug-related toxicity. However, in this article, we focus only on the subset of studies that considered *NAT2* variants. Studies investigating associations between other genetic variants and anti-tuberculosis drug-related toxicity will be reported separately.

### Selection criteria

#### Types of studies

We included cohort studies, case-control studies and randomised controlled trials (RCTs). We did not include studies on case series because this type of study design would be inappropriate to investigate the effect of genetic variants on anti-tuberculosis drug-related toxicity. We did not require a minimum number of enrolled patients for a study to be included in our review.

#### Types of participants

We included studies that recruited TB patients who were either already established on anti-tuberculosis treatment or commencing treatment (at least one of INH, RMP, PZA or EMB), and who were genotyped to investigate the effect of genetic variants on anti-tuberculosis drug-related toxicity. We only included studies where >50% of included patients were TB patients receiving anti-tuberculosis treatment.

#### Types of outcomes

We included studies that measured any drug-related toxicity outcomes.

### Search strategy

An information specialist (EK) designed the search strategy (Appendix Tables A.1^*^ and A.2), and searched for relevant studies in Medline, PubMed, EMBASE, BIOSIS and Web of Science (date of search: 3 March 2016). We searched reference lists from relevant studies manually, and contacted experts to identify eligible studies. We included studies published in English only. We did not restrict by year of publication or publication status.

### Study selection

The search results were imported to Covidence.^17^ We removed duplicates, and one author (MR) scanned the study abstracts to remove irrelevant studies. A second author (AJ, JK or KD) independently screened a sample of 10% of studies.

We obtained the full text for each potentially relevant study. One reviewer (MR) assessed eligibility based on the selection criteria. A second author (AJ, JK or KD) independently assessed a sample of 10% of studies for eligibility. Disagreements between the two reviewers at the abstract and full-text screening stages were resolved through discussion, and by consulting a third author if necessary.

### Outcomes

The primary outcome of this review was hepatotoxicity by any definition used by the original investigators. The secondary outcomes were all other toxicity outcomes.

### Data collection

We designed and piloted a data extraction form. We collected data on study design, participant characteristics, and treatment regimen and outcomes. One author (MR) extracted data in accordance with the methods outlined in the Cochrane Handbook^18^ and The HuGENet HuGE Review Handbook.^19^ A second author (AJ, JK or KD) independently extracted all outcome data. Disagreements between the two reviewers were resolved through discussion, and by consulting a third author if necessary. We contacted study authors if outcome data necessary for inclusion in a meta-analysis were not published in the paper.

We contacted individuals who were listed as authors of multiple included articles to enquire whether there was overlap between articles in terms of the patient cohorts. We examined locations, dates of recruitment and other study characteristics to identify articles that reported outcomes for the same patient cohort. If an author confirmed that multiple articles reported outcomes for the same patient cohort, or if we suspected this based on reported study characteristics, we assigned a group identifier (GI) to these articles, and ensured that no data for the same patient cohort were included more than once in any meta-analysis.

### Quality assessment

One author applied criteria for the quality assessment of pharmacogenetic studies^20^ to each study. A second author (AJ) independently assessed the quality of a sample of 10% of studies. Disagreements between the two reviewers were resolved through discussion. We obtained the number of studies meeting each criterion and summarised this information in the text.

### Data synthesis

We performed meta-analyses for associations between *NAT2* and any anti-tuberculosis drug-related toxicity outcome that were investigated by at least two studies. The effects of both *NAT2* acetylator status (as predicted using genotyping methods) and individual *NAT2* single-nucleotide polymorphisms (SNPs) were investigated.

#### Primary analysis

The primary analysis compared risk of hepatotoxicity for slow/intermediate acetylators in comparison with rapid acetylators. Data were pooled from studies that reported data for each acetylator group separately together with data from studies that combined slow and intermediate acetylator groups.

Two sensitivity analyses were conducted. The first was pairwise comparisons of slow vs. rapid acetylator status, and intermediate vs. rapid acetylator status. Here, it was only possible to include data from studies that reported on each acetylator group separately. The second was comparison of slow vs. rapid/intermediate acetylator status. Here, data were pooled from studies that combined data for intermediate and rapid acetylator groups, and from studies that reported data for each acetylator group separately.

#### Secondary analysis

The secondary analysis compared the risk of hepatotoxicity between genotype groups for *NAT2* SNPs. For each SNP, two pairwise comparisons were undertaken: heterozygous genotype vs. homozygous wild-type (wt), and homozygous mutant-type vs. homozygous wt. For SNPs investigated by one study only, odds ratios (ORs) comparing genotype groups were calculated and summarised in a table, together with the pooled estimates from the meta-analyses. There were insufficient data to perform meta-analyses for an association between *NAT2* (acetylator status and individual SNPs) and other toxicity outcomes; ORs and 95% confidence intervals (CIs) for each pairwise comparison were calculated and reported in a table.

Meta-analyses were performed using Stata v 14 (metan package) (StataCorp, College Station, TX, USA);^21^ ORs with 95%CIs were the chosen measure of effect. We used the random-effects model because we anticipated heterogeneity between studies due to differences in study design, methodological quality, ethnicity of participants and outcome definitions. The random-effects model used the method of DerSimonian and Laird,^22^ with the estimate of heterogeneity being taken from the Mantel-Haenszel model.^23^ If zero events were observed in one of the genotype groups, a continuity correction of 0.5 was used. Data were excluded from the analysis if there were no patients in one of the genotype groups in a comparison.

The HuGENet HuGE Review Handbook recommends that meta-analyses of genetic association studies be stratified by ethnicity, and that meta-analyses should only be performed if effect estimates for different ethnic groups appear sufficiently similar.^19^ However, information on participants' ethnicity was sparsely reported in the studies included in our review. We therefore performed analyses stratified by the countries in which studies were conducted as a proxy for ethnicity.

### Investigation of heterogeneity

We assessed heterogeneity by visually examining forest plots, and by referring to the *I^2^* statistic. If substantial heterogeneity had been observed (>50%),^18^ we planned to undertake subgroup analyses according to study design, outcome definitions, treatment regimens and date of study publication.

### Selective reporting

We assessed the possibility of selective reporting as part of the quality assessment. Potential sources of selective reporting considered were genetic variants, outcomes and modes of inheritance.^20^

### Publication bias

We produced a funnel plot for the primary analysis to assess the risk of publication bias.

## RESULTS

### Included and excluded studies

**Figure 1:**
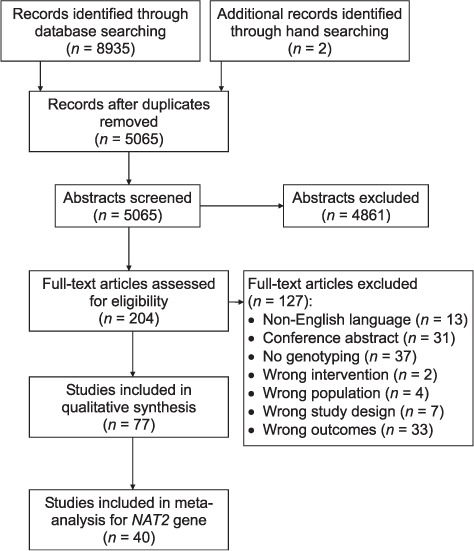
Flow chart of study according to the Preferred Reporting Items for Systematic Reviews and Meta-Analyses (PRISMA).^24^ NAT2 = N-acetyltransferase 2.

A Preferred Reporting Items for Systematic Reviews and Meta-Analyses (PRISMA) flow chart showing the selection of studies during the literature search is provided in Figure 1 (for more information, visit www.prisma-statement.org).^24^ The initial search identified 77 articles investigating the association between any genetic variant and anti-tuberculosis drug-related toxicity, from which 52 distinct cohorts of patients were identified (Figure 1).

Forty-six articles reported data for the association between *NAT2* variants and anti-tuberculosis drug-related toxicity; from these articles, 40 distinct patient cohorts were identified. In this review, we include data from 40 articles (39 distinct patient cohorts).^25–64^ We did not include data from the remaining six articles.^65–70^ Of those six articles, five reported data for patient cohorts for whom data were also reported in other articles (or we suspected that this was the case); for the sixth article,^70^ the numbers of patients in each genotype group were not reported, and we were unable to obtain this information from the authors. The characteristics of studies included in this review are provided in Appendix Table A.3.

### Quality assessment

#### Choosing which genes and SNPs to genotype

Twenty-seven articles reported the reasons for choosing all genes and SNPs investigated. For the 13 articles^28^,^31^,^33^,^37^,^48^,^51^,^53^,^57^,^59^,^60^,^62–64^ that did not report this information, no articles limited their reporting to only statistically significant associations. Therefore, there was no evidence to suggest that selective reporting of genes and SNPs had occurred.

#### Sample size

The median sample size was 170 (interquartile range 108.5–285). Only two articles^26^,^63^ provided details of the a priori power to detect pre-specified effect sizes.

#### Study design

Eleven articles described case-control studies, 27 articles described prospective cohorts, one article described a retrospective cohort and one article described an RCT. For one case-control study,^33^ the case and control groups were not clearly defined. No articles describing case-control studies reported that the two groups were genotyped in mixed batches.

#### Reliability of genotypes

Only three articles^26^,^32^,^46^ mentioned genotype quality control procedures, and only 12 articles^26^,^33^,^35^,^37^,^38^,^41^,^45^,^49–51^,^53^,^55^ compared the genotype frequencies of all investigated SNPs to those previously published for the same population. Of the articles describing case-control studies and retrospective cohorts, only two^45^,^46^ mentioned that genotyping personnel were blinded to outcome status.

#### Missing genotype data

For most articles (29/40), on comparison of the number of participants included in the analyses with the study sample size, it was apparent there were no missing genotype data. For the remaining 11 articles,^32^,^33^,^42–44^,^53^,^56^,^58^,^60^,^63^,^64^ only five articles^32^,^56^,^58^,^63^,^64^ summarised the extent of missing data for all the genes and SNPs analysed. None of these articles described checking whether missing data were randomly distributed.

#### Population stratification

One article mentioned undertaking tests for population stratification;^53^ no population stratification was identified. One article used a study design that ensured that the included patients were from a non-diverse ethnic group.^48^ All other studies were at potential risk from confounding due to population stratification.

#### Hardy-Weinberg equilibrium

Twenty-three articles^30^,^32^,^34–39^,^41–43^,^46–49^,^53^,^57^,^58^,^60–64^ reported testing for Hardy-Weinberg equilibrium (HWE) for all investigated SNPs, and a further three^25^,^51^,^56^ tested for HWE for a subset of SNPs. The remaining 14 articles reported no testing for HWE.

#### Mode of inheritance

Nineteen articles made a specific assumption regarding the underlying mode of inheritance.^25^,^29^,^31^,^34^,^35^,^40^,^43^,^44^,^48^,^50^,^53^,^55–57^,^59–61^,^63^,^64^ Of these, only two provided justification;^29^,^60^ for the remaining 17 articles, there was a risk of selective reporting under different modes of inheritance. Two articles^42^,^58^ applied models assuming different modes of inheritance to the genotype data, although only one of these articles^42^ adjusted these analyses for multiplicity of testing.

### Choice and definition of outcomes

There was large variation in the definition of hepatotoxicity (Appendix Table A.4). Of the 37 articles reporting hepatotoxicity data, one did not provide a definition,^62^ one provided a vague definition,^30^ and the remaining 35 articles provided 31 different definitions. Definitions of other toxicity outcomes were generally not sufficiently detailed (Appendix Table A.5).

Nine articles did not provide justification for the choice of outcomes, but outcomes were in line with the main study aim as conveyed in the Introduction section of the article.^27^,^32^,^38^,^49^,^50^,^52^,^56^,^57^,^63^ The remaining articles all provided justification for the choice of outcomes. There was therefore no evidence to suggest that selective reporting of outcomes had occurred.

#### Treatment adherence

Six articles^31^,^32^,^43^,^45^,^50^,^57^ mentioned assessing treatment adherence. One article^48^ reported that treatment was administered under DOTS; it was therefore not necessary to measure adherence. Of the six articles that reported assessing adherence, one did not report adjusting the analyses for adherence.^50^ It was not necessary to adjust for adherence in the analyses of two articles because patients were reported to have good treatment adherence.^31^,^32^

### Association between NAT2 variants and anti-tuberculosis drug-related toxicity

#### NAT2 acetylator status and hepatotoxicity

**Figure 2:**
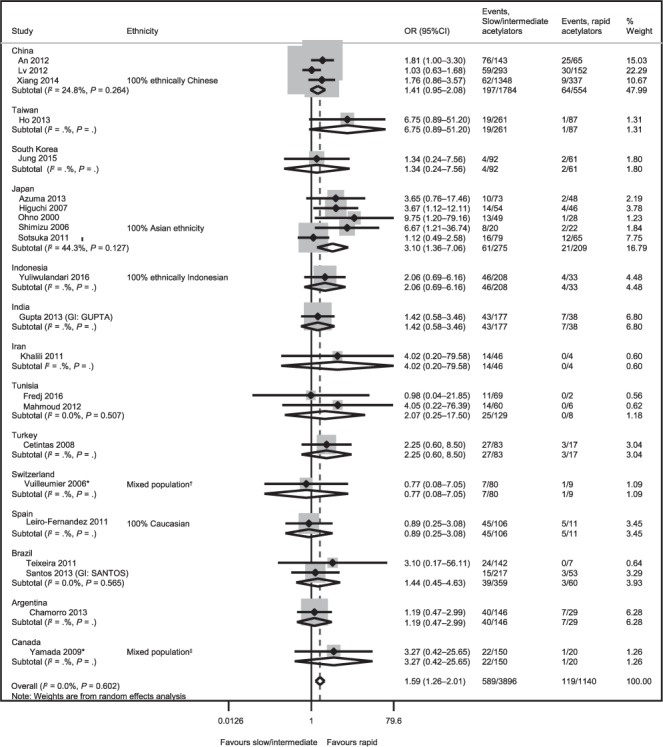
Slow/intermediate vs. rapid acetylator status for the outcome of hepatotoxicity. ^*^ Conducted in LTBI populations. ^†^Caucasian: 38 (43%), Hispanic: 8 (9%), African: 22 (25%), South American: 15 (17%), Asian: 5 (6%), Middle Eastern: 1 (1%). ^‡^Asian: 72 (42%), Caucasian: 49 (29%), South Asian: 22 (13%), Hispanic: 7 (4%), Middle Eastern: 8 (5%), First nations: 5 (3%), Other/mixed/unknown: 7 (4%). OR = odds ratio; CI = confidence interval; GI = group identifier; LTBI = latent tuberculous infection.

A forest plot displaying the results of the primary analysis is given in Figure 2. Slow/intermediate acetylators were significantly more likely to experience hepatotoxicity than rapid acetylators (OR 1.59, 95%CI 1.26–2.01). No heterogeneity was detected in this analysis (*I*^2^ = 0%).

**Figure A.1:**
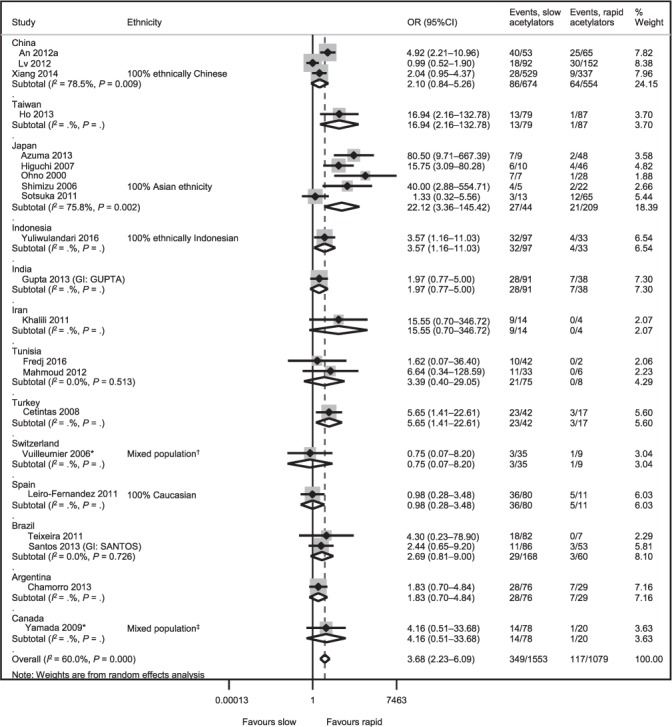
Sensitivity analysis 1: pairwise comparisons of slow vs. rapid acetylator status for the outcome of hepatotoxicity. ^*^ Conducted in LTBI populations. ^†^ Caucasian (n = 38, 43%), Hispanic (n = 8, 9%), African (n = 22, 25%), South American (n = 15, 17%), Asian (n = 5, 6%), Middle Eastern (n = 1, 1%). ^‡^ Asian (n = 72, 42%), Caucasian (n = 49, 29%), South Asian (n = 22, 13%), Hispanic (n =7, 4%), Middle Eastern (n =8, 5%), First nations (n =5, 3%), Other/mixed/unknown: (n =7, 4%). OR =odds ratio; CI = confidence interval; GI = group identifier; LTBI = latent tuberculous infection.

Results of the sensitivity analyses are provided in Appendix Figures A.1–A.3. Results from the pairwise comparisons suggested that slow acetylators were significantly more likely to experience hepatotoxicity than rapid acetylators (OR 3.68, 95%CI 2.23–6.09, *I*^2^ = 60.0%), but there were no significant differences between intermediate and rapid acetylators (OR 1.12, 95%CI 0.87–1.45, *I*^2^ = 0%). The sensitivity analysis that compared slow acetylators with rapid/intermediate acetylators suggested that slow acetylator status significantly increased the risk of hepatotoxicity (OR 3.12, 95%CI 2.45–3.97, *I*^2^ = 59.0%).

Moderate heterogeneity was observed in the sensitivity analyses of slow vs. rapid acetylator status, and slow vs. rapid/intermediate acetylator status. Such moderate heterogeneity may have been due to the variable distribution of genotypes in different geographic areas.

**Figure A.4:**
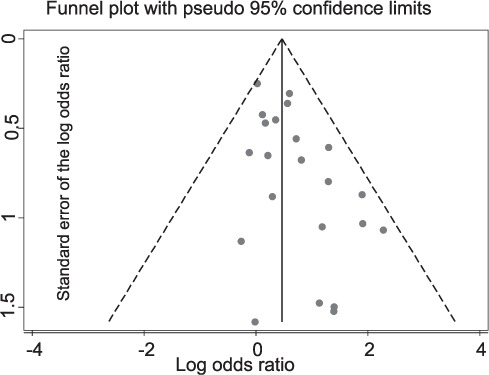
Funnel plot for the primary analysis.

The funnel plot for the primary analysis (Appendix Figure A.4) provided no evidence of publication bias.

#### NAT2 SNPs and hepatotoxicity

The included studies reported data for 12 *NAT2* SNPs. A summary of all data for the association between *NAT2* SNPs and hepatotoxicity is provided in Table 1. There were sufficient data to perform meta-analyses for six SNPs. Forest plots showing the results of these meta-analyses are provided in Figure 2. The four main findings from these meta-analyses are shown below.

1 For 590G-A and 857G-A, both heterozygous genotype and homozygous mutant-type significantly increased hepatotoxicity risk compared with homozygous wt (590G-A: GA vs. GG, OR 1.30, 95%CI 1.06–1.59, *I*^2^ =0%; AA vs. GG, OR 2.05, 95%CI 1.24–3.40, *I*^2^ =47.7%; 857G-A: GA vs. GG, OR 1.30, 95%CI 1.03–1.64, *I*^2^ = 0.9%; AA vs. GG, OR 1.99, 95%CI 1.02–3.91, *I*^2^ = 11.3%).
2 For 282C-T, homozygous mutant-type significantly increased hepatotoxicity risk compared with homozygous wt (OR 3.95, 95%CI 2.21–7.05, *I*^2^ = 5.5%); however, no significant difference was observed for heterozygous genotype compared with homozygous wt (OR 1.27, 95%CI 0.80–2.02, *I*^2^ = 0%).
3 For 481C-T, heterozygous genotype significantly increased hepatotoxicity risk compared with homozygous wt (OR 1.48, 95%CI 1.12–1.97, *I*^2^ = 0%); however, no significant difference was observed for homozygous mutant-type compared with homozygous wt (OR 1.91, 95%CI 0.93–3.92, *I*^2^ = 34.1%). The lack of statistical significance for the latter comparison may have been caused by the relatively small number of homozygous mutant-type patients (*n* = 162) among the patients contributing data to this analysis (*n* = 3604)
4 For 341T-C and 803A-G, no significant differences were observed for either pairwise comparison (341T-C: TC vs. TT, OR 1.15, 95%CI 0.72–1.82, *I*^2^=0%; CC vs. TT, OR 1.54, 95%CI 0.58–4.04, *I*^2^ = 0%; 803A-G: AG vs. AA, OR 1.14, 95%CI 0.67–1.96, *I*^2^ =0%; GG vs. AA, OR 1.90, 95%CI 0.66–5.52, *I*^2^ = 0%).

**Table 1:** Summary of all reported data for the association between NAT2 SNPs and hepatotoxicity

*NAT2* SNP	Comparison	Country (number of studies)	Ethnicity	OR (95%CI)	Cases *n*	Controls *n*	*I*^2^
190C-T	Het (CT) vs. hom wt (CC)	China (1 study)	NR	0.21 (0.01–4.38)	101	107	NA
	Hom MT (TT) vs. hom wt (CC)	China (1 study)	NR	Data excluded^*^					
191G-A (*rs*1801279)	Het (GA) vs. hom wt (GG)	Taiwan (1 study)	NR	Data excluded^*^					
		Turkey (1 study)	NR	Data excluded^*^					
		All (0 studies)		NA	NA	NA	NA
	Hom MT (AA) vs. hom wt (GG)	Taiwan (1 study)	NR	Data excluded^*^					
		Turkey (1 study)	NR	Data excluded^*^					
		All (0 studies)		NA	NA	NA	NA
282C-T (*rs*1041983)	Het (CT) vs. hom wt (CC)	China (1 study)	NR	1.28 (0.67–2.44)	65	98	NA
		Taiwan (1 study)	NR	0.50 (0.06–4.06)	70	284	NA
		Indonesia (1 study)	100% Indonesian	1.25 (0.51–3.05)	27	148	NA
		Brazil (1 study)	NR	1.67 (0.56–5.00)	14	216	NA
		All (4 studies)		1.27 (0.80, 2.02)	176	746	0.0
	Hom MT (TT) vs. hom wt (CC)	China (1 study)	NR	7.00 (2.89–16.98)	60	51	NA
		Taiwan (1 study)	NR	1.33 (0.05–32.91)	69	277	NA
		Indonesia (1 study)	100% Indonesian	3.41 (1.38–8.40)	31	94	NA
		Brazil (1 study)	NR	2.07 (0.59–7.25)	12	185	NA
		All (4 studies)		3.95 (2.21, 7.05)	172	607	5.5
341T-C (*rs*1801280)	Het (TC) vs. hom wt (TT)	China (1 study)	NR	1.63 (0.45–5.94)	101	107	NA
		Taiwan (2 studies)	NR	1.26 (0.58–2.75)	114	376	0.0
		Indonesia (1 study)	100% Indonesian	1.13 (0.54–2.35)	49	188	NA
		Brazil (1 study)	NR	0.66 (0.18–2.42)	10	187	NA
		All (5 studies)		1.15 (0.72–1.82)	274	858	0.0
	Hom MT (CC) vs. hom wt (TT)	China (1 study)	NR	Data excluded^*^					
		Taiwan (2 studies)	NR	1.18 (0.08–16.93)	105	355	41.4
		Indonesia (1 study)	100% Indonesian	1.32 (0.13–13.01)	38	149	NA
		Brazil (1 study)	NR	1.75 (0.50–6.13)	12	122	NA
		All (4 studies)		1.54 (0.58, 4.04)	155	626	0.0
481C-T (*rs*1799929)	Het (CT) vs. hom wt (CC)	China (3 studies)	1 study, 100% Chinese; 2 studies, NR	1.66 (1.11–2.48)	259	2027	0.0
		Taiwan (1 study)	NR	4.12 (0.25–66.63)	70	285	NA
		Indonesia (1 study)	100% Indonesian	1.01 (0.47–2.14)	49	188	NA
		India (1 study)	NR	1.82 (0.89–3.71)	39	154	NA
		Tunisia (1 study)	NR	1.33 (0.29–6.06)	8	42	NA
		Turkey (1 study)	NR	2.17 (0.88–5.36)	28	63	NA
		Brazil (1 study)	NR	0.44 (0.14–1.37)	14	216	NA
		All (9 studies)		1.48 (1.12, 1.97)	467	2975	0.0
	Hom MT (TT) vs. hom wt (CC)	China (3 studies)^†^	1 study, 100% Chinese; 2 studies, NR	0.81 (0.19–3.41)	41	1155	NA
		Taiwan (1 study)	NR	Data excluded^*^					
		Indonesia (1 study)	100% Indonesian	1.28 (0.13–12.66)	39	149	NA
		India (1 study)	NR	5.38 (1.99–14.49)	27	97	NA
		Tunisia (1 study)	NR	3.60 (0.83–15.57)	10	34	NA
		Turkey (1 study)	NR	0.93 (0.17–5.08)	14	46	NA
		Brazil (1 study)	NR	1.19 (0.34–4.09)	13	132	NA
		All (6 studies)		1.91 (0.93, 3.92)	144	1613	34.1
499G-A	Het (GA) vs. hom wt (GG)	China (1 study)	NR	0.21 (0.01–4.38)	101	107	NA
	Hom MT (AA) vs. hom wt (GG)	China (1 study)	NR	Data excluded^*^					
590G-A (*rs*1799930)	Het (GA) vs. hom wt (GG)	China (3 studies)	1 study, 100% Chinese; 2 studies, NR	1.19 (0.86–1.66)	236	1921	15.6
		Taiwan (2 studies)	NR	1.16 (0.74–1.82)	104	356	0.0
		South Korea (1 study)	NR	1.99 (1.06–3.74)	57	145	NA
		Indonesia (1 study)	100% Indonesian	1.17 (0.58–2.36)	38	173	NA
		India (1 study)	NR	1.38 (0.70–2.72)	45	137	NA
		Tunisia (1 study)	NR	0.77 (0.22–2.77)	12	50	NA
		Turkey (1 study)	NR	2.63 (1.00–6.87)	24	67	NA
		Brazil (1 study)	NR	2.36 (0.27–20.76)	18	247	NA
		All (11 studies)		1.30 (1.06, 1.59)	534	3096	0.0
	Hom MT (AA) vs. hom wt (GG)	China (3 studies)	1 study, 100% Chinese; 2 studies, NR	1.63 (0.66–4.00)	165	1356	58.1
		Taiwan (2 studies)	NR	1.52 (0.68–3.40)	74	250	0.0
		South Korea (1 study)	NR	5.26 (1.61–17.26)	39	107	NA
		Indonesia (1 study)	100% Indonesian	3.29 (1.34–8.08)	29	102	NA
		India (1 study)	NR	0.64 (0.22–1.88)	25	100	NA
		Tunisia (1 study)	NR	3.71 (0.44–31.26)	9	28	NA
		Turkey (1 study)	NR	9.11 (1.91–43.46)	15	44	NA
		Brazil (1 study)	NR	1.25 (0.07–23.62)	17	246	NA
		All (11 studies)		2.05 (1.24–3.40)	373	2233	47.7
803A-G (*rs*1208)	Het (AG) vs. hom wt (AA)	China (1 study)	NR	1.63 (0.45–5.94)	101	107	NA
		Taiwan (1 study)	NR	1.36 (0.14–13.30)	70	285	NA
		Indonesia (1 study)	100% Indonesian	1.15 (0.55–2.41)	49	187	NA
		Brazil (1 study)	NR	0.82 (0.27–2.52)	13	219	NA
		All (4 studies)		1.14 (0.67–1.96)	233	798	0.0
	Hom MT (GG) vs. hom wt (AA)	China (1 study)	NR	Data excluded^†^					
		Taiwan (1 study)	NR	Data excluded^†^					
		Indonesia (1 study)	100% Indonesian	0.99 (0.11–9.09)	38	150	NA
		Brazil (1 study)	NR	2.32 (0.69–7.78)	12	140	NA
		All (2 studies)		1.90 (0.66–5.52)	50	290	0.0%
857G-A (*rs*1799931)	Het (GA) vs. hom wt (GG)	China (3 studies)	1 study, 100% Chinese; 2 studies, NR	1.28 (0.74–2.22)	254	2069	61.5
		Taiwan (2 studies)	NR	1.13 (0.70–1.82)	103	368	0.0
		South Korea (1 study)	NR	1.11 (0.56–2.20)	65	150	NA
		Indonesia (1 study)	100% Indonesian	1.41 (0.72–2.75)	49	190	NA
		Tunisia (1 study)	NR	0.70 (0.03–15.34)	14	52	NA
		Turkey (1 study)	NR	3.39 (0.84–13.67)	29	69	NA
		Brazil (1 study)	NR	2.19 (0.73–6.55)	17	250	NA
		All (10 studies)		1.30 (1.03–1.64)	531	3148	0.9
	Hom MT (AA) vs. hom wt (GG)	China (3 studies)	1 study, 100% Chinese; 2 studies, NR	0.98 (0.38–2.51)	184	1677	0.0
		Taiwan (2 studies)	NR	5.05 (0.47–54.88)	82	268	74.2
		South Korea (1 study)	NR	1.18 (0.10–13.36)	50	118	NA
		Indonesia (1 study)	100% Indonesian	4.31 (0.26–70.80)	33	139	NA
		Tunisia (1 study)	NR	Data excluded^*^			
		Turkey (1 study)	NR	2.71 (0.16–45.03)	25	66	NA
		Brazil (1 study)	NR	8.75 (0.74–103.44)	13	212	NA
		All (9 studies)		1.99 (1.02–3.91)	387	2480	11.3
*rs*1495741	Het (AG) vs. hom wt (AA)	Taiwan (1 study)	NR	0.19 (0.07–0.52)	19	249	NA
	Hom MT (GG) vs. hom wt (AA)	Taiwan (1 study)	NR	0.07 (0.01–0.56)	14	152	NA
*rs*4646244	Het (TA) vs. hom wt (TT)	South Korea (1 study)	NR	2.03 (1.09–3.78)	57	152	NA
	Hom MT (AA) vs. hom wt (TT)	South Korea (1 study)	NR	4.06 (1.36–12.13)	37	110	NA
Rs4646267	Het (AG) vs. hom wt (AA)	South Korea (1 study)	NR	0.50 (0.25–0.98)	52	127	NA
	Hom MT (GG) vs. hom wt (AA)	South Korea (1 study)	NR	0.63 (0.27–1.45)	35	66	NA

^*^ Due to zero patients in one of the genotype groups.

^†^ Data from two of the three Chinese studies were excluded due to zero counts.

SNP = single nucleotide polymorphism OR = odds ratio; CI = confidence interval; het = heterozygous genotype; hom wt = homozygous wild-type; NR = not reported; NA = not applicable; hom MT = homozygous mutant-type.

**Table 1:** (*continued*)

*NAT2* SNP	Comparison	Country (number of studies)	Ethnicity	OR (95%CI)	Cases *n*	Controls *n*	*I*^2^
190C-T	Het (CT) vs. hom wt (CC)	China (1 study)	NR	0.21 (0.01–4.38)	101	107	NA
	Hom MT (TT) vs. hom wt (CC)	China (1 study)	NR	Data excluded^*^					
191G-A (*rs*1801279)	Het (GA) vs. hom wt (GG)	Taiwan (1 study)	NR	Data excluded^*^					
		Turkey (1 study)	NR	Data excluded^*^					
		All (0 studies)		NA	NA	NA	NA
	Hom MT (AA) vs. hom wt (GG)	Taiwan (1 study)	NR	Data excluded^*^					
		Turkey (1 study)	NR	Data excluded^*^					
		All (0 studies)		NA	NA	NA	NA
282C-T (*rs*1041983)	Het (CT) vs. hom wt (CC)	China (1 study)	NR	1.28 (0.67–2.44)	65	98	NA
		Taiwan (1 study)	NR	0.50 (0.06–4.06)	70	284	NA
		Indonesia (1 study)	100% Indonesian	1.25 (0.51–3.05)	27	148	NA
		Brazil (1 study)	NR	1.67 (0.56–5.00)	14	216	NA
		All (4 studies)		1.27 (0.80, 2.02)	176	746	0.0
	Hom MT (TT) vs. hom wt (CC)	China (1 study)	NR	7.00 (2.89–16.98)	60	51	NA
		Taiwan (1 study)	NR	1.33 (0.05–32.91)	69	277	NA
		Indonesia (1 study)	100% Indonesian	3.41 (1.38–8.40)	31	94	NA
		Brazil (1 study)	NR	2.07 (0.59–7.25)	12	185	NA
		All (4 studies)		3.95 (2.21, 7.05)	172	607	5.5
341T-C (*rs*1801280)	Het (TC) vs. hom wt (TT)	China (1 study)	NR	1.63 (0.45–5.94)	101	107	NA
		Taiwan (2 studies)	NR	1.26 (0.58–2.75)	114	376	0.0
		Indonesia (1 study)	100% Indonesian	1.13 (0.54–2.35)	49	188	NA
		Brazil (1 study)	NR	0.66 (0.18–2.42)	10	187	NA
		All (5 studies)		1.15 (0.72–1.82)	274	858	0.0
	Hom MT (CC) vs. hom wt (TT)	China (1 study)	NR	Data excluded^*^					
		Taiwan (2 studies)	NR	1.18 (0.08–16.93)	105	355	41.4
		Indonesia (1 study)	100% Indonesian	1.32 (0.13–13.01)	38	149	NA
		Brazil (1 study)	NR	1.75 (0.50–6.13)	12	122	NA
		All (4 studies)		1.54 (0.58, 4.04)	155	626	0.0
481C-T (*rs*1799929)	Het (CT) vs. hom wt (CC)	China (3 studies)	1 study, 100% Chinese; 2 studies, NR	1.66 (1.11–2.48)	259	2027	0.0
		Taiwan (1 study)	NR	4.12 (0.25–66.63)	70	285	NA
		Indonesia (1 study)	100% Indonesian	1.01 (0.47–2.14)	49	188	NA
		India (1 study)	NR	1.82 (0.89–3.71)	39	154	NA
		Tunisia (1 study)	NR	1.33 (0.29–6.06)	8	42	NA
		Turkey (1 study)	NR	2.17 (0.88–5.36)	28	63	NA
		Brazil (1 study)	NR	0.44 (0.14–1.37)	14	216	NA
		All (9 studies)		1.48 (1.12, 1.97)	467	2975	0.0
	Hom MT (TT) vs. hom wt (CC)	China (3 studies)^†^	1 study, 100% Chinese; 2 studies, NR	0.81 (0.19–3.41)	41	1155	NA
		Taiwan (1 study)	NR	Data excluded^*^					
		Indonesia (1 study)	100% Indonesian	1.28 (0.13–12.66)	39	149	NA
		India (1 study)	NR	5.38 (1.99–14.49)	27	97	NA
		Tunisia (1 study)	NR	3.60 (0.83–15.57)	10	34	NA
		Turkey (1 study)	NR	0.93 (0.17–5.08)	14	46	NA
		Brazil (1 study)	NR	1.19 (0.34–4.09)	13	132	NA
		All (6 studies)		1.91 (0.93, 3.92)	144	1613	34.1
499G-A	Het (GA) vs. hom wt (GG)	China (1 study)	NR	0.21 (0.01–4.38)	101	107	NA
	Hom MT (AA) vs. hom wt (GG)	China (1 study)	NR	Data excluded^*^					
590G-A (*rs*1799930)	Het (GA) vs. hom wt (GG)	China (3 studies)	1 study, 100% Chinese; 2 studies, NR	1.19 (0.86–1.66)	236	1921	15.6
		Taiwan (2 studies)	NR	1.16 (0.74–1.82)	104	356	0.0
		South Korea (1 study)	NR	1.99 (1.06–3.74)	57	145	NA
		Indonesia (1 study)	100% Indonesian	1.17 (0.58–2.36)	38	173	NA
		India (1 study)	NR	1.38 (0.70–2.72)	45	137	NA
		Tunisia (1 study)	NR	0.77 (0.22–2.77)	12	50	NA
		Turkey (1 study)	NR	2.63 (1.00–6.87)	24	67	NA
		Brazil (1 study)	NR	2.36 (0.27–20.76)	18	247	NA
		All (11 studies)		1.30 (1.06, 1.59)	534	3096	0.0
	Hom MT (AA) vs. hom wt (GG)	China (3 studies)	1 study, 100% Chinese; 2 studies, NR	1.63 (0.66–4.00)	165	1356	58.1
		Taiwan (2 studies)	NR	1.52 (0.68–3.40)	74	250	0.0
		South Korea (1 study)	NR	5.26 (1.61–17.26)	39	107	NA
		Indonesia (1 study)	100% Indonesian	3.29 (1.34–8.08)	29	102	NA
		India (1 study)	NR	0.64 (0.22–1.88)	25	100	NA
		Tunisia (1 study)	NR	3.71 (0.44–31.26)	9	28	NA
		Turkey (1 study)	NR	9.11 (1.91–43.46)	15	44	NA
		Brazil (1 study)	NR	1.25 (0.07–23.62)	17	246	NA
		All (11 studies)		2.05 (1.24–3.40)	373	2233	47.7
803A-G (*rs*1208)	Het (AG) vs. hom wt (AA)	China (1 study)	NR	1.63 (0.45–5.94)	101	107	NA
		Taiwan (1 study)	NR	1.36 (0.14–13.30)	70	285	NA
		Indonesia (1 study)	100% Indonesian	1.15 (0.55–2.41)	49	187	NA
		Brazil (1 study)	NR	0.82 (0.27–2.52)	13	219	NA
		All (4 studies)		1.14 (0.67–1.96)	233	798	0.0
	Hom MT (GG) vs. hom wt (AA)	China (1 study)	NR	Data excluded^†^					
		Taiwan (1 study)	NR	Data excluded^†^					
		Indonesia (1 study)	100% Indonesian	0.99 (0.11–9.09)	38	150	NA
		Brazil (1 study)	NR	2.32 (0.69–7.78)	12	140	NA
		All (2 studies)		1.90 (0.66–5.52)	50	290	0.0%
857G-A (*rs*1799931)	Het (GA) vs. hom wt (GG)	China (3 studies)	1 study, 100% Chinese; 2 studies, NR	1.28 (0.74–2.22)	254	2069	61.5
		Taiwan (2 studies)	NR	1.13 (0.70–1.82)	103	368	0.0
		South Korea (1 study)	NR	1.11 (0.56–2.20)	65	150	NA
		Indonesia (1 study)	100% Indonesian	1.41 (0.72–2.75)	49	190	NA
		Tunisia (1 study)	NR	0.70 (0.03–15.34)	14	52	NA
		Turkey (1 study)	NR	3.39 (0.84–13.67)	29	69	NA
		Brazil (1 study)	NR	2.19 (0.73–6.55)	17	250	NA
		All (10 studies)		1.30 (1.03–1.64)	531	3148	0.9
	Hom MT (AA) vs. hom wt (GG)	China (3 studies)	1 study, 100% Chinese; 2 studies, NR	0.98 (0.38–2.51)	184	1677	0.0
		Taiwan (2 studies)	NR	5.05 (0.47–54.88)	82	268	74.2
		South Korea (1 study)	NR	1.18 (0.10–13.36)	50	118	NA
		Indonesia (1 study)	100% Indonesian	4.31 (0.26–70.80)	33	139	NA
		Tunisia (1 study)	NR	Data excluded^*^			
		Turkey (1 study)	NR	2.71 (0.16–45.03)	25	66	NA
		Brazil (1 study)	NR	8.75 (0.74–103.44)	13	212	NA
		All (9 studies)		1.99 (1.02–3.91)	387	2480	11.3
*rs*1495741	Het (AG) vs. hom wt (AA)	Taiwan (1 study)	NR	0.19 (0.07–0.52)	19	249	NA
	Hom MT (GG) vs. hom wt (AA)	Taiwan (1 study)	NR	0.07 (0.01–0.56)	14	152	NA
*rs*4646244	Het (TA) vs. hom wt (TT)	South Korea (1 study)	NR	2.03 (1.09–3.78)	57	152	NA
	Hom MT (AA) vs. hom wt (TT)	South Korea (1 study)	NR	4.06 (1.36–12.13)	37	110	NA
Rs4646267	Het (AG) vs. hom wt (AA)	South Korea (1 study)	NR	0.50 (0.25–0.98)	52	127	NA
	Hom MT (GG) vs. hom wt (AA)	South Korea (1 study)	NR	0.63 (0.27–1.45)	35	66	NA

^*^ Due to zero patients in one of the genotype groups.

^†^ Data from two of the three Chinese studies were excluded due to zero counts.

SNP = single nucleotide polymorphism OR = odds ratio; CI = confidence interval; het = heterozygous genotype; hom wt = homozygous wild-type; NR = not reported; NA = not applicable; hom MT = homozygous mutant-type.

Results were relatively homogeneous between studies for most comparisons, except for the comparison between homozygous mutant-type and homozygous wt for the 590G-A SNP (*I*^2^ =47.7%). This moderate heterogeneity may have been due to the variable distribution of genotypes in different geographic areas (Table 1; Appendix Figure A.5).

#### NAT2 variants and other toxicity outcomes

A summary of all data for the association between *NAT2* variants and toxicity outcomes (other than hepatotoxicity) is provided in Table 2. Each reported result is based on data from a single study because there were no comparisons where more than one study provided data (Table 2).

**Table 2:** Summary of results for all toxicity outcomes other than hepatotoxicity

Outcome	Variant	Study	Country	Ethnicity	Comparison	OR (95%CI)	Cases *n*	Controls *n*
Peripheral neuropathy	Acetylator status	Azuma, 2013	Japan	NR	Intermediate vs. rapid	1.36 (0.32–5.75)	8	104
					Slow vs. rapid	4.29 (0.66–27.8)	6	67
	191G-A (rs1801279)	Dhoro, 2013	Zimbabwe	NR	Het (GA) vs. hom wt (GG)	0.69 (0.33–1.41)	102	56
					Hom MT (AA) vs. hom wt (GG)	2.48 (0.12–53.02)	79	38
	341T-C (rs1801280)	Dhoro, 2013	Zimbabwe	NR	Het (TC) vs. hom wt (TT)	1.01 (0.50–2.07)	84	48
					Hom MT (CC) vs. hom wt (TT)	1.34 (0.32–5.62)	54	30
Adverse DIH outcome	Acetylator status	Bose, 2011	India	NR	Slow vs. rapid/intermediate	3.31 (1.03–10.62)	16	202
ADRs	Acetylator status	Costa, 2012	Brazil	84% Black/mixed race, 16% other	Slow vs. rapid/intermediate	3.20 (1.31–7.80)	40	47
Skin rash	Acetylator status	Higuchi, 2007	Japan	NR	Intermediate vs. rapid	0.83 (0.32–2.19)	22	68
					Slow vs. rapid	1.21 (0.27–5.46)	15	41
Eosinophilia	Acetylator status	Higuchi, 2007	Japan	NR	Intermediate vs. rapid	1.44 (0.60–3.45)	31	59
					Slow vs. rapid	0.98 (0.22–4.35)	17	39
ATD-induced MPE	R197Q (590G-A, *rs*1799930)	Kim, 2011 (GI: KIM)	South Korea	NR	Hom MT (AA) or het (GA) vs. hom wt (GG)	0.96 (0.50–1.84)	58	150
	G286E (857G-A, *rs*1799931)	Kim, 2011 (GI: KIM)	South Korea	NR	Hom MT (AA) or het (GA) vs. hom wt (GG)	1.65 (0.86–3.18)	59	152
	-9796 T-A (*rs*4646244)	Kim, 2011 (GI: KIM)	South Korea	NR	Hom MT (AA) or het (TA) vs. hom wt (TT)	1.08 (0.59–2.00)	62	159
	-9601A-G (*rs*4646267)	Kim, 2011 (GI: KIM)	South Korea	NR	Hom MT (GG) or het (AG) vs. hom wt (AA)	0.65 (0.33–1.27)	61	159
Gastrointestinal ADRs	Acetylator status	Possuelo, 2008(GI: POSSUELO)	Brazil	57% White	Slow vs. rapid/intermediate	1.18 (0.51–2.70)	33	207

OR=odds ratio; CI=confidence interval; NR=not reported; HET=heterozygous genotype; HOM WT=homozygous wild-type; HOM mt=homozygous mutant-type; DIH=drug-induced hepatotoxicity; ADR=adverse drug reaction; ATD = anti-tuberculosis drug; MPE = maculopapular eruption; GI = group identifier.

For peripheral neuropathy, no significant associations were reported for either of the pairwise comparisons conducted for acetylator status, 191G-A or 341T-C. Similarly, for skin rash and eosinophilia, the pairwise comparisons for acetylator status demonstrated no significant effects. None of the SNPs investigated by Kim et al. had a significant effect on anti-tuberculosis drug-induced maculopapular eruption.^43^ Slow acetylators were significantly more likely to experience adverse drug-induced hepatotoxicity outcomes (definition unclear; OR 3.31, 95%CI 1.03–10.62), and ADRs (defined as at least one of the following: gastric, joint, neuromuscular or skin reactions, hepatotoxicity; OR 3.20, 95%CI 1.31–7.80) compared with rapid or intermediate acetylators. However, slow acetylator status was not found to increase the risk of gastrointestinal ADRs.

## DISCUSSION

There is substantial evidence for the association between *NAT2* variants and anti-tuberculosis drug-related toxicity outcomes, as previously identified and as our systematic review confirmed. However, we established that performing robust synthesis of this evidence is challenging due to the variability between studies in terms of how participants are classified according to genotype; choice and definition of outcomes and variants to investigate; ethnicity of participants; and methodological quality. In conducting our review, we carefully considered these challenges, stratifying meta-analyses by genetic variants, genotype contrasts and outcomes. We also stratified further by the country where the study was conducted as a proxy for ethnicity, which has not been widely reported. We supplemented our data synthesis with a rigorous assessment of the methodological quality of included studies.

### Meta-analyses

Where possible, meta-analyses were undertaken to improve the power to estimate genetic effects. We found that slow/intermediate acetylators were significantly more likely to experience hepatotoxicity than rapid acetylators. This result is consistent with the findings of several meta-analyses,^12–15^ but not consistent with the meta-analysis reported by Sun et al.,^6^ who did not identify a significant association between slow acetylator status and hepatotoxicity. However, the search date for Sun et al.^6^ (May 2007) is several years earlier than the search dates for the other meta-analyses, and many relevant studies have been published in recent years. As more studies are published, the power to detect a statistically significant association increases.

Meta-analyses on individual SNPs of the *NAT2* gene have not been published, so our results add to the existing knowledge of the association between *NAT2* variants and hepatotoxicity.

INH remains an essential drug in the treatment of active TB and is the mainstay of chemoprophylaxis in latent tuberculous infection (LTBI), an intervention that is being rapidly expanded in recent strategies to eliminate TB as a public health problem. The global use of INH will therefore greatly increase worldwide in the coming decade. While transaminase testing is a readily available biomarker of possible ATDH, baseline values have modest predictive value and routine monitoring is not generally recommended. Where slow acetylator status is common, pharmacogenetic testing could make a clinically useful contribution to risk stratification for ATDH. However, the need for testing of a relatively large panel of SNPs and the current lack of a clear substitute to INH for LTBI chemoprophylaxis mean that such a strategy may not be cost-effective or feasible. Studies investigating the cost-effectiveness and/or feasibility of such a strategy would be beneficial. Nevertheless, based on the nearly three-fold increased risk of ATDH in slow acetylators observed in this review, pharmacogenetic epidemiology should certainly be a factor in national policymaking on the need for transaminase monitoring during treatment of active TB and LTBI locally.

### Quality assessment

The quality of included studies varied, with some areas of concern. Most studies were significantly smaller than typically required to provide sufficient power,^20^ and the reader was left unaware of the likelihood of false-negatives in all studies due to the lack of reported a priori power calculations. The fact that no studies described checking that missing data were missing at random is also a concern; missing genotype data are unlikely to be missing at random because heterozygotes are notoriously more difficult to call than homozygotes.^20^ Few studies reported testing of HWE, which can highlight genotyping errors, population stratification and other problems.^20^ Furthermore, in studies that did not adjust for treatment adherence, the proportion of variability explained by genetic variants may have been underestimated.^20^

As the quality assessment was qualitative rather than quantitative, it was not possible to exclude studies from meta-analyses based on a single summary score. Although we identified issues of concern relating to some of the quality criteria, we did not identify any studies that were thought to be of particularly poor quality overall, so we did not deem it necessary to exclude any single study in sensitivity analyses.

### Limitations

Most included studies did not report the ethnic background of participants. We therefore performed analyses stratified by the country in which the study was conducted as a proxy variable for ethnicity. It is clear that this approach is not ideal as the population of any given country is often ethnically diverse. However, stratifying by country was deemed the most suitable approach in the absence of definitive information on ethnicity.

An additional challenge was identifying distinct patient cohorts from the included articles. If multiple articles report data for the same patient cohort, data for this patient cohort must only be included in meta-analysis once, otherwise a unit-of-analysis error occurs.^18^ We found that it was often not possible to determine from the articles alone whether the patient cohorts were identical. We contacted several study authors for clarification. For two articles,^51^,^68^ we did not receive a response and, consequently, data from the older article^68^ were excluded from a meta-analysis to which both articles contributed data. If the two articles reported data for two distinct cohorts, then information would have been lost by excluding one article. Furthermore, there may have been cases of multiple articles reporting outcomes for the same cohorts that we did not identify; if this was the case, some patients may have been double-counted in the meta-analyses.

There was considerable variability in the definitions of hepatotoxicity in the included studies, which introduced heterogeneity into the meta-analyses. Jorgensen et al.^71^ and Contopoulos-Ioannidis et al.^72^ made similar observations about the variability of definitions of outcomes across pharmacogenetics studies. If outcome definitions were more consistent between pharmacogenetic studies, the amount of heterogeneity observed in meta-analyses would have been reduced.

Finally, an important limitation of the systematic review was a lack of evidence from studies conducted in Africa. There is a great deal of *NAT2* diversity across Africa,^73^ where TB is endemic, but there has been little mapping of pharmacogenomic polymorphisms in African populations. Only four studies included in this review were conducted in Africa. The vast majority of evidence included in this review is therefore not representative of the global population most affected by TB.

### Recommendations for authors of pharmacogenetic studies

We made several recommendations regarding the reporting of future pharmacogenetic studies to facilitate the conduct of high-quality systematic reviews and meta-analyses, and thus improve the power to detect genetic associations.

1 Report the number of patients in each genotype group;
2 Report outcomes for each genotype group separately (i.e., number of events for dichotomous outcomes, and mean and standard deviation values for continuous outcomes);
3 Report the *rs* number of each genotyped SNP;
4 Report the ethnicity of included patients;
5 If a study includes more than one ethnic group, provide the summary data specified in 1) and 2) per ethnic group;
6 Provide the reference to the published protocol;
7 Provide information on patient cohort overlap;
8 Report full details of all variants and outcomes investigated, and of all analyses undertaken;
9 Consensus should be reached between experts in specific areas of research on the definitions of outcomes that are commonly reported in pharmacogenetic studies of a particular treatment.

We also recommend that articles adhere to the criteria of the quality assessment tool^20^ as improvement in the methodological quality of studies included in meta-analyses would in turn improve the strength of the evidence synthesised in meta-analyses. Furthermore, we recommend that STREGA reporting guidelines are referred to, which provide guidance on the reporting of genetic association studies in general.^74^

## CONCLUSION

This review showed that slow/intermediate acetylators were significantly more likely to experience hepatotoxicity than rapid acetylators. Therefore, pharmacogenetic testing may be useful in clinical practice in terms of risk stratification for ATDH during treatment of TB. However, more studies are needed to overcome the reported methodological limitations and to assess if this strategy might be feasible and cost-effective.

## Figures and Tables

**Figure A.2:**
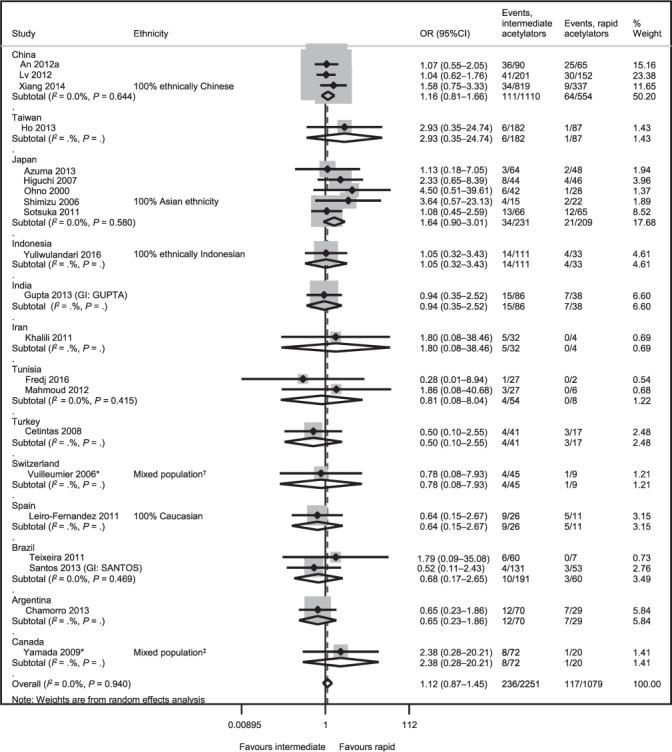
Sensitivity analysis 1: Intermediate vs. rapid acetylator status for the outcome of hepatotoxicity. ^*^ Conducted in LTBI populations. ^†^ Caucasian (n = 38, 43%), Hispanic (n = 8, 9%), African (n = 22, 25%), South American (n = 15, 17%), Asian (n = 5, 6%), Middle Eastern (n =1, 1%). ^‡^ Asian (n =72, 42%), Caucasian (n =49, 29%), South Asian (n =22, 13%), Hispanic (n =7, 4%), Middle Eastern (n=8, 5%), First nations (n=5, 3%), Other/mixed/unknown: (n=7, 4%). OR=odds ratio; CI=confidence interval; GI = group identifier; LTBI = latent tuberculous infection.

**Figure A.3:**
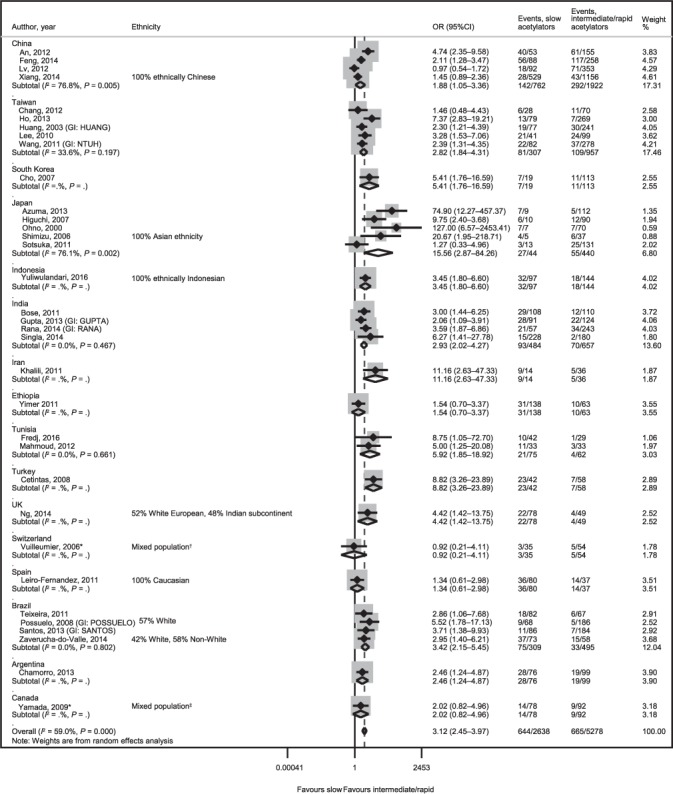
Sensitivity analysis 2: Slow vs. rapid/intermediate acetylator status for the outcome of hepatotoxicity. ^*^ Conducted in LTBI populations. ^†^ Caucasian (n=38, 43%), Hispanic (n=8, 9%), African (n=22, 25%), South American (n=15, 17%), Asian (n=5, 6%), Middle Eastern (n =1, 1%). ^‡^Asian (n =72, 42%), Caucasian (n =49, 29%), South Asian (n =22, 13%), Hispanic (n =7, 4%), Middle Eastern (n=8, 5%), First nations (n=5, 3%), Other/mixed/unknown: (n=7, 4%). OR=odds ratio; CI=confidence interval; GI = group identifier; LTBI = latent tuberculous infection.

**Figure A.5:**
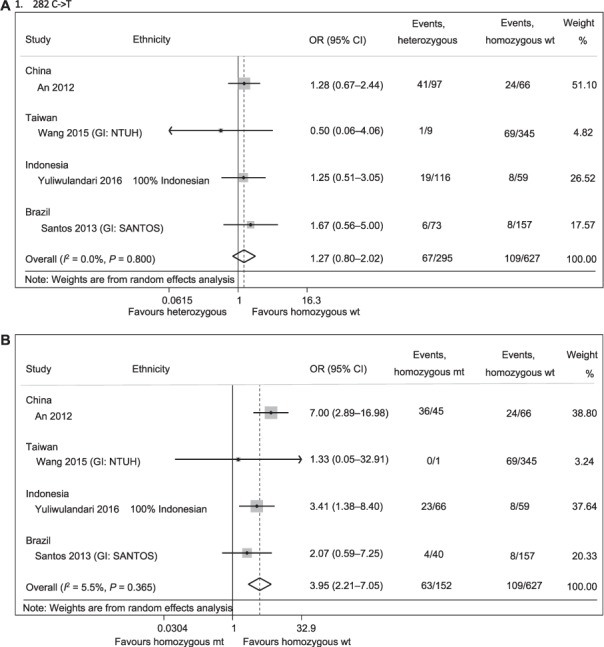
NAT2 SNPs and hepatotoxicity meta-analyses: heterozygous genotype (CT) vs. homozygous wt genotype (CC) for the outcome of hepatotoxicity. A) Heterozygous genotype (CT) vs. homozygous wt genotype (CC) for the outcome of hepatotoxicity. B) Homozygous mutant genotype (TT) vs. homozygous wt genotype (CC) for the outcome of hepatotoxicity. C) Heterozygous genotype (TC) vs. homozygous wt genotype (TT) for the outcome of hepatotoxicity. D) Homozygous mutant genotype (CC) vs. homozygous wt genotype (TT) for the outcome of hepatotoxicity. E) Heterozygous genotype (CT) vs. homozygous wt genotype (CC) for the outcome of hepatotoxicity. F) Homozygous mutant genotype (TT) vs. homozygous wt genotype (CC) for the outcome of hepatotoxicity. G) Heterozygous genotype (GA) vs. homozygous wt genotype (GG) for the outcome of hepatotoxicity. H) Homozygous mutant genotype (AA) vs. homozygous wt genotype (GG) for the outcome of hepatotoxicity. I) Heterozygous genotype (GA) vs. homozygous wt genotype (AA) for the outcome of hepatotoxicity. J) Homozygous mutant genotype (GG) vs. homozygous wt genotype (AA) for the outcome of hepatotoxicity. K) Heterozygous genotype (GA) vs. homozygous wt genotype (GG) for the outcome of hepatotoxicity. L) Homozygous mutant genotype (AA) vs. homozygous wt genotype (GG) for the outcome of hepatotoxicity. OR = odds ratio; CI = confidence interval; GI =group identifier; wt =wild type; NAT2 =N-acetyltransferase 2; SNP =single-nucleotide polymorphism; mt = mutation.

**Figure A.5:**
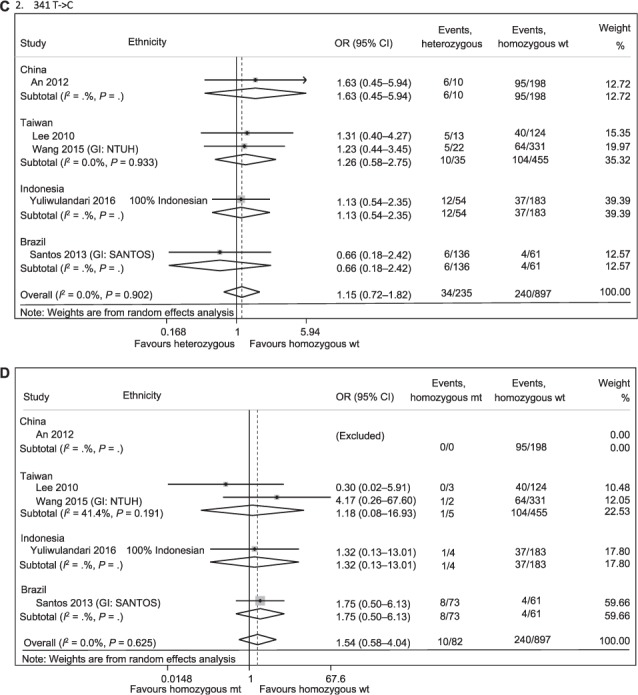
(*continued*)

**Figure A.5:**
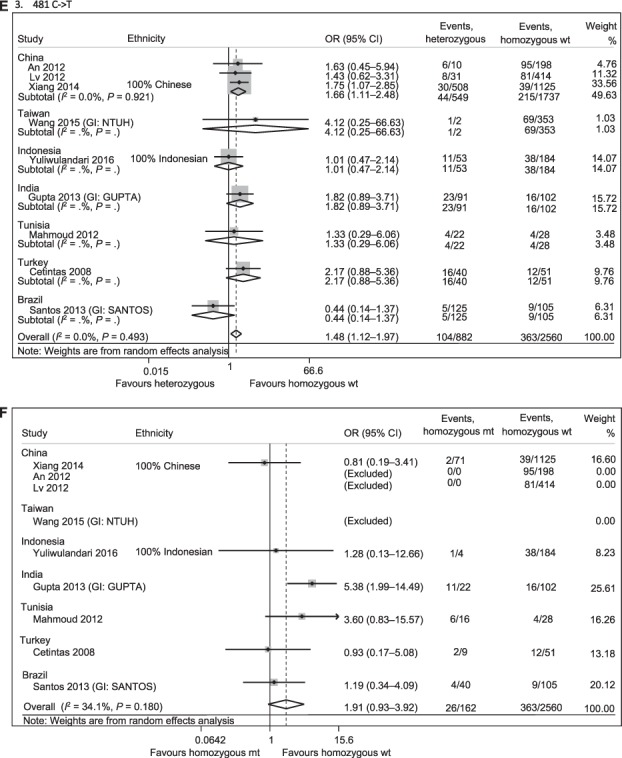
(*continued*)

**Figure A.5:**
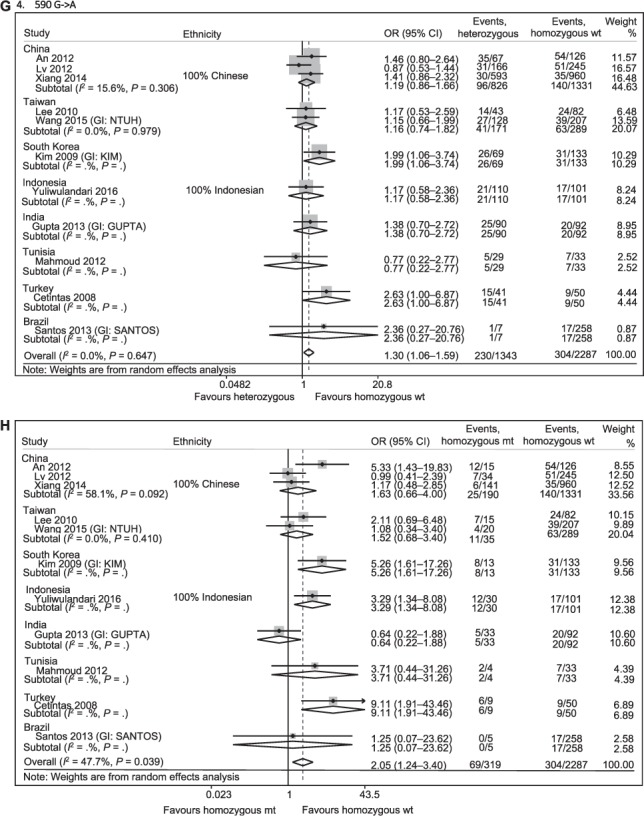
(*continued*)

**Figure A.5:**
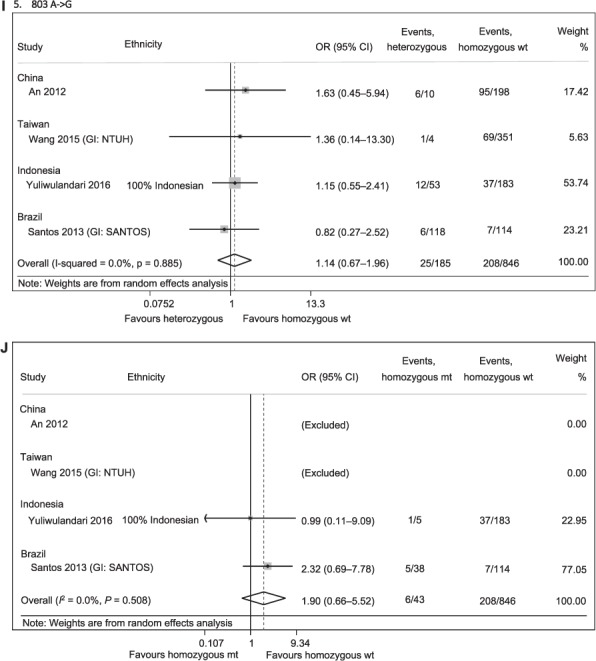
(*continued*)

**Figure A.5:**
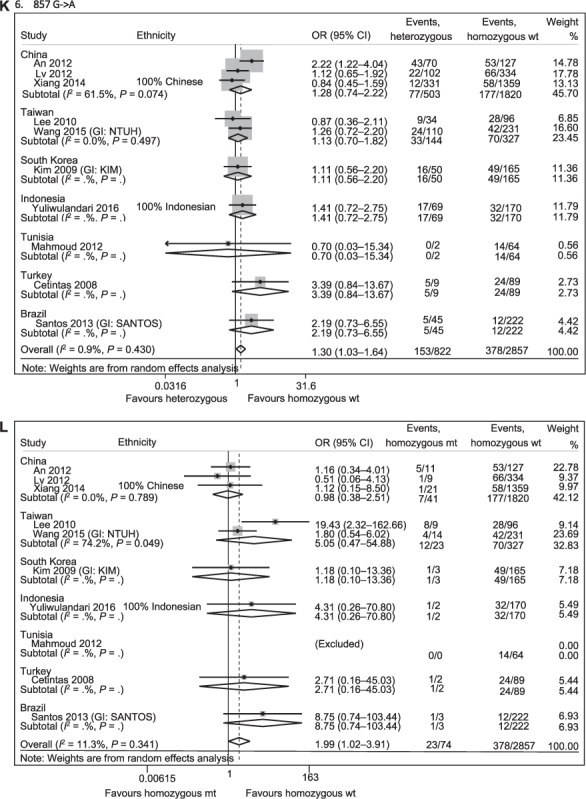
(*continued*)

## Footnotes

^*^ The appendix is available in the online version of this article, at http://www.ingentaconnect.com/content/iuatld/ijtld/2018/00000023/00000003/art000.....

## APPENDIX

**Table A.1:** Search history

Databases	Date searched	Number retrieved
MEDLINE (Ovid) and MEDLINE In-Process (Ovid)	3 March 2016	3029
EMBASE (Ovid)	3 March 2016	4778
PubMed	3 March 2016	379
Web of science	3 March 2016	421
Biosis	3 March 2016	328

**Table A.2:** Search strategies

A) Database: Web of science and Biosis			

Approximately 1 634 627		#10 OR #7 OR #6 OR #5 OR #4	
Approximately 9 565		**TITLE:** ((((Genetic or gene*) near/2 associat* near/2 (studies or study or analys*))))	
Approximately 93 935		#2 OR #1	
Approximately 382 014		**TITLE:** ((((TB or Tuberculosis* or Antitubercul*))))	
Approximately 219 324		**TITLE:** ((((gene* or genetic*) near/5 (mutat* or variant*))))	
Approximately 1 388 831		**TITLE:** ((((SNP or Genotyp* or Phenotyp* or Allele* or Pharmacogenet* or Pharmacogenom* or Polymorph*))))	
Approximately 38 430		**TITLE:** (((single* near/2 nucleotid* near/2 polymorph*)))	
Approximately 45 745		**TITLE:** ((((genetic* or gene*) near/3 (suscept* or predisposit* or anticipat*))))	
Approximately 47 961		**TITLE:** (((aminosalicylic acid or diarylquinoline* or ethambutol* or ethionamide* or isoniazid* or prothionamide* or pyrazinamide* or thioacetazone* or capreomycin* or cycloserine* or enviomycin* or rifabutin* or rifampin* or viomycin*)))	
Approximately 49 386		**TITLE:** ((((Antitubercul* or tuberculos* or TB) Near/4 (agent* or drug* or antibiotic* or medicine* or medication* or treatment*))))	

**B)** Database: Medline			

# ▴	Searches		Results

1	antitubercular agents/ or aminosalicylic acid/ or diarylquinolines/ or ethambutol/ or ethionamide/ or isoniazid/ or prothionamide/ or pyrazinamide/ or thioacetazone/ or antibiotics, antitubercular/ or capreomycin/ or cycloserine/ or enviomycin/ or rifabutin/ or rifampin/ or viomycin/		73 943
2	((Antitubercul* or tuberculos* or TB) adj4 (agent* or drug* or antibiotic* or medicine* or medication* or treatment*)).tw.		29 293
3	(aminosalicylic acid or diarylquinoline* or ethambutol* or ethionamide* or isoniazid* or prothionamide* or pyrazinamide* or thioacetazone* or capreomycin* or cycloserine* or enviomycin* or rifabutin* or rifampin* or viomycin*).tw.		26 053
4	1 or 2 or 3		93 357
5	Polymorphism, Genetic/		103 705
6	genetic predisposition to disease/ or anticipation, genetic/		101 390
7	Pharmacogenetics/		9 595
8	Genetic Association Studies/		14 210
9	((Genetic or gene*) adj2 associat* adj2 (studies or study or analys*)).tw.		4 883
10	((genetic* or gene*) adj3 (suscept* or predisposit* or anticipat*)).tw.		40 247
11	Polymorphism, Single Nucleotide/		77 811
12	(single* adj2 nucleotid* adj2 polymorph*).tw.		46 260
13	(SNP or Genotyp* or Phenotyp* or Allele* or Pharmacogenet* or Pharmacogenom* or Polymorph*).tw.		774 469
14	((gene* or genetic*) adj5 (mutat* or variant*)).tw.		182 197
15	Genotype/ or Phenotype/ or Alleles/		381 555
16	or/5–15		1 035 512
17	exp Tuberculosis/		175 110
18	(TB or Tuberculosis*).tw.		153 175
19	Antitubercul*.tw.		11 635
20	or/17–19		213 138
21	4 and 16 and 20		2 846
22	animal/ not human/		4 159 388
23	21 not 22		2 730

**C)** Database: Embase			

# ▴	Searches		Results

1	antitubercular agents/ or aminosalicylic acid/ or diarylquinolines/ or ethambutol/ or ethionamide/ or isoniazid/ or prothionamide/ or pyrazinamide/ or thioacetazone/ or antibiotics, antitubercular/ or capreomycin/ or cycloserine/ or enviomycin/ or rifabutin/ or rifampin/ or viomycin/		151 901
2	((Antitubercul* or tuberculos* or TB) adj4 (agent* or drug* or antibiotic* or medicine* or medication* or treatment*)).tw.		40 664
3	(aminosalicylic acid or diarylquinoline* or ethambutol* or ethionamide* or isoniazid* or prothionamide* or pyrazinamide* or thioacetazone* or capreomycin* or cycloserine* or enviomycin* or rifabutin* or rifampin* or viomycin*).tw.		34 743
4	1 or 2 or 3		172 588
5	Polymorphism, Genetic/		102 257
6	genetic predisposition to disease/ or anticipation, genetic/		97 585
7	Pharmacogenetics/		17 431
8	Genetic Association Studies/		876
9	((Genetic or gene*) adj2 associat* adj2 (studies or study or analys*)).tw.		7 890
10	((genetic* or gene*) adj3 (suscept* or predisposit* or anticipat*)).tw.		61 544
11	Polymorphism, Single Nucleotide/		98 303
12	(single* adj2 nucleotid* adj2 polymorph*).tw.		75 841
13	(SNP or Genotyp* or Phenotyp* or Allele* or Pharmacogenet* or Pharmacogenom* or Polymorph*).tw.		1 171 894
14	((gene* or genetic*) adj5 (mutat* or variant*)).tw.		294 715
15	Genotype/ or Phenotype/ or Alleles/		777 386
16	or/5–15		1 548 879
17	exp Tuberculosis/		197 008
18	(TB or Tuberculosis*).tw.		187 590
19	Antitubercul*.tw.		16 330
20	or/17–19		253 048
21	4 and 16 and 20		5 380
22	animal/ not human/		1 357 016
23	21 not 22		5 360
24	limit 23 to em=188300-201608		4 778

**D)** Database: PubMed			

#1	Search (((Antitubercul* or tuberculos* or TB))) AND ((agent* or drug* or antibiotic* or medicine* or medication* or treatment*))		124 242
#2	Search ((aminosalicylic acid or diarylquinoline* or ethambutol* or ethionamide* or isoniazid* or prothionamide* or pyrazinamide* or thioacetazone* or capreomycin* or cycloserine* or enviomycin* or rifabutin* or rifampin* or viomycin*))		49 591
#3	Search (#1 or #2)		151 329
#4	Search ((((Genetic or gene*) near/2 near/2 ))) AND associat*) AND ((studies or study or analys*))		3 922
#5	Search (((genetic* or gene*))) AND ((suscept* or predisposit* or anticipat*))		235 548
#6	Search ((single*) AND nucleotid*) AND polymorph*		98 071
#7	Search ((SNP or Genotyp* or Phenotyp* or Allele* or Pharmacogenet* or Pharmacogenom* or Polymorph*))		997 538
#8	Search (((gene* or genetic*))) AND ((mutat* or variant*))		743 819
#9	Search (#4 or #5 or #6 or #7 or #8)		1 553 428
#10	Search (((((TB or Tuberculosis* or Antitubercul*)))))		251 923
#11	Search (#3 and #9 and #10)		7 671
#12	Search (“2015/08/01”[Date - Entrez] : “3000”[Date - Entrez])		658 085
#13	Search (#11 and #12)		379

**Table A.2:** Search strategies

A) Database: Web of science and Biosis			

Approximately 1 634 627		#10 OR #7 OR #6 OR #5 OR #4	
Approximately 9 565		**TITLE:** ((((Genetic or gene*) near/2 associat* near/2 (studies or study or analys*))))	
Approximately 93 935		#2 OR #1	
Approximately 382 014		**TITLE:** ((((TB or Tuberculosis* or Antitubercul*))))	
Approximately 219 324		**TITLE:** ((((gene* or genetic*) near/5 (mutat* or variant*))))	
Approximately 1 388 831		**TITLE:** ((((SNP or Genotyp* or Phenotyp* or Allele* or Pharmacogenet* or Pharmacogenom* or Polymorph*))))	
Approximately 38 430		**TITLE:** (((single* near/2 nucleotid* near/2 polymorph*)))	
Approximately 45 745		**TITLE:** ((((genetic* or gene*) near/3 (suscept* or predisposit* or anticipat*))))	
Approximately 47 961		**TITLE:** (((aminosalicylic acid or diarylquinoline* or ethambutol* or ethionamide* or isoniazid* or prothionamide* or pyrazinamide* or thioacetazone* or capreomycin* or cycloserine* or enviomycin* or rifabutin* or rifampin* or viomycin*)))	
Approximately 49 386		**TITLE:** ((((Antitubercul* or tuberculos* or TB) Near/4 (agent* or drug* or antibiotic* or medicine* or medication* or treatment*))))	

**B)** Database: Medline			

# ▴	Searches		Results

1	antitubercular agents/ or aminosalicylic acid/ or diarylquinolines/ or ethambutol/ or ethionamide/ or isoniazid/ or prothionamide/ or pyrazinamide/ or thioacetazone/ or antibiotics, antitubercular/ or capreomycin/ or cycloserine/ or enviomycin/ or rifabutin/ or rifampin/ or viomycin/		73 943
2	((Antitubercul* or tuberculos* or TB) adj4 (agent* or drug* or antibiotic* or medicine* or medication* or treatment*)).tw.		29 293
3	(aminosalicylic acid or diarylquinoline* or ethambutol* or ethionamide* or isoniazid* or prothionamide* or pyrazinamide* or thioacetazone* or capreomycin* or cycloserine* or enviomycin* or rifabutin* or rifampin* or viomycin*).tw.		26 053
4	1 or 2 or 3		93 357
5	Polymorphism, Genetic/		103 705
6	genetic predisposition to disease/ or anticipation, genetic/		101 390
7	Pharmacogenetics/		9 595
8	Genetic Association Studies/		14 210
9	((Genetic or gene*) adj2 associat* adj2 (studies or study or analys*)).tw.		4 883
10	((genetic* or gene*) adj3 (suscept* or predisposit* or anticipat*)).tw.		40 247
11	Polymorphism, Single Nucleotide/		77 811
12	(single* adj2 nucleotid* adj2 polymorph*).tw.		46 260
13	(SNP or Genotyp* or Phenotyp* or Allele* or Pharmacogenet* or Pharmacogenom* or Polymorph*).tw.		774 469
14	((gene* or genetic*) adj5 (mutat* or variant*)).tw.		182 197
15	Genotype/ or Phenotype/ or Alleles/		381 555
16	or/5–15		1 035 512
17	exp Tuberculosis/		175 110
18	(TB or Tuberculosis*).tw.		153 175
19	Antitubercul*.tw.		11 635
20	or/17–19		213 138
21	4 and 16 and 20		2 846
22	animal/ not human/		4 159 388
23	21 not 22		2 730

**C)** Database: Embase			

# ▴	Searches		Results

1	antitubercular agents/ or aminosalicylic acid/ or diarylquinolines/ or ethambutol/ or ethionamide/ or isoniazid/ or prothionamide/ or pyrazinamide/ or thioacetazone/ or antibiotics, antitubercular/ or capreomycin/ or cycloserine/ or enviomycin/ or rifabutin/ or rifampin/ or viomycin/		151 901
2	((Antitubercul* or tuberculos* or TB) adj4 (agent* or drug* or antibiotic* or medicine* or medication* or treatment*)).tw.		40 664
3	(aminosalicylic acid or diarylquinoline* or ethambutol* or ethionamide* or isoniazid* or prothionamide* or pyrazinamide* or thioacetazone* or capreomycin* or cycloserine* or enviomycin* or rifabutin* or rifampin* or viomycin*).tw.		34 743
4	1 or 2 or 3		172 588
5	Polymorphism, Genetic/		102 257
6	genetic predisposition to disease/ or anticipation, genetic/		97 585
7	Pharmacogenetics/		17 431
8	Genetic Association Studies/		876
9	((Genetic or gene*) adj2 associat* adj2 (studies or study or analys*)).tw.		7 890
10	((genetic* or gene*) adj3 (suscept* or predisposit* or anticipat*)).tw.		61 544
11	Polymorphism, Single Nucleotide/		98 303
12	(single* adj2 nucleotid* adj2 polymorph*).tw.		75 841
13	(SNP or Genotyp* or Phenotyp* or Allele* or Pharmacogenet* or Pharmacogenom* or Polymorph*).tw.		1 171 894
14	((gene* or genetic*) adj5 (mutat* or variant*)).tw.		294 715
15	Genotype/ or Phenotype/ or Alleles/		777 386
16	or/5–15		1 548 879
17	exp Tuberculosis/		197 008
18	(TB or Tuberculosis*).tw.		187 590
19	Antitubercul*.tw.		16 330
20	or/17–19		253 048
21	4 and 16 and 20		5 380
22	animal/ not human/		1 357 016
23	21 not 22		5 360
24	limit 23 to em=188300-201608		4 778

**D)** Database: PubMed			

#1	Search (((Antitubercul* or tuberculos* or TB))) AND ((agent* or drug* or antibiotic* or medicine* or medication* or treatment*))		124 242
#2	Search ((aminosalicylic acid or diarylquinoline* or ethambutol* or ethionamide* or isoniazid* or prothionamide* or pyrazinamide* or thioacetazone* or capreomycin* or cycloserine* or enviomycin* or rifabutin* or rifampin* or viomycin*))		49 591
#3	Search (#1 or #2)		151 329
#4	Search ((((Genetic or gene*) near/2 near/2 ))) AND associat*) AND ((studies or study or analys*))		3 922
#5	Search (((genetic* or gene*))) AND ((suscept* or predisposit* or anticipat*))		235 548
#6	Search ((single*) AND nucleotid*) AND polymorph*		98 071
#7	Search ((SNP or Genotyp* or Phenotyp* or Allele* or Pharmacogenet* or Pharmacogenom* or Polymorph*))		997 538
#8	Search (((gene* or genetic*))) AND ((mutat* or variant*))		743 819
#9	Search (#4 or #5 or #6 or #7 or #8)		1 553 428
#10	Search (((((TB or Tuberculosis* or Antitubercul*)))))		251 923
#11	Search (#3 and #9 and #10)		7 671
#12	Search (“2015/08/01”[Date - Entrez] : “3000”[Date - Entrez])		658 085
#13	Search (#11 and #12)		379

**Table A.3:** Key characteristics of included studies

Author, year	Country	Study design	Follow-up time	Drugs and dosage	Selection criteria	Sample size *n*	Toxicity outcomes
An, 2012	China	App	6 months	Daily treatment with INH, RMP, PZA and EMB for 2 months, followed by 4 months treatment with INH and RMP, with drug dosages calculated according to body weight Body weight < 45 kg: RMP 300 mg, INH 200 mg, PZA 1000 mg Body weight of 45–55 kg: RMP 450 mg, INH 300 mg, PZA 1500 mg Body weight > 55 kg: RMP 600 mg, INH 400 mg, PZA 2000 mg	Inclusion criteria: Normal serum ALT, AST and bilirubin levels, no symptoms related to abnormal liver function (i.e., jaundice) before anti-tuberculosis drug treatment and close monitoring of changes in liver functions within 6 months of treatment Patients with and without hepatotoxicity during treatment Exclusion criteria: Malnutrition HIV type 1 infection Alcoholic liver disease or habitual drinking Hepatitis B or C infection, liver disease, systemic diseases and/or treatment with drugs other than the anti-tuberculosis drugs that can induce hepatotoxicity Severe TB or cardiac dysfunction that may cause liver dysfunction Transient increases in ALT	208	Hepatotoxocity
Azuma, 2013	Japan	RCT	6 months	All patients were treated with a 6-month regimen comprising INH, RMP, PZA and EMB/SM for the first 2 months, followed by RMP and INH for 4 months. All patients were started on the standard oral dose (~5 mg/kg body weight, once-daily). For pharmacogenetics-treatment patients, dosages were adjusted based on individual *NAT2* status within 3 days. Modified daily INH doses were respectively ~7.5, ~5 and ~2.5 mg/kg for rapid, intermediate and slow acetylators. Regarding the other drugs for the standard regimen, standard daily doses of RMP (10 mg/kg, maximum 600 mg/body), PZA (25 mg/kg, 1500 mg/body), EMB (15 mg/kg, 750 mg/body; 20 mg/kg, 1000 mg/body) and SM (15 mg/kg, 750 mg/body) were recommended with the following dose ranges allowed at the discretion of the physician in charge: RMP 8–12 mg/kg; PZA 20–30 mg/kg; EMB 15–20 mg/kg; SM 12–18 mg/kg	The eligible population was identified from a series of patients newly diagnosed with PTB (male and female aged 20–75 years), requiring the 6-month 4-drug standard treatment for the first time Exclusion criteria: abnormal test results for liver and kidney function (serum AST > 45 IU/l, ALT > 50 IU/l, ALP > 444 IU/l, total bilirubin > 1.6 mg/dl and creatinine > 1.4 mg/dl before the study treatments commenced, long-term use of steroids and/or immunosuppressants, inadequate clinical conditions such as hyperglycaemia, diabetes mellitus, acute life-threatening chronic progressive disease, pregnancy or lactation and alcoholism. Patients not expected to complete the study protocol for social reasons were not recruited	172	INH-DILI Peripheral neuropathy
Bose, 2011	India	Prospective cohort	Patients were followed up to 8 weeks after they had started with anti-tuberculosis treatment	All patients received anti-tuberculosis treatment (RMP, INH, EMB and PZA) according to body weight. All four drugs were given for 2 months. PZA and EMB were discontinued, while INH and RMP were continued for another 4 months RMP doses as follows: ⩽35 kg: 300 mg/day 36–50 kg: 450 mg/day >50 kg: 600 mg/day INH doses as follows: ⩽35 kg: 200 mg/day >35 kg: 400 mg/day PZA doses as follows: ⩽50 kg: 1.0 g/day >50 kg: 1.5 g/day EMB 20 mg/kg/day	Newly diagnosed patients of PTB. The baseline LFT of the patients was normal when they were started on anti-tuberculosis treatment Exclusion criteria: no history of habitual alcoholism, chronic liver diseases or steatosis	218	ADIH ADIH outcome
Çetintaş, 2008	Turkey	Prospective cohort	NR	Patients received INH 5 mg/kg (maximum 300 mg/day), RMP 10 mg/kg (maximum 600 mg/day), PZA 25 mg/kg (maximum 2000 mg/day), EMB 15–25 mg/kg (maximum 1500 mg/day)	Patients diagnosed with TB. Only patients with serum levels before initiation of treatment within the following ranges were included in the study: ALT 0–40 U/l, AST 5–45 U/l and total bilirubin 0.2–1.6 mg/dl Exclusion criteria: patients with positive serum hepatitis B surface antigen, hepatitis C antibody or hepatitis A immunoglobulin M antibody, and patients with alcoholic liver disease or any hepatic or systemic disease that could cause liver function disorder	100	DIH
Chamorro, 2013	Argentina	Prospective cohort	NR	The patients began a standard anti-tuberculosis treatment protocol for the first 2 months (INH 5 mg/kg/days, maximum 300 mg/day, RMP 10 mg/kg/day maximum 600 mg/day, PZA 20 mg/kg/day, EMB 20 mg/kg/day), followed by INH and RMP for ⩾4 months, depending on the disease severity or the presence ofextra-pulmonary foci	Inclusion criteria: TB patients aged >18 years with stable haemodynamic levels, normal renal function and negative for pregnancy Exclusion criteria: presence of diseases that directly affected the liver (acute hepatitis A, active hepatitis B and C; cirrhosis; encephalopathy and cancer), INH allergy, HIV, autoimmunity, concomitant hepatotoxic medications, a history of TB treatment failures, refusal of blood extraction and refusal to sign the written informed consent	175	ATDH
Chang, 2012	Taiwan	Prospective cohort	NR	First-line anti-tuberculosis medications	Patients diagnosed with TB and treated with first-line anti-tuberculosis medications Exclusion criteria: pregnancy, abnormal liver function and history of positive viral hepatitis before starting treatment with first-line anti-tuberculosis drugs	98	ATDH
Cho, 2007	Korea	Prospective cohort	Serum AST, ALT and total bilirubin levels were then monitored monthly until the end of treatment	All patients received oral INH (300 mg), RMP (600 mg), PZA (20 mg/kg body weight) and EMB (800 mg) daily for the first 2 months. PZA was then discontinued, while INH, RMP and EMB were continued for another 4 months	Adult patients newly diagnosed with active TB, with evident lesion of TB using simple X-ray or computed tomography or positive results on sputum smear or culture for detection of mycobacteria Exclusion criteria: 1) abnormal serum ALT, AST, or bilirubin levels or symptoms related to abnormal liver function such as jaundice before anti-tuberculosis treatment; 2) alcoholic liver disease or habitual alcohol drinking; 3) any other hepatic or systemic diseases that may cause liver dysfunction	132	ATDH
Costa, 2012	Brazil	Prospective cohort	NR	All patients were treated with the first-line anti-tuberculosis drug regimen INH (300 mg/kg/day), RMP (300 mg/kg/day) and PZA (1500 mg/kg/day) for the first 2 months, followed by INH and RMP for a further 4 months	Male or female subjects aged ⩾18 years, with no previously described renal, allergic or hepatic diseases and not pregnant	129	ADRs
Dhoro, 2013	Zimbabwe	Case-control	NR	NR	TB patients	Not clear—189 received INH	Peripheral neuropathy
Feng, 2014	China	Case-control	6 months	Treatment with anti-tuberculosis drug regimens at the usual dosage—300 mg/day INH, 450 mg/day RMP and 1500 mg/day PZA	Selection of cases: cases selected based on liver functions, i.e., all indices of liver function were normal before anti-tuberculosis chemotherapy, and became abnormal indicating hepatic injury after 6 months of chemotherapy. Cases were patients who showed ATDH based on increased serum transaminase values that were three-fold higher than the ULN (40 IU/l ALT) and symptoms compatible with hepatitis Selection of controls: controls underwent the same anti-tuberculosis chemotherapy with selected cases and were not tested for abnormality in liver functions after 6 months of the chemotherapy. The controls selected matched the criteria compared to the cases: 1) sex; 2) age discrepancy of <5 years; 3) living in the same regions; and 4) treatment with anti-tuberculosis drug regimens at the usual dosage, including 300 mg/day INH, 450 mg/day RMP and 1500 mg/day PZA	346	ATDH
Fredj, 2016	Tunisia	Prospective cohort	Serum AST, ALT and ALP were monitored monthly until the end of the treatment	The anti-tuberculosis treatment was based on the association of INH (5 mg/kg/day), RMP (10 mg/kg/day), PZA (25 mg/kg/day) and EMB (15 mg/kg/day) for the first 2 months, followed by INH and RMP for 4–7 additional months, depending on TB clinical presentation	Patients diagnosed with PTB and EPTB	71	INH-induced-hepatotoxicity
Gupta, 2013 (GI: GUPTA)	India	Prospective cohort	The patients were monitored for ALT, AST, andtotal bilirubin levels weekly for 1 month and then monthly until the completion of treatment	Initially patients received a combination regimen including INH 5 mg/kg (max 300 mg daily), RMP 10 mg/kg (max 600 mg daily), PZA 25 mg/kg (max 1500 mg daily) and EMB 15–25 mg/kg (max 2000 mg daily) for a period of 2 months, followed by an additional 4 months with INH and RMP	Inclusion criteria: 1) age > 18 years; 2) smear and/or culture positive for mycobacteria in clinical samples; and 3) normal ALT, AST and total bilirubin levels Exclusion criteria: 1) patients presenting clinically and laboratory-confirmed chronic liver disease such as jaundice; 2) acute and chronic hepatitis B and/or C or HIV; 3) alcoholic liver diseases; 4) a rise of two times the ULN of ALT, AST and total bilirubin levels; 5) medication with anti-tuberculosis drugs before start of treatment and/or other potentially hepatotoxic drugs; and 6) refusal to provide blood sample or signed informed consent form	215	ATDH
Higuchi, 2007 (GI: HIGUCHI)	Japan	Prospective cohort	NR	Treated with a INH (400 mg/d) and RMP (450 mg/d) containing regimen for 6 or 9 months	Patients with new onset of PTB treated with a INH (400 mg/d) and RMP (450 mg/d) containing regimen for 6 or 9 months Exclusion criteria: patients with liver cirrhosis, chronic and acute hepatitis, alcoholic liver disease and other chronic liver diseases	100	ATDH Skin rash Eosinophilia
Ho, 2013	Taiwan	Prospective cohort	180 days	All patients received oral INH 300 mg, RMP 600 mg (or 450 mg if body weight was <50 kg), PZA 25 mg/kg of body weight (max daily dose 2000 mg) and EMB 15 mg/kg of body weight daily (max daily dose 1600 mg) for the first 2 months. PZA was then discontinued, whereas INH, RMP and EMB were continued for another 4 months	The inclusion criteria included a sputum smear with AFB or culture positive for M. tuberculosis, undergoing anti-tuberculosis treatment, including INH or starting treatment during the recruitment period, hepatitis serology recorded in the medical chart with signed written consent Patients were excluded if they were infected with hepatitis B virus, hepatitis C virus or HIV, had any other hepatic disease, or did not have at least two visits documented in the medical records	348	ATDH
Huang, 2003 (GI: HUANG)	Taiwan	Prospective cohort	Serum ALT, AST and total bilirubin levels were monitored monthly until the end of treatment or checked whenever patients had symptoms of suspected hepatitis	Their standard daily anti-tuberculosis regimen for the first 2 months included INH (300 mg), RMP (600 mg or 450 mg if body weight <50 kg), PZA (20 mg/kg body weight) and EMB (25 mg/kg body weight). PZA was then discontinued, whereas INH, RMP, and EMB (15 mg/kg body weight) were continued for another 4 months	Patients with incident PTB or EPTB Exclusion criteria: 1) abnormal serum ALT, AST or bilirubin before anti-tuberculosis treatment; and 2) refusal of blood sampling or informed written consent	318	ADIH
Jung, 2015	Korea	Prospective cohort	4 weeks	Standard 4-drug treatment for 6 months: INH (5 mg/kg, usually 300 mg), RMP (450 mg for <50 kg or 600 mg for ⩾50 kg of body weight), EMB (15 mg/kg) and PZA (20–30 mg/kg), daily for 2 months, followed by INH and RMP with or without EMB for 4 months. Doses were adjusted when applying model-based treatment	Eligible participants were patients newly diagnosed with active TB, who underwent standard 4-drug treatment for 6 months Exclusion criteria: patients with abnormal hepatic function on laboratory testing (increased serum AST, ALT or total bilirubin) before anti-tuberculosis treatment and underlying liver disease or systemic illness such as congestive heart failure, acute life-threatening disease, or alcoholism or disease that was resistant to INH at the start of treatment	206	ATDH
Khalili, 2011	Iran	Case-control	2 months	Treated daily with INH (300 mg), RMP (600 mg), PZA (20 mg/kg), EMB (15 mg/kg) for the first 2 months, followed by INH and RMP daily for 4 additional months	Inclusion criteria: newly diagnosed patients (>18 years) with active PTB, who had been planned to be treated daily with anti-tuberculosis treatment (see drugs and dosage) Exclusion criteria: other possibilities of hepatitis, including patients with HIV infection, hepatic insufficiency (ALT or AST > 2× ULN) or clinically signs and symptoms of liver diseases such as jaundice and ascites, chronic hepatitis (B or C) and renal insufficiency (creatinine clearance < 50 ml/min based on Cockcroft-Gault equation) and history or current use of herbal, supplemental and hepatotoxic non-tuberculosis drugs	100	Hepatotoxicity
Kim, 2009 (GI: KIM)	Korea	Case-control	Assessments performed 2 weeks after onset of treatment and bi-monthly thereafter	All patients with PTB were treated daily with a combination regimen comprising INH (300–400 mg daily), RMP (450–600 mg daily), EMB (600–800 mg daily) and PZA (1000–1500 mg daily) for 2 months and then without PZA for ⩾4 following months. Doses of each drug were adjusted based on body weight of the patient	Newly diagnosed and treated patients with PTB. Exclusion criteria: patients with active or chronic hepatitis, including alcoholic hepatitis, fatty liver disease, liver cirrhosis, carriers of the hepatitis B or C virus, heavy alcohol intake, decreased renal function and severe cardiac diseases requiring several medications	226	ATDH
Kim, 2011 (GI: KIM)	Korea	Case-control	NR	The treatment comprised an initial phase of 2 months and a subsequent continuation phase of ⩾4 months. During the initial phase, 4 drugs were administered, including INH (300–400 mg daily), RMP (450–600 mg daily), EMB (600–800 mg daily) and PZA (1000–1500 mg daily). Doses of each drug were adjusted based on the body weight of the patient. In the following continuation phase, only PZA was discontinued, while the other three drugs were continued	Patients newly diagnosed with PTB and/or TB pleuritis and treated with first-line anti-tuberculosis medications such as INH, RMP, EMB and PZA Exclusion criteria: 1) patients with skin diseases before treatment, 2) chronic renal failure and chronic liver diseases affecting drug metabolism, 3) chronic alcoholism, 4) other chronic medical conditions requiring medication, and 5) non-adherence to treatment	221	ATD-induced MPE
Lee, 2010	Taiwan	Prospective cohort	NR	All patients received oral INH 300 mg, RMP 600 mg (or 450 mg, if body weight was <50 kg), PZA 200 mg/kg of body weight and EMB 800 mg daily for the first 2 months. PZA was then discontinued, while INH, RMP and EMB were continued for another 4 months	Inclusion criteria: adult patients newly diagnosed with active TB, having evident lesions of TB using simple X-ray, computed tomography, sputum smears and cultures positive for mycobacteria Exclusion criteria: 1) positive serum hepatitis B virus surface antigen, antibody to hepatitis C virus; 2) alcoholic liver disease or habitual alcohol drinking; 3) any other hepatic or systemic diseases that may cause liver dysfunction; 4) abnormal serum ALT, AST or bilirubin levels before anti-tuberculosis treatment	140	ATDH
Leiro-Fernandez, 2011	Spain	Case-control	Routine follow-up of clinical assessments every 2 weeks during the first month and monthly thereafter untilcompletion of treatment	Treatment with regimens that included INH, RMP and PZA at the usual dosages (INH 5 mg/kg/day to maximum 300 mg/day, RMP10 mg/kg/day to maximum 600 mg/day and PZA 25–30 mg/kg/day to maximum 2500 mg/day	Inclusion criteria: 1) age 15–75 years; 2) microbiological demonstration of active TB; 3) treatment with regimens that included INH, RMP and PZA at the usual dosages (INH 5 mg/kg/day to max 300 mg/day, RMP 10 mg/kg/day to max 600 mg/day and PZA 25–30 mg/kg/day to max 2500 mg/day); and 4) adequate treatment adherence Exclusion criteria: 1) increased baseline serum transaminases (AST and/or ALT; normal values ⩽ 40 IU/l); 2) positive serological testing for HIV, hepatitis B virus or hepatitis C virus; 3) regular alcohol intake or concomitant use of hepatotoxic drugs; 4) history of chronic liver disease; 5) pregnancy; or 6) no or poor adherence to treatment	117	ATDH
Lv, 2012	China	Case-control	6–9 months	All primary/retreatment patients took INH (600 mg), RMP (600 mg or 450 mg if body weight was <50 kg), PZA (2000 mg) and EMB (1250 mg) every other day in the first 2 months and then INH and RMP were continued for another 4/6 months. The retreatment patients received SM (750 mg) every other day in the first 2 months and continued receiving EMB for another 6 months	Inclusion criteria: sputum smear-positive PTB patients who received standard short-course chemotherapy recommended by the WHO Exclusion criteria: 1) positive serum hepatitis B virus surface antigen or other liver disease, 2) potentially hepatotoxic medications that would confound the picture, 3) abnormal serum ALT, AST or total bilirubin levels before anti-tuberculosis treatment	445	ATDH
Mahmoud, 2012	Tunisia	Prospective cohort	3 months	Treated with INH and RMP containing regimen	Inclusion criteria: adult patients (>18 years) newly diagnosed with TB treated with INH- and RMP-containing regimen, LFTs before initiation of treatment showed completely normal findings on serum ALT, AST, total bilirubin, ALP and gGTP Exclusion criteria: 1) patients receiving other potentially hepatotoxic drugs in addition to anti-tuberculosis agents; 2) positive serum hepatitis B surface antigen or hepatitis C antibody; 3) alcoholic liver disease; 4) any other hepatic or systemic diseases that may cause liver dysfunction	66	INH hepatotoxicity
Ng, 2014	Cases recruited in the UK; controls were samples from elsewhere	Case-control	NR	All had been prescribed INH with all but one also taking additional anti-tuberculosis drugs. Patients were prescribed 300 mg INH/day in line with UK/WHO guidelines	Cases: patients diagnosed with DILI either in the past or at the time of sample collection. Onlycases assessed as having DILI that was highly probably, probably or possibly relating to anti-tuberculosis drug exposure were enrolled in the study. Other possible causes of liver toxicity, particularly hepatitis A, B, C and CMV infection were excluded. No patients included were HIV-positive or had pre-existing liver disease. None had been treated for TB under directly observed treatment Controls: 52 DNA samples from individuals of European ancestry; inclusion/exclusion criteria not reported	127	DILI
Ohno, 2000	Japan	Prospective cohort	3 months	Initial chemotherapy always included INH (400 mg/day; 8.2 ± 2.0 mg/kg/day) and RMP (450 mg/day; 9.2 ± 2.2 mg/kg/day); the third drug used was EMB or SM.	Inclusion criteria: patients had no history of alcohol abuse and were negative for hepatitis B surface antigen and hepatitis C antibody. At the beginning of treatment, LFTs showed completely normal findings on serum AST, ALT, bilirubin, ALP and gGTP Patients receiving other potentially hepatopathic drugs in addition to anti-tuberculosis agents were excluded from the study	77	INH + RMP-induced hepatotoxicity
Possuelo, 2008 (GI: POSSUELO)	Brazil	Prospective cohort	NR	Treatment daily with INH, RMP and PZA for the first 2 months followed by INH and RMP daily for 4 additional months. Drug dosages used were calculated according to patient's weight (weight <45 kg: RMP 300 mg, INH 200 mg, PZA 1000 mg; 45–55 kg: RMP 450 mg, INH 300 mg, PZA 1500 mg; >55 kg: RMP 600 mg, INH 400 mg, PZA 2000 mg)	Inclusion criteria: adult patients (>18 years) newly diagnosed with active TB, who had been treated daily with anti-tuberculosis treatment (see drugs and dosage) Exclusion criteria: patients presenting clinically and laboratory-confirmed liver chronic disease, patients using anti-tuberculosis drugs before enrolment in the study, patients presenting results of LFTs before the beginning of treatment higher that were 2× ULN and refusal to participate of the study	254	ATDH Gastrointestinal ADRs
Rana, 2014 (GI: RANA)	India	Prospective cohort	Patients were monitored every month until the end of treatment or whenever the patients had symptoms or signs of hepatotoxicity	Daily anti-tuberculosis treatment for the first 2 months included INH (300 mg), RMP (600 or 450 mg for body weight/50 kg), PZA (20 mg/kg body weight) and EMB (25 mg/kg body weight). After 2 months, EMB and PZA were discontinued, whereas INH and RMP were continued for an additional 4 months	Inclusion criteria: patients with pulmonary and EPTB Exclusion criteria: patients with history of alcohol abuse and/or any other liver disease; patients who had received other potentially hepatotoxic drugs in addition to anti-tuberculosis drugs; patients who had abnormal serum ALT, AST or bilirubin before starting anti-tuberculosis treatment; patientswho developed viral hepatitis during anti-tuberculosis treatment; patients with renal failure or cancer; and patients who refused blood sampling or providing informed written consent	300	Hepatotoxicity
Santos, 2013 (GI: SANTOS)	Brazil	Prospective cohort	NR	Treatment with INH, RMP and PZA for the first 2 months, followed by INH and RMP daily for 4 months	Inclusion criteria: patients diagnosed with TB and treated with anti-tuberculosis treatment (see drugs and dosage) Exclusion criteria: patients aged <18 years, those with mental disabilities, chronic liver disease confirmed by clinical and laboratory data, users of anti-tuberculosis drugs before enrolment in the study, and those with liver function results > 2× ULN before beginning treatment	270	ATDH
Shimizu, 2006	Japan	Prospective cohort	3 months	Treatment with INH (300–400 mg) and RMP (300–450 mg)	Individuals with PTB without abnormal serum levels of ALT, AST, direct or total bilirubin, or serum hepatitis B virus surface antigen or antibody to hepatitis C virus at the beginning of the study	42	Hepatotoxicity
Singla, 2014	India	Prospective cohort	NR	NR	Inclusion criteria: newly diagnosed patients with TB Exclusion criteria: history of heavy use of alcohol or chronic liver diseases or liver cirrhosis;patients who were settled in the area for a minimum of three generations; people infected with HIV	408	ATDH
Sotsuka, 2011	Japan	Prospective cohort	3 months	INH, RMP and PZA, plus EMB or SM during the first 2 months, followed by administration of INH and RMP plus EMB or SM during the final 4 months	Inclusion criteria: in-patients with active PTB who were treated with the standard Japanese chemotherapy regimen, followed up for >3 months after treatment, and who consented to this study	144	Hepatotoxicity
Teixeira, 2011	Brazil	Case-control	NR	Anti-tuberculosis drug regimens that include INH at the usual dosage (400 mg/day)	Inclusion criteria: 1) age > 18 years, 2) diagnosis of active TB, 3) treatment with anti-tuberculosis drug regimens that include INH at the usual dosage (400 mg/day) and 4) normal baseline serum transaminases (ALT and AST) before treatment Exclusion criteria: 1) positive serological test for the HIV, hepatitis B virus or hepatitis C virus, 2) alcohol abuse, 3) history of chronic liver disease and 4) pregnancy	167	Hepatitis
Vuilleumier, 2006	Switzerland	Prospective cohort	NR	INH 300 mg daily and vitamin B6 40 mg per day for a period of 6 months	Inclusion criteria: patients having LTBI as defined by the American Thoracic Society with normal plasma AST and ALT levels before the beginning of INH monotherapy Exclusion criteria: 1) a history of alcohol consumption, 2) positive serology for the HAV, HBV or HCV, 3) poor chemotherapy compliance (negative urine INH 3 times during the follow-up), loss during the follow-up, 4) patients had to receive supplementary anti-tuberculosis agents or other potentially hepatotoxic drugs	89	INH-induced hepatotoxicity
Wang, 2015 (GI: NTUH)	Taiwan	Prospective cohort	6 months	Daily INH, RMP, EMB and PZA in the first 2 months, and daily INH and RMP for the next 4 months The daily dosage of each drug was calculated by weight	Adult Taiwanese (>16 years) patients with culture-confirmed PTB Subjects were excluded if they were pregnant, had a life expectancy < 6 months, had abnormal baseline LFT or were resistant to INH, RMP or both	355 in the derivation cohort, 182 in the validation cohort	Hepatotoxicity during anti-tuberculosis treatment
Xiang, 2014	China	Prospective cohort	2 months	All patients were prescribed INH (600 mg), RMP (600 mg or 450 mg if the body weight was <50 kg), PZA (2000 mg) and EMB (1250 mg) every other day in the first 2 months. After 2 months, INH and RMP were continued for a further 4–6 months. Retreatment patients in addition received SM (750 mg) every other day in the first 2 months and continued receiving EMB for another 6 months	Inclusion criteria: newly diagnosed PTB patients belonging to the Uyghur ethnic group, who were receiving standard short-course chemotherapy recommended by the WHO, who attended for a 2-month assessment, and any patients attending clinic with suspected liver disease after the start of treatment, before the 2-month visit Exclusion criteria: patients who had signs of abnormal liver function when they started treatment (jaundice or elevated ALT, AST or bilirubin levels) or disease associated with liver dysfunction	2244	ATLI
Yamada, 2009 (GI: YAMADA)	Canada	Prospective cohort	9 months	INH 300 mg	Subjects who were receiving treatment with INH 300 mg daily self-administered for LTBI.Subjects were included if they were aged ⩾19 years; not concurrently receiving other anti-tuberculosis drugs; non-reactive to hepatitis B surface antigen and antibody to hepatitis C virus; absence of any liver or metabolic diseases; without a HIV-positive test result; not consuming ⩾7 alcoholic beverages per day and undergoing sufficiently frequentAST tests to detect hepatotoxicity	170	Hepatotoxicity
Yimer, 2011	Ethiopia	Prospective cohort	followed up for development of DILI for up to 56 weeks	All study participants received RMP based short-course chemotherapy for TB following the national TB treatment guideline. ART was then initiated	Newly diagnosed ART and anti-tuberculosis treatment-naïve adult TB and HIV co-infected patients. The eligibility criteria were age >18 years, CD4 count, 200 cells/Ul, not pregnant and not on other known hepatotoxic drugs concurrently (except cotrimoxazole, 960 mg per day, which was given to all participants before enrolment and during the follow-up period according to the treatment guideline)	353	Anti-tubercular and antiretroviral DILI
Yuliwulandari, 2016	Indonesia	Case-control	NR	NR (anti-tuberculosis treatment)	Patients included in the study were male and female aged 15–70 years, suffering from TB according to the WHO standards, and were under monitored treatment with anti-tuberculosis drugs Exclusion criteria: 1) history of liver disease such as: hepatitis A B or C; hepatoma; liver cirrhosis or cholelithiasis positive; 2) abnormal levels of any LFT (ALT, AST or total bilirubin) before anti-tuberculosis treatment	241	ATLI
Zaverucha-do-Valle, 2014	Brazil	Retrospective cohort	Follow-up at days 15 and 30 and at least one visit monthly until the end of treatment	600 mg/day of RMP, 400 mg/day of INH and 2 g/day of PZA for all patients with corporal weight >45 kg or adjusted for corporal weight <45 kg. After 2 months of treatment, PZA was discontinued	Inclusion criteria: signed written consent, sputum smear with AFB or culture positive for M. tuberculosis; ongoing TB treatment and laboratory tests of liver function Exclusion criteria: age <18 years, pregnancy and no >1 visit registered	131	ATDH

INH =isoniazid; RMP =rifampicin; PZA =pyrazinamide; EMB =ethambutol; ALT =alanine aminotransferase; AST =aspartate aminotransferase; RCT =randomised controlled trial; HIV =human immunodeficiency virus; SM =streptomycin; PTB =pulmonary tuberculosis; IU =international unit; ALP =alkaline phosphatase; DILI =drug-induced liver injury; LFT =liver function test; DIH =drug-induced hepatotoxicity; NR =not reported; ATDH =anti-tuberculosis drug-induced hepatotoxicity; GI =group identifier; ADR =adverse drug reaction; ULN =upper limit of normal; EPTB =extra-pulmonary TB; ADIH =anti-tuberculosis drug-induced hepatitis; ATD =anti-tuberculosis drug; MPE =maculopapular eruption; WHO = World Health Organization; gGTP = gamma-glutamyltranspeptidase; CMV = cytomegalovirus ; LTBI = latent tuberculous infection; ART =antiretroviral therapy; ATLI = anti-tuberculosis drug-induced liver injury.

**Table A.3:** (*continued*)

Author, year	Country	Study design	Follow-up time	Drugs and dosage	Selection criteria	Sample size *n*	Toxicity outcomes
An, 2012	China	App	6 months	Daily treatment with INH, RMP, PZA and EMB for 2 months, followed by 4 months treatment with INH and RMP, with drug dosages calculated according to body weight Body weight < 45 kg: RMP 300 mg, INH 200 mg, PZA 1000 mg Body weight of 45–55 kg: RMP 450 mg, INH 300 mg, PZA 1500 mg Body weight > 55 kg: RMP 600 mg, INH 400 mg, PZA 2000 mg	Inclusion criteria: Normal serum ALT, AST and bilirubin levels, no symptoms related to abnormal liver function (i.e., jaundice) before anti-tuberculosis drug treatment and close monitoring of changes in liver functions within 6 months of treatment Patients with and without hepatotoxicity during treatment Exclusion criteria: Malnutrition HIV type 1 infection Alcoholic liver disease or habitual drinking Hepatitis B or C infection, liver disease, systemic diseases and/or treatment with drugs other than the anti-tuberculosis drugs that can induce hepatotoxicity Severe TB or cardiac dysfunction that may cause liver dysfunction Transient increases in ALT	208	Hepatotoxocity
Azuma, 2013	Japan	RCT	6 months	All patients were treated with a 6-month regimen comprising INH, RMP, PZA and EMB/SM for the first 2 months, followed by RMP and INH for 4 months. All patients were started on the standard oral dose (~5 mg/kg body weight, once-daily). For pharmacogenetics-treatment patients, dosages were adjusted based on individual *NAT2* status within 3 days. Modified daily INH doses were respectively ~7.5, ~5 and ~2.5 mg/kg for rapid, intermediate and slow acetylators. Regarding the other drugs for the standard regimen, standard daily doses of RMP (10 mg/kg, maximum 600 mg/body), PZA (25 mg/kg, 1500 mg/body), EMB (15 mg/kg, 750 mg/body; 20 mg/kg, 1000 mg/body) and SM (15 mg/kg, 750 mg/body) were recommended with the following dose ranges allowed at the discretion of the physician in charge: RMP 8–12 mg/kg; PZA 20–30 mg/kg; EMB 15–20 mg/kg; SM 12–18 mg/kg	The eligible population was identified from a series of patients newly diagnosed with PTB (male and female aged 20–75 years), requiring the 6-month 4-drug standard treatment for the first time Exclusion criteria: abnormal test results for liver and kidney function (serum AST > 45 IU/l, ALT > 50 IU/l, ALP > 444 IU/l, total bilirubin > 1.6 mg/dl and creatinine > 1.4 mg/dl before the study treatments commenced, long-term use of steroids and/or immunosuppressants, inadequate clinical conditions such as hyperglycaemia, diabetes mellitus, acute life-threatening chronic progressive disease, pregnancy or lactation and alcoholism. Patients not expected to complete the study protocol for social reasons were not recruited	172	INH-DILI Peripheral neuropathy
Bose, 2011	India	Prospective cohort	Patients were followed up to 8 weeks after they had started with anti-tuberculosis treatment	All patients received anti-tuberculosis treatment (RMP, INH, EMB and PZA) according to body weight. All four drugs were given for 2 months. PZA and EMB were discontinued, while INH and RMP were continued for another 4 months RMP doses as follows: ⩽35 kg: 300 mg/day 36–50 kg: 450 mg/day >50 kg: 600 mg/day INH doses as follows: ⩽35 kg: 200 mg/day >35 kg: 400 mg/day PZA doses as follows: ⩽50 kg: 1.0 g/day >50 kg: 1.5 g/day EMB 20 mg/kg/day	Newly diagnosed patients of PTB. The baseline LFT of the patients was normal when they were started on anti-tuberculosis treatment Exclusion criteria: no history of habitual alcoholism, chronic liver diseases or steatosis	218	ADIH ADIH outcome
Çetintaş, 2008	Turkey	Prospective cohort	NR	Patients received INH 5 mg/kg (maximum 300 mg/day), RMP 10 mg/kg (maximum 600 mg/day), PZA 25 mg/kg (maximum 2000 mg/day), EMB 15–25 mg/kg (maximum 1500 mg/day)	Patients diagnosed with TB. Only patients with serum levels before initiation of treatment within the following ranges were included in the study: ALT 0–40 U/l, AST 5–45 U/l and total bilirubin 0.2–1.6 mg/dl Exclusion criteria: patients with positive serum hepatitis B surface antigen, hepatitis C antibody or hepatitis A immunoglobulin M antibody, and patients with alcoholic liver disease or any hepatic or systemic disease that could cause liver function disorder	100	DIH
Chamorro, 2013	Argentina	Prospective cohort	NR	The patients began a standard anti-tuberculosis treatment protocol for the first 2 months (INH 5 mg/kg/days, maximum 300 mg/day, RMP 10 mg/kg/day maximum 600 mg/day, PZA 20 mg/kg/day, EMB 20 mg/kg/day), followed by INH and RMP for ⩾4 months, depending on the disease severity or the presence ofextra-pulmonary foci	Inclusion criteria: TB patients aged >18 years with stable haemodynamic levels, normal renal function and negative for pregnancy Exclusion criteria: presence of diseases that directly affected the liver (acute hepatitis A, active hepatitis B and C; cirrhosis; encephalopathy and cancer), INH allergy, HIV, autoimmunity, concomitant hepatotoxic medications, a history of TB treatment failures, refusal of blood extraction and refusal to sign the written informed consent	175	ATDH
Chang, 2012	Taiwan	Prospective cohort	NR	First-line anti-tuberculosis medications	Patients diagnosed with TB and treated with first-line anti-tuberculosis medications Exclusion criteria: pregnancy, abnormal liver function and history of positive viral hepatitis before starting treatment with first-line anti-tuberculosis drugs	98	ATDH
Cho, 2007	Korea	Prospective cohort	Serum AST, ALT and total bilirubin levels were then monitored monthly until the end of treatment	All patients received oral INH (300 mg), RMP (600 mg), PZA (20 mg/kg body weight) and EMB (800 mg) daily for the first 2 months. PZA was then discontinued, while INH, RMP and EMB were continued for another 4 months	Adult patients newly diagnosed with active TB, with evident lesion of TB using simple X-ray or computed tomography or positive results on sputum smear or culture for detection of mycobacteria Exclusion criteria: 1) abnormal serum ALT, AST, or bilirubin levels or symptoms related to abnormal liver function such as jaundice before anti-tuberculosis treatment; 2) alcoholic liver disease or habitual alcohol drinking; 3) any other hepatic or systemic diseases that may cause liver dysfunction	132	ATDH
Costa, 2012	Brazil	Prospective cohort	NR	All patients were treated with the first-line anti-tuberculosis drug regimen INH (300 mg/kg/day), RMP (300 mg/kg/day) and PZA (1500 mg/kg/day) for the first 2 months, followed by INH and RMP for a further 4 months	Male or female subjects aged ⩾18 years, with no previously described renal, allergic or hepatic diseases and not pregnant	129	ADRs
Dhoro, 2013	Zimbabwe	Case-control	NR	NR	TB patients	Not clear—189 received INH	Peripheral neuropathy
Feng, 2014	China	Case-control	6 months	Treatment with anti-tuberculosis drug regimens at the usual dosage—300 mg/day INH, 450 mg/day RMP and 1500 mg/day PZA	Selection of cases: cases selected based on liver functions, i.e., all indices of liver function were normal before anti-tuberculosis chemotherapy, and became abnormal indicating hepatic injury after 6 months of chemotherapy. Cases were patients who showed ATDH based on increased serum transaminase values that were three-fold higher than the ULN (40 IU/l ALT) and symptoms compatible with hepatitis Selection of controls: controls underwent the same anti-tuberculosis chemotherapy with selected cases and were not tested for abnormality in liver functions after 6 months of the chemotherapy. The controls selected matched the criteria compared to the cases: 1) sex; 2) age discrepancy of <5 years; 3) living in the same regions; and 4) treatment with anti-tuberculosis drug regimens at the usual dosage, including 300 mg/day INH, 450 mg/day RMP and 1500 mg/day PZA	346	ATDH
Fredj, 2016	Tunisia	Prospective cohort	Serum AST, ALT and ALP were monitored monthly until the end of the treatment	The anti-tuberculosis treatment was based on the association of INH (5 mg/kg/day), RMP (10 mg/kg/day), PZA (25 mg/kg/day) and EMB (15 mg/kg/day) for the first 2 months, followed by INH and RMP for 4–7 additional months, depending on TB clinical presentation	Patients diagnosed with PTB and EPTB	71	INH-induced-hepatotoxicity
Gupta, 2013 (GI: GUPTA)	India	Prospective cohort	The patients were monitored for ALT, AST, andtotal bilirubin levels weekly for 1 month and then monthly until the completion of treatment	Initially patients received a combination regimen including INH 5 mg/kg (max 300 mg daily), RMP 10 mg/kg (max 600 mg daily), PZA 25 mg/kg (max 1500 mg daily) and EMB 15–25 mg/kg (max 2000 mg daily) for a period of 2 months, followed by an additional 4 months with INH and RMP	Inclusion criteria: 1) age > 18 years; 2) smear and/or culture positive for mycobacteria in clinical samples; and 3) normal ALT, AST and total bilirubin levels Exclusion criteria: 1) patients presenting clinically and laboratory-confirmed chronic liver disease such as jaundice; 2) acute and chronic hepatitis B and/or C or HIV; 3) alcoholic liver diseases; 4) a rise of two times the ULN of ALT, AST and total bilirubin levels; 5) medication with anti-tuberculosis drugs before start of treatment and/or other potentially hepatotoxic drugs; and 6) refusal to provide blood sample or signed informed consent form	215	ATDH
Higuchi, 2007 (GI: HIGUCHI)	Japan	Prospective cohort	NR	Treated with a INH (400 mg/d) and RMP (450 mg/d) containing regimen for 6 or 9 months	Patients with new onset of PTB treated with a INH (400 mg/d) and RMP (450 mg/d) containing regimen for 6 or 9 months Exclusion criteria: patients with liver cirrhosis, chronic and acute hepatitis, alcoholic liver disease and other chronic liver diseases	100	ATDH Skin rash Eosinophilia
Ho, 2013	Taiwan	Prospective cohort	180 days	All patients received oral INH 300 mg, RMP 600 mg (or 450 mg if body weight was <50 kg), PZA 25 mg/kg of body weight (max daily dose 2000 mg) and EMB 15 mg/kg of body weight daily (max daily dose 1600 mg) for the first 2 months. PZA was then discontinued, whereas INH, RMP and EMB were continued for another 4 months	The inclusion criteria included a sputum smear with AFB or culture positive for M. tuberculosis, undergoing anti-tuberculosis treatment, including INH or starting treatment during the recruitment period, hepatitis serology recorded in the medical chart with signed written consent Patients were excluded if they were infected with hepatitis B virus, hepatitis C virus or HIV, had any other hepatic disease, or did not have at least two visits documented in the medical records	348	ATDH
Huang, 2003 (GI: HUANG)	Taiwan	Prospective cohort	Serum ALT, AST and total bilirubin levels were monitored monthly until the end of treatment or checked whenever patients had symptoms of suspected hepatitis	Their standard daily anti-tuberculosis regimen for the first 2 months included INH (300 mg), RMP (600 mg or 450 mg if body weight <50 kg), PZA (20 mg/kg body weight) and EMB (25 mg/kg body weight). PZA was then discontinued, whereas INH, RMP, and EMB (15 mg/kg body weight) were continued for another 4 months	Patients with incident PTB or EPTB Exclusion criteria: 1) abnormal serum ALT, AST or bilirubin before anti-tuberculosis treatment; and 2) refusal of blood sampling or informed written consent	318	ADIH
Jung, 2015	Korea	Prospective cohort	4 weeks	Standard 4-drug treatment for 6 months: INH (5 mg/kg, usually 300 mg), RMP (450 mg for <50 kg or 600 mg for ⩾50 kg of body weight), EMB (15 mg/kg) and PZA (20–30 mg/kg), daily for 2 months, followed by INH and RMP with or without EMB for 4 months. Doses were adjusted when applying model-based treatment	Eligible participants were patients newly diagnosed with active TB, who underwent standard 4-drug treatment for 6 months Exclusion criteria: patients with abnormal hepatic function on laboratory testing (increased serum AST, ALT or total bilirubin) before anti-tuberculosis treatment and underlying liver disease or systemic illness such as congestive heart failure, acute life-threatening disease, or alcoholism or disease that was resistant to INH at the start of treatment	206	ATDH
Khalili, 2011	Iran	Case-control	2 months	Treated daily with INH (300 mg), RMP (600 mg), PZA (20 mg/kg), EMB (15 mg/kg) for the first 2 months, followed by INH and RMP daily for 4 additional months	Inclusion criteria: newly diagnosed patients (>18 years) with active PTB, who had been planned to be treated daily with anti-tuberculosis treatment (see drugs and dosage) Exclusion criteria: other possibilities of hepatitis, including patients with HIV infection, hepatic insufficiency (ALT or AST > 2× ULN) or clinically signs and symptoms of liver diseases such as jaundice and ascites, chronic hepatitis (B or C) and renal insufficiency (creatinine clearance < 50 ml/min based on Cockcroft-Gault equation) and history or current use of herbal, supplemental and hepatotoxic non-tuberculosis drugs	100	Hepatotoxicity
Kim, 2009 (GI: KIM)	Korea	Case-control	Assessments performed 2 weeks after onset of treatment and bi-monthly thereafter	All patients with PTB were treated daily with a combination regimen comprising INH (300–400 mg daily), RMP (450–600 mg daily), EMB (600–800 mg daily) and PZA (1000–1500 mg daily) for 2 months and then without PZA for ⩾4 following months. Doses of each drug were adjusted based on body weight of the patient	Newly diagnosed and treated patients with PTB. Exclusion criteria: patients with active or chronic hepatitis, including alcoholic hepatitis, fatty liver disease, liver cirrhosis, carriers of the hepatitis B or C virus, heavy alcohol intake, decreased renal function and severe cardiac diseases requiring several medications	226	ATDH
Kim, 2011 (GI: KIM)	Korea	Case-control	NR	The treatment comprised an initial phase of 2 months and a subsequent continuation phase of ⩾4 months. During the initial phase, 4 drugs were administered, including INH (300–400 mg daily), RMP (450–600 mg daily), EMB (600–800 mg daily) and PZA (1000–1500 mg daily). Doses of each drug were adjusted based on the body weight of the patient. In the following continuation phase, only PZA was discontinued, while the other three drugs were continued	Patients newly diagnosed with PTB and/or TB pleuritis and treated with first-line anti-tuberculosis medications such as INH, RMP, EMB and PZA Exclusion criteria: 1) patients with skin diseases before treatment, 2) chronic renal failure and chronic liver diseases affecting drug metabolism, 3) chronic alcoholism, 4) other chronic medical conditions requiring medication, and 5) non-adherence to treatment	221	ATD-induced MPE
Lee, 2010	Taiwan	Prospective cohort	NR	All patients received oral INH 300 mg, RMP 600 mg (or 450 mg, if body weight was <50 kg), PZA 200 mg/kg of body weight and EMB 800 mg daily for the first 2 months. PZA was then discontinued, while INH, RMP and EMB were continued for another 4 months	Inclusion criteria: adult patients newly diagnosed with active TB, having evident lesions of TB using simple X-ray, computed tomography, sputum smears and cultures positive for mycobacteria Exclusion criteria: 1) positive serum hepatitis B virus surface antigen, antibody to hepatitis C virus; 2) alcoholic liver disease or habitual alcohol drinking; 3) any other hepatic or systemic diseases that may cause liver dysfunction; 4) abnormal serum ALT, AST or bilirubin levels before anti-tuberculosis treatment	140	ATDH
Leiro-Fernandez, 2011	Spain	Case-control	Routine follow-up of clinical assessments every 2 weeks during the first month and monthly thereafter untilcompletion of treatment	Treatment with regimens that included INH, RMP and PZA at the usual dosages (INH 5 mg/kg/day to maximum 300 mg/day, RMP10 mg/kg/day to maximum 600 mg/day and PZA 25–30 mg/kg/day to maximum 2500 mg/day	Inclusion criteria: 1) age 15–75 years; 2) microbiological demonstration of active TB; 3) treatment with regimens that included INH, RMP and PZA at the usual dosages (INH 5 mg/kg/day to max 300 mg/day, RMP 10 mg/kg/day to max 600 mg/day and PZA 25–30 mg/kg/day to max 2500 mg/day); and 4) adequate treatment adherence Exclusion criteria: 1) increased baseline serum transaminases (AST and/or ALT; normal values ⩽ 40 IU/l); 2) positive serological testing for HIV, hepatitis B virus or hepatitis C virus; 3) regular alcohol intake or concomitant use of hepatotoxic drugs; 4) history of chronic liver disease; 5) pregnancy; or 6) no or poor adherence to treatment	117	ATDH
Lv, 2012	China	Case-control	6–9 months	All primary/retreatment patients took INH (600 mg), RMP (600 mg or 450 mg if body weight was <50 kg), PZA (2000 mg) and EMB (1250 mg) every other day in the first 2 months and then INH and RMP were continued for another 4/6 months. The retreatment patients received SM (750 mg) every other day in the first 2 months and continued receiving EMB for another 6 months	Inclusion criteria: sputum smear-positive PTB patients who received standard short-course chemotherapy recommended by the WHO Exclusion criteria: 1) positive serum hepatitis B virus surface antigen or other liver disease, 2) potentially hepatotoxic medications that would confound the picture, 3) abnormal serum ALT, AST or total bilirubin levels before anti-tuberculosis treatment	445	ATDH
Mahmoud, 2012	Tunisia	Prospective cohort	3 months	Treated with INH and RMP containing regimen	Inclusion criteria: adult patients (>18 years) newly diagnosed with TB treated with INH- and RMP-containing regimen, LFTs before initiation of treatment showed completely normal findings on serum ALT, AST, total bilirubin, ALP and gGTP Exclusion criteria: 1) patients receiving other potentially hepatotoxic drugs in addition to anti-tuberculosis agents; 2) positive serum hepatitis B surface antigen or hepatitis C antibody; 3) alcoholic liver disease; 4) any other hepatic or systemic diseases that may cause liver dysfunction	66	INH hepatotoxicity
Ng, 2014	Cases recruited in the UK; controls were samples from elsewhere	Case-control	NR	All had been prescribed INH with all but one also taking additional anti-tuberculosis drugs. Patients were prescribed 300 mg INH/day in line with UK/WHO guidelines	Cases: patients diagnosed with DILI either in the past or at the time of sample collection. Onlycases assessed as having DILI that was highly probably, probably or possibly relating to anti-tuberculosis drug exposure were enrolled in the study. Other possible causes of liver toxicity, particularly hepatitis A, B, C and CMV infection were excluded. No patients included were HIV-positive or had pre-existing liver disease. None had been treated for TB under directly observed treatment Controls: 52 DNA samples from individuals of European ancestry; inclusion/exclusion criteria not reported	127	DILI
Ohno, 2000	Japan	Prospective cohort	3 months	Initial chemotherapy always included INH (400 mg/day; 8.2 ± 2.0 mg/kg/day) and RMP (450 mg/day; 9.2 ± 2.2 mg/kg/day); the third drug used was EMB or SM.	Inclusion criteria: patients had no history of alcohol abuse and were negative for hepatitis B surface antigen and hepatitis C antibody. At the beginning of treatment, LFTs showed completely normal findings on serum AST, ALT, bilirubin, ALP and gGTP Patients receiving other potentially hepatopathic drugs in addition to anti-tuberculosis agents were excluded from the study	77	INH + RMP-induced hepatotoxicity
Possuelo, 2008 (GI: POSSUELO)	Brazil	Prospective cohort	NR	Treatment daily with INH, RMP and PZA for the first 2 months followed by INH and RMP daily for 4 additional months. Drug dosages used were calculated according to patient's weight (weight <45 kg: RMP 300 mg, INH 200 mg, PZA 1000 mg; 45–55 kg: RMP 450 mg, INH 300 mg, PZA 1500 mg; >55 kg: RMP 600 mg, INH 400 mg, PZA 2000 mg)	Inclusion criteria: adult patients (>18 years) newly diagnosed with active TB, who had been treated daily with anti-tuberculosis treatment (see drugs and dosage) Exclusion criteria: patients presenting clinically and laboratory-confirmed liver chronic disease, patients using anti-tuberculosis drugs before enrolment in the study, patients presenting results of LFTs before the beginning of treatment higher that were 2× ULN and refusal to participate of the study	254	ATDH Gastrointestinal ADRs
Rana, 2014 (GI: RANA)	India	Prospective cohort	Patients were monitored every month until the end of treatment or whenever the patients had symptoms or signs of hepatotoxicity	Daily anti-tuberculosis treatment for the first 2 months included INH (300 mg), RMP (600 or 450 mg for body weight/50 kg), PZA (20 mg/kg body weight) and EMB (25 mg/kg body weight). After 2 months, EMB and PZA were discontinued, whereas INH and RMP were continued for an additional 4 months	Inclusion criteria: patients with pulmonary and EPTB Exclusion criteria: patients with history of alcohol abuse and/or any other liver disease; patients who had received other potentially hepatotoxic drugs in addition to anti-tuberculosis drugs; patients who had abnormal serum ALT, AST or bilirubin before starting anti-tuberculosis treatment; patientswho developed viral hepatitis during anti-tuberculosis treatment; patients with renal failure or cancer; and patients who refused blood sampling or providing informed written consent	300	Hepatotoxicity
Santos, 2013 (GI: SANTOS)	Brazil	Prospective cohort	NR	Treatment with INH, RMP and PZA for the first 2 months, followed by INH and RMP daily for 4 months	Inclusion criteria: patients diagnosed with TB and treated with anti-tuberculosis treatment (see drugs and dosage) Exclusion criteria: patients aged <18 years, those with mental disabilities, chronic liver disease confirmed by clinical and laboratory data, users of anti-tuberculosis drugs before enrolment in the study, and those with liver function results > 2× ULN before beginning treatment	270	ATDH
Shimizu, 2006	Japan	Prospective cohort	3 months	Treatment with INH (300–400 mg) and RMP (300–450 mg)	Individuals with PTB without abnormal serum levels of ALT, AST, direct or total bilirubin, or serum hepatitis B virus surface antigen or antibody to hepatitis C virus at the beginning of the study	42	Hepatotoxicity
Singla, 2014	India	Prospective cohort	NR	NR	Inclusion criteria: newly diagnosed patients with TB Exclusion criteria: history of heavy use of alcohol or chronic liver diseases or liver cirrhosis;patients who were settled in the area for a minimum of three generations; people infected with HIV	408	ATDH
Sotsuka, 2011	Japan	Prospective cohort	3 months	INH, RMP and PZA, plus EMB or SM during the first 2 months, followed by administration of INH and RMP plus EMB or SM during the final 4 months	Inclusion criteria: in-patients with active PTB who were treated with the standard Japanese chemotherapy regimen, followed up for >3 months after treatment, and who consented to this study	144	Hepatotoxicity
Teixeira, 2011	Brazil	Case-control	NR	Anti-tuberculosis drug regimens that include INH at the usual dosage (400 mg/day)	Inclusion criteria: 1) age > 18 years, 2) diagnosis of active TB, 3) treatment with anti-tuberculosis drug regimens that include INH at the usual dosage (400 mg/day) and 4) normal baseline serum transaminases (ALT and AST) before treatment Exclusion criteria: 1) positive serological test for the HIV, hepatitis B virus or hepatitis C virus, 2) alcohol abuse, 3) history of chronic liver disease and 4) pregnancy	167	Hepatitis
Vuilleumier, 2006	Switzerland	Prospective cohort	NR	INH 300 mg daily and vitamin B6 40 mg per day for a period of 6 months	Inclusion criteria: patients having LTBI as defined by the American Thoracic Society with normal plasma AST and ALT levels before the beginning of INH monotherapy Exclusion criteria: 1) a history of alcohol consumption, 2) positive serology for the HAV, HBV or HCV, 3) poor chemotherapy compliance (negative urine INH 3 times during the follow-up), loss during the follow-up, 4) patients had to receive supplementary anti-tuberculosis agents or other potentially hepatotoxic drugs	89	INH-induced hepatotoxicity
Wang, 2015 (GI: NTUH)	Taiwan	Prospective cohort	6 months	Daily INH, RMP, EMB and PZA in the first 2 months, and daily INH and RMP for the next 4 months The daily dosage of each drug was calculated by weight	Adult Taiwanese (>16 years) patients with culture-confirmed PTB Subjects were excluded if they were pregnant, had a life expectancy < 6 months, had abnormal baseline LFT or were resistant to INH, RMP or both	355 in the derivation cohort, 182 in the validation cohort	Hepatotoxicity during anti-tuberculosis treatment
Xiang, 2014	China	Prospective cohort	2 months	All patients were prescribed INH (600 mg), RMP (600 mg or 450 mg if the body weight was <50 kg), PZA (2000 mg) and EMB (1250 mg) every other day in the first 2 months. After 2 months, INH and RMP were continued for a further 4–6 months. Retreatment patients in addition received SM (750 mg) every other day in the first 2 months and continued receiving EMB for another 6 months	Inclusion criteria: newly diagnosed PTB patients belonging to the Uyghur ethnic group, who were receiving standard short-course chemotherapy recommended by the WHO, who attended for a 2-month assessment, and any patients attending clinic with suspected liver disease after the start of treatment, before the 2-month visit Exclusion criteria: patients who had signs of abnormal liver function when they started treatment (jaundice or elevated ALT, AST or bilirubin levels) or disease associated with liver dysfunction	2244	ATLI
Yamada, 2009 (GI: YAMADA)	Canada	Prospective cohort	9 months	INH 300 mg	Subjects who were receiving treatment with INH 300 mg daily self-administered for LTBI.Subjects were included if they were aged ⩾19 years; not concurrently receiving other anti-tuberculosis drugs; non-reactive to hepatitis B surface antigen and antibody to hepatitis C virus; absence of any liver or metabolic diseases; without a HIV-positive test result; not consuming ⩾7 alcoholic beverages per day and undergoing sufficiently frequentAST tests to detect hepatotoxicity	170	Hepatotoxicity
Yimer, 2011	Ethiopia	Prospective cohort	followed up for development of DILI for up to 56 weeks	All study participants received RMP based short-course chemotherapy for TB following the national TB treatment guideline. ART was then initiated	Newly diagnosed ART and anti-tuberculosis treatment-naïve adult TB and HIV co-infected patients. The eligibility criteria were age >18 years, CD4 count, 200 cells/Ul, not pregnant and not on other known hepatotoxic drugs concurrently (except cotrimoxazole, 960 mg per day, which was given to all participants before enrolment and during the follow-up period according to the treatment guideline)	353	Anti-tubercular and antiretroviral DILI
Yuliwulandari, 2016	Indonesia	Case-control	NR	NR (anti-tuberculosis treatment)	Patients included in the study were male and female aged 15–70 years, suffering from TB according to the WHO standards, and were under monitored treatment with anti-tuberculosis drugs Exclusion criteria: 1) history of liver disease such as: hepatitis A B or C; hepatoma; liver cirrhosis or cholelithiasis positive; 2) abnormal levels of any LFT (ALT, AST or total bilirubin) before anti-tuberculosis treatment	241	ATLI
Zaverucha-do-Valle, 2014	Brazil	Retrospective cohort	Follow-up at days 15 and 30 and at least one visit monthly until the end of treatment	600 mg/day of RMP, 400 mg/day of INH and 2 g/day of PZA for all patients with corporal weight >45 kg or adjusted for corporal weight <45 kg. After 2 months of treatment, PZA was discontinued	Inclusion criteria: signed written consent, sputum smear with AFB or culture positive for M. tuberculosis; ongoing TB treatment and laboratory tests of liver function Exclusion criteria: age <18 years, pregnancy and no >1 visit registered	131	ATDH

INH =isoniazid; RMP =rifampicin; PZA =pyrazinamide; EMB =ethambutol; ALT =alanine aminotransferase; AST =aspartate aminotransferase; RCT =randomised controlled trial; HIV =human immunodeficiency virus; SM =streptomycin; PTB =pulmonary tuberculosis; IU =international unit; ALP =alkaline phosphatase; DILI =drug-induced liver injury; LFT =liver function test; DIH =drug-induced hepatotoxicity; NR =not reported; ATDH =anti-tuberculosis drug-induced hepatotoxicity; GI =group identifier; ADR =adverse drug reaction; ULN =upper limit of normal; EPTB =extra-pulmonary TB; ADIH =anti-tuberculosis drug-induced hepatitis; ATD =anti-tuberculosis drug; MPE =maculopapular eruption; WHO = World Health Organization; gGTP = gamma-glutamyltranspeptidase; CMV = cytomegalovirus ; LTBI = latent tuberculous infection; ART =antiretroviral therapy; ATLI = anti-tuberculosis drug-induced liver injury.

**Table A.3:** Key characteristics of included studies

Author, year	Country	Study design	Follow-up time	Drugs and dosage	Selection criteria	Sample size *n*	Toxicity outcomes
An, 2012	China	App	6 months	Daily treatment with INH, RMP, PZA and EMB for 2 months, followed by 4 months treatment with INH and RMP, with drug dosages calculated according to body weight Body weight < 45 kg: RMP 300 mg, INH 200 mg, PZA 1000 mg Body weight of 45–55 kg: RMP 450 mg, INH 300 mg, PZA 1500 mg Body weight > 55 kg: RMP 600 mg, INH 400 mg, PZA 2000 mg	Inclusion criteria: Normal serum ALT, AST and bilirubin levels, no symptoms related to abnormal liver function (i.e., jaundice) before anti-tuberculosis drug treatment and close monitoring of changes in liver functions within 6 months of treatment Patients with and without hepatotoxicity during treatment Exclusion criteria: Malnutrition HIV type 1 infection Alcoholic liver disease or habitual drinking Hepatitis B or C infection, liver disease, systemic diseases and/or treatment with drugs other than the anti-tuberculosis drugs that can induce hepatotoxicity Severe TB or cardiac dysfunction that may cause liver dysfunction Transient increases in ALT	208	Hepatotoxocity
Azuma, 2013	Japan	RCT	6 months	All patients were treated with a 6-month regimen comprising INH, RMP, PZA and EMB/SM for the first 2 months, followed by RMP and INH for 4 months. All patients were started on the standard oral dose (~5 mg/kg body weight, once-daily). For pharmacogenetics-treatment patients, dosages were adjusted based on individual *NAT2* status within 3 days. Modified daily INH doses were respectively ~7.5, ~5 and ~2.5 mg/kg for rapid, intermediate and slow acetylators. Regarding the other drugs for the standard regimen, standard daily doses of RMP (10 mg/kg, maximum 600 mg/body), PZA (25 mg/kg, 1500 mg/body), EMB (15 mg/kg, 750 mg/body; 20 mg/kg, 1000 mg/body) and SM (15 mg/kg, 750 mg/body) were recommended with the following dose ranges allowed at the discretion of the physician in charge: RMP 8–12 mg/kg; PZA 20–30 mg/kg; EMB 15–20 mg/kg; SM 12–18 mg/kg	The eligible population was identified from a series of patients newly diagnosed with PTB (male and female aged 20–75 years), requiring the 6-month 4-drug standard treatment for the first time Exclusion criteria: abnormal test results for liver and kidney function (serum AST > 45 IU/l, ALT > 50 IU/l, ALP > 444 IU/l, total bilirubin > 1.6 mg/dl and creatinine > 1.4 mg/dl before the study treatments commenced, long-term use of steroids and/or immunosuppressants, inadequate clinical conditions such as hyperglycaemia, diabetes mellitus, acute life-threatening chronic progressive disease, pregnancy or lactation and alcoholism. Patients not expected to complete the study protocol for social reasons were not recruited	172	INH-DILI Peripheral neuropathy
Bose, 2011	India	Prospective cohort	Patients were followed up to 8 weeks after they had started with anti-tuberculosis treatment	All patients received anti-tuberculosis treatment (RMP, INH, EMB and PZA) according to body weight. All four drugs were given for 2 months. PZA and EMB were discontinued, while INH and RMP were continued for another 4 months RMP doses as follows: ⩽35 kg: 300 mg/day 36–50 kg: 450 mg/day >50 kg: 600 mg/day INH doses as follows: ⩽35 kg: 200 mg/day >35 kg: 400 mg/day PZA doses as follows: ⩽50 kg: 1.0 g/day >50 kg: 1.5 g/day EMB 20 mg/kg/day	Newly diagnosed patients of PTB. The baseline LFT of the patients was normal when they were started on anti-tuberculosis treatment Exclusion criteria: no history of habitual alcoholism, chronic liver diseases or steatosis	218	ADIH ADIH outcome
Çetintaş, 2008	Turkey	Prospective cohort	NR	Patients received INH 5 mg/kg (maximum 300 mg/day), RMP 10 mg/kg (maximum 600 mg/day), PZA 25 mg/kg (maximum 2000 mg/day), EMB 15–25 mg/kg (maximum 1500 mg/day)	Patients diagnosed with TB. Only patients with serum levels before initiation of treatment within the following ranges were included in the study: ALT 0–40 U/l, AST 5–45 U/l and total bilirubin 0.2–1.6 mg/dl Exclusion criteria: patients with positive serum hepatitis B surface antigen, hepatitis C antibody or hepatitis A immunoglobulin M antibody, and patients with alcoholic liver disease or any hepatic or systemic disease that could cause liver function disorder	100	DIH
Chamorro, 2013	Argentina	Prospective cohort	NR	The patients began a standard anti-tuberculosis treatment protocol for the first 2 months (INH 5 mg/kg/days, maximum 300 mg/day, RMP 10 mg/kg/day maximum 600 mg/day, PZA 20 mg/kg/day, EMB 20 mg/kg/day), followed by INH and RMP for ⩾4 months, depending on the disease severity or the presence ofextra-pulmonary foci	Inclusion criteria: TB patients aged >18 years with stable haemodynamic levels, normal renal function and negative for pregnancy Exclusion criteria: presence of diseases that directly affected the liver (acute hepatitis A, active hepatitis B and C; cirrhosis; encephalopathy and cancer), INH allergy, HIV, autoimmunity, concomitant hepatotoxic medications, a history of TB treatment failures, refusal of blood extraction and refusal to sign the written informed consent	175	ATDH
Chang, 2012	Taiwan	Prospective cohort	NR	First-line anti-tuberculosis medications	Patients diagnosed with TB and treated with first-line anti-tuberculosis medications Exclusion criteria: pregnancy, abnormal liver function and history of positive viral hepatitis before starting treatment with first-line anti-tuberculosis drugs	98	ATDH
Cho, 2007	Korea	Prospective cohort	Serum AST, ALT and total bilirubin levels were then monitored monthly until the end of treatment	All patients received oral INH (300 mg), RMP (600 mg), PZA (20 mg/kg body weight) and EMB (800 mg) daily for the first 2 months. PZA was then discontinued, while INH, RMP and EMB were continued for another 4 months	Adult patients newly diagnosed with active TB, with evident lesion of TB using simple X-ray or computed tomography or positive results on sputum smear or culture for detection of mycobacteria Exclusion criteria: 1) abnormal serum ALT, AST, or bilirubin levels or symptoms related to abnormal liver function such as jaundice before anti-tuberculosis treatment; 2) alcoholic liver disease or habitual alcohol drinking; 3) any other hepatic or systemic diseases that may cause liver dysfunction	132	ATDH
Costa, 2012	Brazil	Prospective cohort	NR	All patients were treated with the first-line anti-tuberculosis drug regimen INH (300 mg/kg/day), RMP (300 mg/kg/day) and PZA (1500 mg/kg/day) for the first 2 months, followed by INH and RMP for a further 4 months	Male or female subjects aged ⩾18 years, with no previously described renal, allergic or hepatic diseases and not pregnant	129	ADRs
Dhoro, 2013	Zimbabwe	Case-control	NR	NR	TB patients	Not clear—189 received INH	Peripheral neuropathy
Feng, 2014	China	Case-control	6 months	Treatment with anti-tuberculosis drug regimens at the usual dosage—300 mg/day INH, 450 mg/day RMP and 1500 mg/day PZA	Selection of cases: cases selected based on liver functions, i.e., all indices of liver function were normal before anti-tuberculosis chemotherapy, and became abnormal indicating hepatic injury after 6 months of chemotherapy. Cases were patients who showed ATDH based on increased serum transaminase values that were three-fold higher than the ULN (40 IU/l ALT) and symptoms compatible with hepatitis Selection of controls: controls underwent the same anti-tuberculosis chemotherapy with selected cases and were not tested for abnormality in liver functions after 6 months of the chemotherapy. The controls selected matched the criteria compared to the cases: 1) sex; 2) age discrepancy of <5 years; 3) living in the same regions; and 4) treatment with anti-tuberculosis drug regimens at the usual dosage, including 300 mg/day INH, 450 mg/day RMP and 1500 mg/day PZA	346	ATDH
Fredj, 2016	Tunisia	Prospective cohort	Serum AST, ALT and ALP were monitored monthly until the end of the treatment	The anti-tuberculosis treatment was based on the association of INH (5 mg/kg/day), RMP (10 mg/kg/day), PZA (25 mg/kg/day) and EMB (15 mg/kg/day) for the first 2 months, followed by INH and RMP for 4–7 additional months, depending on TB clinical presentation	Patients diagnosed with PTB and EPTB	71	INH-induced-hepatotoxicity
Gupta, 2013 (GI: GUPTA)	India	Prospective cohort	The patients were monitored for ALT, AST, andtotal bilirubin levels weekly for 1 month and then monthly until the completion of treatment	Initially patients received a combination regimen including INH 5 mg/kg (max 300 mg daily), RMP 10 mg/kg (max 600 mg daily), PZA 25 mg/kg (max 1500 mg daily) and EMB 15–25 mg/kg (max 2000 mg daily) for a period of 2 months, followed by an additional 4 months with INH and RMP	Inclusion criteria: 1) age > 18 years; 2) smear and/or culture positive for mycobacteria in clinical samples; and 3) normal ALT, AST and total bilirubin levels Exclusion criteria: 1) patients presenting clinically and laboratory-confirmed chronic liver disease such as jaundice; 2) acute and chronic hepatitis B and/or C or HIV; 3) alcoholic liver diseases; 4) a rise of two times the ULN of ALT, AST and total bilirubin levels; 5) medication with anti-tuberculosis drugs before start of treatment and/or other potentially hepatotoxic drugs; and 6) refusal to provide blood sample or signed informed consent form	215	ATDH
Higuchi, 2007 (GI: HIGUCHI)	Japan	Prospective cohort	NR	Treated with a INH (400 mg/d) and RMP (450 mg/d) containing regimen for 6 or 9 months	Patients with new onset of PTB treated with a INH (400 mg/d) and RMP (450 mg/d) containing regimen for 6 or 9 months Exclusion criteria: patients with liver cirrhosis, chronic and acute hepatitis, alcoholic liver disease and other chronic liver diseases	100	ATDH Skin rash Eosinophilia
Ho, 2013	Taiwan	Prospective cohort	180 days	All patients received oral INH 300 mg, RMP 600 mg (or 450 mg if body weight was <50 kg), PZA 25 mg/kg of body weight (max daily dose 2000 mg) and EMB 15 mg/kg of body weight daily (max daily dose 1600 mg) for the first 2 months. PZA was then discontinued, whereas INH, RMP and EMB were continued for another 4 months	The inclusion criteria included a sputum smear with AFB or culture positive for M. tuberculosis, undergoing anti-tuberculosis treatment, including INH or starting treatment during the recruitment period, hepatitis serology recorded in the medical chart with signed written consent Patients were excluded if they were infected with hepatitis B virus, hepatitis C virus or HIV, had any other hepatic disease, or did not have at least two visits documented in the medical records	348	ATDH
Huang, 2003 (GI: HUANG)	Taiwan	Prospective cohort	Serum ALT, AST and total bilirubin levels were monitored monthly until the end of treatment or checked whenever patients had symptoms of suspected hepatitis	Their standard daily anti-tuberculosis regimen for the first 2 months included INH (300 mg), RMP (600 mg or 450 mg if body weight <50 kg), PZA (20 mg/kg body weight) and EMB (25 mg/kg body weight). PZA was then discontinued, whereas INH, RMP, and EMB (15 mg/kg body weight) were continued for another 4 months	Patients with incident PTB or EPTB Exclusion criteria: 1) abnormal serum ALT, AST or bilirubin before anti-tuberculosis treatment; and 2) refusal of blood sampling or informed written consent	318	ADIH
Jung, 2015	Korea	Prospective cohort	4 weeks	Standard 4-drug treatment for 6 months: INH (5 mg/kg, usually 300 mg), RMP (450 mg for <50 kg or 600 mg for ⩾50 kg of body weight), EMB (15 mg/kg) and PZA (20–30 mg/kg), daily for 2 months, followed by INH and RMP with or without EMB for 4 months. Doses were adjusted when applying model-based treatment	Eligible participants were patients newly diagnosed with active TB, who underwent standard 4-drug treatment for 6 months Exclusion criteria: patients with abnormal hepatic function on laboratory testing (increased serum AST, ALT or total bilirubin) before anti-tuberculosis treatment and underlying liver disease or systemic illness such as congestive heart failure, acute life-threatening disease, or alcoholism or disease that was resistant to INH at the start of treatment	206	ATDH
Khalili, 2011	Iran	Case-control	2 months	Treated daily with INH (300 mg), RMP (600 mg), PZA (20 mg/kg), EMB (15 mg/kg) for the first 2 months, followed by INH and RMP daily for 4 additional months	Inclusion criteria: newly diagnosed patients (>18 years) with active PTB, who had been planned to be treated daily with anti-tuberculosis treatment (see drugs and dosage) Exclusion criteria: other possibilities of hepatitis, including patients with HIV infection, hepatic insufficiency (ALT or AST > 2× ULN) or clinically signs and symptoms of liver diseases such as jaundice and ascites, chronic hepatitis (B or C) and renal insufficiency (creatinine clearance < 50 ml/min based on Cockcroft-Gault equation) and history or current use of herbal, supplemental and hepatotoxic non-tuberculosis drugs	100	Hepatotoxicity
Kim, 2009 (GI: KIM)	Korea	Case-control	Assessments performed 2 weeks after onset of treatment and bi-monthly thereafter	All patients with PTB were treated daily with a combination regimen comprising INH (300–400 mg daily), RMP (450–600 mg daily), EMB (600–800 mg daily) and PZA (1000–1500 mg daily) for 2 months and then without PZA for ⩾4 following months. Doses of each drug were adjusted based on body weight of the patient	Newly diagnosed and treated patients with PTB. Exclusion criteria: patients with active or chronic hepatitis, including alcoholic hepatitis, fatty liver disease, liver cirrhosis, carriers of the hepatitis B or C virus, heavy alcohol intake, decreased renal function and severe cardiac diseases requiring several medications	226	ATDH
Kim, 2011 (GI: KIM)	Korea	Case-control	NR	The treatment comprised an initial phase of 2 months and a subsequent continuation phase of ⩾4 months. During the initial phase, 4 drugs were administered, including INH (300–400 mg daily), RMP (450–600 mg daily), EMB (600–800 mg daily) and PZA (1000–1500 mg daily). Doses of each drug were adjusted based on the body weight of the patient. In the following continuation phase, only PZA was discontinued, while the other three drugs were continued	Patients newly diagnosed with PTB and/or TB pleuritis and treated with first-line anti-tuberculosis medications such as INH, RMP, EMB and PZA Exclusion criteria: 1) patients with skin diseases before treatment, 2) chronic renal failure and chronic liver diseases affecting drug metabolism, 3) chronic alcoholism, 4) other chronic medical conditions requiring medication, and 5) non-adherence to treatment	221	ATD-induced MPE
Lee, 2010	Taiwan	Prospective cohort	NR	All patients received oral INH 300 mg, RMP 600 mg (or 450 mg, if body weight was <50 kg), PZA 200 mg/kg of body weight and EMB 800 mg daily for the first 2 months. PZA was then discontinued, while INH, RMP and EMB were continued for another 4 months	Inclusion criteria: adult patients newly diagnosed with active TB, having evident lesions of TB using simple X-ray, computed tomography, sputum smears and cultures positive for mycobacteria Exclusion criteria: 1) positive serum hepatitis B virus surface antigen, antibody to hepatitis C virus; 2) alcoholic liver disease or habitual alcohol drinking; 3) any other hepatic or systemic diseases that may cause liver dysfunction; 4) abnormal serum ALT, AST or bilirubin levels before anti-tuberculosis treatment	140	ATDH
Leiro-Fernandez, 2011	Spain	Case-control	Routine follow-up of clinical assessments every 2 weeks during the first month and monthly thereafter untilcompletion of treatment	Treatment with regimens that included INH, RMP and PZA at the usual dosages (INH 5 mg/kg/day to maximum 300 mg/day, RMP10 mg/kg/day to maximum 600 mg/day and PZA 25–30 mg/kg/day to maximum 2500 mg/day	Inclusion criteria: 1) age 15–75 years; 2) microbiological demonstration of active TB; 3) treatment with regimens that included INH, RMP and PZA at the usual dosages (INH 5 mg/kg/day to max 300 mg/day, RMP 10 mg/kg/day to max 600 mg/day and PZA 25–30 mg/kg/day to max 2500 mg/day); and 4) adequate treatment adherence Exclusion criteria: 1) increased baseline serum transaminases (AST and/or ALT; normal values ⩽ 40 IU/l); 2) positive serological testing for HIV, hepatitis B virus or hepatitis C virus; 3) regular alcohol intake or concomitant use of hepatotoxic drugs; 4) history of chronic liver disease; 5) pregnancy; or 6) no or poor adherence to treatment	117	ATDH
Lv, 2012	China	Case-control	6–9 months	All primary/retreatment patients took INH (600 mg), RMP (600 mg or 450 mg if body weight was <50 kg), PZA (2000 mg) and EMB (1250 mg) every other day in the first 2 months and then INH and RMP were continued for another 4/6 months. The retreatment patients received SM (750 mg) every other day in the first 2 months and continued receiving EMB for another 6 months	Inclusion criteria: sputum smear-positive PTB patients who received standard short-course chemotherapy recommended by the WHO Exclusion criteria: 1) positive serum hepatitis B virus surface antigen or other liver disease, 2) potentially hepatotoxic medications that would confound the picture, 3) abnormal serum ALT, AST or total bilirubin levels before anti-tuberculosis treatment	445	ATDH
Mahmoud, 2012	Tunisia	Prospective cohort	3 months	Treated with INH and RMP containing regimen	Inclusion criteria: adult patients (>18 years) newly diagnosed with TB treated with INH- and RMP-containing regimen, LFTs before initiation of treatment showed completely normal findings on serum ALT, AST, total bilirubin, ALP and gGTP Exclusion criteria: 1) patients receiving other potentially hepatotoxic drugs in addition to anti-tuberculosis agents; 2) positive serum hepatitis B surface antigen or hepatitis C antibody; 3) alcoholic liver disease; 4) any other hepatic or systemic diseases that may cause liver dysfunction	66	INH hepatotoxicity
Ng, 2014	Cases recruited in the UK; controls were samples from elsewhere	Case-control	NR	All had been prescribed INH with all but one also taking additional anti-tuberculosis drugs. Patients were prescribed 300 mg INH/day in line with UK/WHO guidelines	Cases: patients diagnosed with DILI either in the past or at the time of sample collection. Onlycases assessed as having DILI that was highly probably, probably or possibly relating to anti-tuberculosis drug exposure were enrolled in the study. Other possible causes of liver toxicity, particularly hepatitis A, B, C and CMV infection were excluded. No patients included were HIV-positive or had pre-existing liver disease. None had been treated for TB under directly observed treatment Controls: 52 DNA samples from individuals of European ancestry; inclusion/exclusion criteria not reported	127	DILI
Ohno, 2000	Japan	Prospective cohort	3 months	Initial chemotherapy always included INH (400 mg/day; 8.2 ± 2.0 mg/kg/day) and RMP (450 mg/day; 9.2 ± 2.2 mg/kg/day); the third drug used was EMB or SM.	Inclusion criteria: patients had no history of alcohol abuse and were negative for hepatitis B surface antigen and hepatitis C antibody. At the beginning of treatment, LFTs showed completely normal findings on serum AST, ALT, bilirubin, ALP and gGTP Patients receiving other potentially hepatopathic drugs in addition to anti-tuberculosis agents were excluded from the study	77	INH + RMP-induced hepatotoxicity
Possuelo, 2008 (GI: POSSUELO)	Brazil	Prospective cohort	NR	Treatment daily with INH, RMP and PZA for the first 2 months followed by INH and RMP daily for 4 additional months. Drug dosages used were calculated according to patient's weight (weight <45 kg: RMP 300 mg, INH 200 mg, PZA 1000 mg; 45–55 kg: RMP 450 mg, INH 300 mg, PZA 1500 mg; >55 kg: RMP 600 mg, INH 400 mg, PZA 2000 mg)	Inclusion criteria: adult patients (>18 years) newly diagnosed with active TB, who had been treated daily with anti-tuberculosis treatment (see drugs and dosage) Exclusion criteria: patients presenting clinically and laboratory-confirmed liver chronic disease, patients using anti-tuberculosis drugs before enrolment in the study, patients presenting results of LFTs before the beginning of treatment higher that were 2× ULN and refusal to participate of the study	254	ATDH Gastrointestinal ADRs
Rana, 2014 (GI: RANA)	India	Prospective cohort	Patients were monitored every month until the end of treatment or whenever the patients had symptoms or signs of hepatotoxicity	Daily anti-tuberculosis treatment for the first 2 months included INH (300 mg), RMP (600 or 450 mg for body weight/50 kg), PZA (20 mg/kg body weight) and EMB (25 mg/kg body weight). After 2 months, EMB and PZA were discontinued, whereas INH and RMP were continued for an additional 4 months	Inclusion criteria: patients with pulmonary and EPTB Exclusion criteria: patients with history of alcohol abuse and/or any other liver disease; patients who had received other potentially hepatotoxic drugs in addition to anti-tuberculosis drugs; patients who had abnormal serum ALT, AST or bilirubin before starting anti-tuberculosis treatment; patientswho developed viral hepatitis during anti-tuberculosis treatment; patients with renal failure or cancer; and patients who refused blood sampling or providing informed written consent	300	Hepatotoxicity
Santos, 2013 (GI: SANTOS)	Brazil	Prospective cohort	NR	Treatment with INH, RMP and PZA for the first 2 months, followed by INH and RMP daily for 4 months	Inclusion criteria: patients diagnosed with TB and treated with anti-tuberculosis treatment (see drugs and dosage) Exclusion criteria: patients aged <18 years, those with mental disabilities, chronic liver disease confirmed by clinical and laboratory data, users of anti-tuberculosis drugs before enrolment in the study, and those with liver function results > 2× ULN before beginning treatment	270	ATDH
Shimizu, 2006	Japan	Prospective cohort	3 months	Treatment with INH (300–400 mg) and RMP (300–450 mg)	Individuals with PTB without abnormal serum levels of ALT, AST, direct or total bilirubin, or serum hepatitis B virus surface antigen or antibody to hepatitis C virus at the beginning of the study	42	Hepatotoxicity
Singla, 2014	India	Prospective cohort	NR	NR	Inclusion criteria: newly diagnosed patients with TB Exclusion criteria: history of heavy use of alcohol or chronic liver diseases or liver cirrhosis;patients who were settled in the area for a minimum of three generations; people infected with HIV	408	ATDH
Sotsuka, 2011	Japan	Prospective cohort	3 months	INH, RMP and PZA, plus EMB or SM during the first 2 months, followed by administration of INH and RMP plus EMB or SM during the final 4 months	Inclusion criteria: in-patients with active PTB who were treated with the standard Japanese chemotherapy regimen, followed up for >3 months after treatment, and who consented to this study	144	Hepatotoxicity
Teixeira, 2011	Brazil	Case-control	NR	Anti-tuberculosis drug regimens that include INH at the usual dosage (400 mg/day)	Inclusion criteria: 1) age > 18 years, 2) diagnosis of active TB, 3) treatment with anti-tuberculosis drug regimens that include INH at the usual dosage (400 mg/day) and 4) normal baseline serum transaminases (ALT and AST) before treatment Exclusion criteria: 1) positive serological test for the HIV, hepatitis B virus or hepatitis C virus, 2) alcohol abuse, 3) history of chronic liver disease and 4) pregnancy	167	Hepatitis
Vuilleumier, 2006	Switzerland	Prospective cohort	NR	INH 300 mg daily and vitamin B6 40 mg per day for a period of 6 months	Inclusion criteria: patients having LTBI as defined by the American Thoracic Society with normal plasma AST and ALT levels before the beginning of INH monotherapy Exclusion criteria: 1) a history of alcohol consumption, 2) positive serology for the HAV, HBV or HCV, 3) poor chemotherapy compliance (negative urine INH 3 times during the follow-up), loss during the follow-up, 4) patients had to receive supplementary anti-tuberculosis agents or other potentially hepatotoxic drugs	89	INH-induced hepatotoxicity
Wang, 2015 (GI: NTUH)	Taiwan	Prospective cohort	6 months	Daily INH, RMP, EMB and PZA in the first 2 months, and daily INH and RMP for the next 4 months The daily dosage of each drug was calculated by weight	Adult Taiwanese (>16 years) patients with culture-confirmed PTB Subjects were excluded if they were pregnant, had a life expectancy < 6 months, had abnormal baseline LFT or were resistant to INH, RMP or both	355 in the derivation cohort, 182 in the validation cohort	Hepatotoxicity during anti-tuberculosis treatment
Xiang, 2014	China	Prospective cohort	2 months	All patients were prescribed INH (600 mg), RMP (600 mg or 450 mg if the body weight was <50 kg), PZA (2000 mg) and EMB (1250 mg) every other day in the first 2 months. After 2 months, INH and RMP were continued for a further 4–6 months. Retreatment patients in addition received SM (750 mg) every other day in the first 2 months and continued receiving EMB for another 6 months	Inclusion criteria: newly diagnosed PTB patients belonging to the Uyghur ethnic group, who were receiving standard short-course chemotherapy recommended by the WHO, who attended for a 2-month assessment, and any patients attending clinic with suspected liver disease after the start of treatment, before the 2-month visit Exclusion criteria: patients who had signs of abnormal liver function when they started treatment (jaundice or elevated ALT, AST or bilirubin levels) or disease associated with liver dysfunction	2244	ATLI
Yamada, 2009 (GI: YAMADA)	Canada	Prospective cohort	9 months	INH 300 mg	Subjects who were receiving treatment with INH 300 mg daily self-administered for LTBI.Subjects were included if they were aged ⩾19 years; not concurrently receiving other anti-tuberculosis drugs; non-reactive to hepatitis B surface antigen and antibody to hepatitis C virus; absence of any liver or metabolic diseases; without a HIV-positive test result; not consuming ⩾7 alcoholic beverages per day and undergoing sufficiently frequentAST tests to detect hepatotoxicity	170	Hepatotoxicity
Yimer, 2011	Ethiopia	Prospective cohort	followed up for development of DILI for up to 56 weeks	All study participants received RMP based short-course chemotherapy for TB following the national TB treatment guideline. ART was then initiated	Newly diagnosed ART and anti-tuberculosis treatment-naïve adult TB and HIV co-infected patients. The eligibility criteria were age >18 years, CD4 count, 200 cells/Ul, not pregnant and not on other known hepatotoxic drugs concurrently (except cotrimoxazole, 960 mg per day, which was given to all participants before enrolment and during the follow-up period according to the treatment guideline)	353	Anti-tubercular and antiretroviral DILI
Yuliwulandari, 2016	Indonesia	Case-control	NR	NR (anti-tuberculosis treatment)	Patients included in the study were male and female aged 15–70 years, suffering from TB according to the WHO standards, and were under monitored treatment with anti-tuberculosis drugs Exclusion criteria: 1) history of liver disease such as: hepatitis A B or C; hepatoma; liver cirrhosis or cholelithiasis positive; 2) abnormal levels of any LFT (ALT, AST or total bilirubin) before anti-tuberculosis treatment	241	ATLI
Zaverucha-do-Valle, 2014	Brazil	Retrospective cohort	Follow-up at days 15 and 30 and at least one visit monthly until the end of treatment	600 mg/day of RMP, 400 mg/day of INH and 2 g/day of PZA for all patients with corporal weight >45 kg or adjusted for corporal weight <45 kg. After 2 months of treatment, PZA was discontinued	Inclusion criteria: signed written consent, sputum smear with AFB or culture positive for M. tuberculosis; ongoing TB treatment and laboratory tests of liver function Exclusion criteria: age <18 years, pregnancy and no >1 visit registered	131	ATDH

INH =isoniazid; RMP =rifampicin; PZA =pyrazinamide; EMB =ethambutol; ALT =alanine aminotransferase; AST =aspartate aminotransferase; RCT =randomised controlled trial; HIV =human immunodeficiency virus; SM =streptomycin; PTB =pulmonary tuberculosis; IU =international unit; ALP =alkaline phosphatase; DILI =drug-induced liver injury; LFT =liver function test; DIH =drug-induced hepatotoxicity; NR =not reported; ATDH =anti-tuberculosis drug-induced hepatotoxicity; GI =group identifier; ADR =adverse drug reaction; ULN =upper limit of normal; EPTB =extra-pulmonary TB; ADIH =anti-tuberculosis drug-induced hepatitis; ATD =anti-tuberculosis drug; MPE =maculopapular eruption; WHO = World Health Organization; gGTP = gamma-glutamyltranspeptidase; CMV = cytomegalovirus ; LTBI = latent tuberculous infection; ART =antiretroviral therapy; ATLI = anti-tuberculosis drug-induced liver injury.

**Table A.3:** (*continued*)

Author, year	Country	Study design	Follow-up time	Drugs and dosage	Selection criteria	Sample size *n*	Toxicity outcomes
An, 2012	China	App	6 months	Daily treatment with INH, RMP, PZA and EMB for 2 months, followed by 4 months treatment with INH and RMP, with drug dosages calculated according to body weight Body weight < 45 kg: RMP 300 mg, INH 200 mg, PZA 1000 mg Body weight of 45–55 kg: RMP 450 mg, INH 300 mg, PZA 1500 mg Body weight > 55 kg: RMP 600 mg, INH 400 mg, PZA 2000 mg	Inclusion criteria: Normal serum ALT, AST and bilirubin levels, no symptoms related to abnormal liver function (i.e., jaundice) before anti-tuberculosis drug treatment and close monitoring of changes in liver functions within 6 months of treatment Patients with and without hepatotoxicity during treatment Exclusion criteria: Malnutrition HIV type 1 infection Alcoholic liver disease or habitual drinking Hepatitis B or C infection, liver disease, systemic diseases and/or treatment with drugs other than the anti-tuberculosis drugs that can induce hepatotoxicity Severe TB or cardiac dysfunction that may cause liver dysfunction Transient increases in ALT	208	Hepatotoxocity
Azuma, 2013	Japan	RCT	6 months	All patients were treated with a 6-month regimen comprising INH, RMP, PZA and EMB/SM for the first 2 months, followed by RMP and INH for 4 months. All patients were started on the standard oral dose (~5 mg/kg body weight, once-daily). For pharmacogenetics-treatment patients, dosages were adjusted based on individual *NAT2* status within 3 days. Modified daily INH doses were respectively ~7.5, ~5 and ~2.5 mg/kg for rapid, intermediate and slow acetylators. Regarding the other drugs for the standard regimen, standard daily doses of RMP (10 mg/kg, maximum 600 mg/body), PZA (25 mg/kg, 1500 mg/body), EMB (15 mg/kg, 750 mg/body; 20 mg/kg, 1000 mg/body) and SM (15 mg/kg, 750 mg/body) were recommended with the following dose ranges allowed at the discretion of the physician in charge: RMP 8–12 mg/kg; PZA 20–30 mg/kg; EMB 15–20 mg/kg; SM 12–18 mg/kg	The eligible population was identified from a series of patients newly diagnosed with PTB (male and female aged 20–75 years), requiring the 6-month 4-drug standard treatment for the first time Exclusion criteria: abnormal test results for liver and kidney function (serum AST > 45 IU/l, ALT > 50 IU/l, ALP > 444 IU/l, total bilirubin > 1.6 mg/dl and creatinine > 1.4 mg/dl before the study treatments commenced, long-term use of steroids and/or immunosuppressants, inadequate clinical conditions such as hyperglycaemia, diabetes mellitus, acute life-threatening chronic progressive disease, pregnancy or lactation and alcoholism. Patients not expected to complete the study protocol for social reasons were not recruited	172	INH-DILI Peripheral neuropathy
Bose, 2011	India	Prospective cohort	Patients were followed up to 8 weeks after they had started with anti-tuberculosis treatment	All patients received anti-tuberculosis treatment (RMP, INH, EMB and PZA) according to body weight. All four drugs were given for 2 months. PZA and EMB were discontinued, while INH and RMP were continued for another 4 months RMP doses as follows: ⩽35 kg: 300 mg/day 36–50 kg: 450 mg/day >50 kg: 600 mg/day INH doses as follows: ⩽35 kg: 200 mg/day >35 kg: 400 mg/day PZA doses as follows: ⩽50 kg: 1.0 g/day >50 kg: 1.5 g/day EMB 20 mg/kg/day	Newly diagnosed patients of PTB. The baseline LFT of the patients was normal when they were started on anti-tuberculosis treatment Exclusion criteria: no history of habitual alcoholism, chronic liver diseases or steatosis	218	ADIH ADIH outcome
Çetintaş, 2008	Turkey	Prospective cohort	NR	Patients received INH 5 mg/kg (maximum 300 mg/day), RMP 10 mg/kg (maximum 600 mg/day), PZA 25 mg/kg (maximum 2000 mg/day), EMB 15–25 mg/kg (maximum 1500 mg/day)	Patients diagnosed with TB. Only patients with serum levels before initiation of treatment within the following ranges were included in the study: ALT 0–40 U/l, AST 5–45 U/l and total bilirubin 0.2–1.6 mg/dl Exclusion criteria: patients with positive serum hepatitis B surface antigen, hepatitis C antibody or hepatitis A immunoglobulin M antibody, and patients with alcoholic liver disease or any hepatic or systemic disease that could cause liver function disorder	100	DIH
Chamorro, 2013	Argentina	Prospective cohort	NR	The patients began a standard anti-tuberculosis treatment protocol for the first 2 months (INH 5 mg/kg/days, maximum 300 mg/day, RMP 10 mg/kg/day maximum 600 mg/day, PZA 20 mg/kg/day, EMB 20 mg/kg/day), followed by INH and RMP for ⩾4 months, depending on the disease severity or the presence ofextra-pulmonary foci	Inclusion criteria: TB patients aged >18 years with stable haemodynamic levels, normal renal function and negative for pregnancy Exclusion criteria: presence of diseases that directly affected the liver (acute hepatitis A, active hepatitis B and C; cirrhosis; encephalopathy and cancer), INH allergy, HIV, autoimmunity, concomitant hepatotoxic medications, a history of TB treatment failures, refusal of blood extraction and refusal to sign the written informed consent	175	ATDH
Chang, 2012	Taiwan	Prospective cohort	NR	First-line anti-tuberculosis medications	Patients diagnosed with TB and treated with first-line anti-tuberculosis medications Exclusion criteria: pregnancy, abnormal liver function and history of positive viral hepatitis before starting treatment with first-line anti-tuberculosis drugs	98	ATDH
Cho, 2007	Korea	Prospective cohort	Serum AST, ALT and total bilirubin levels were then monitored monthly until the end of treatment	All patients received oral INH (300 mg), RMP (600 mg), PZA (20 mg/kg body weight) and EMB (800 mg) daily for the first 2 months. PZA was then discontinued, while INH, RMP and EMB were continued for another 4 months	Adult patients newly diagnosed with active TB, with evident lesion of TB using simple X-ray or computed tomography or positive results on sputum smear or culture for detection of mycobacteria Exclusion criteria: 1) abnormal serum ALT, AST, or bilirubin levels or symptoms related to abnormal liver function such as jaundice before anti-tuberculosis treatment; 2) alcoholic liver disease or habitual alcohol drinking; 3) any other hepatic or systemic diseases that may cause liver dysfunction	132	ATDH
Costa, 2012	Brazil	Prospective cohort	NR	All patients were treated with the first-line anti-tuberculosis drug regimen INH (300 mg/kg/day), RMP (300 mg/kg/day) and PZA (1500 mg/kg/day) for the first 2 months, followed by INH and RMP for a further 4 months	Male or female subjects aged ⩾18 years, with no previously described renal, allergic or hepatic diseases and not pregnant	129	ADRs
Dhoro, 2013	Zimbabwe	Case-control	NR	NR	TB patients	Not clear—189 received INH	Peripheral neuropathy
Feng, 2014	China	Case-control	6 months	Treatment with anti-tuberculosis drug regimens at the usual dosage—300 mg/day INH, 450 mg/day RMP and 1500 mg/day PZA	Selection of cases: cases selected based on liver functions, i.e., all indices of liver function were normal before anti-tuberculosis chemotherapy, and became abnormal indicating hepatic injury after 6 months of chemotherapy. Cases were patients who showed ATDH based on increased serum transaminase values that were three-fold higher than the ULN (40 IU/l ALT) and symptoms compatible with hepatitis Selection of controls: controls underwent the same anti-tuberculosis chemotherapy with selected cases and were not tested for abnormality in liver functions after 6 months of the chemotherapy. The controls selected matched the criteria compared to the cases: 1) sex; 2) age discrepancy of <5 years; 3) living in the same regions; and 4) treatment with anti-tuberculosis drug regimens at the usual dosage, including 300 mg/day INH, 450 mg/day RMP and 1500 mg/day PZA	346	ATDH
Fredj, 2016	Tunisia	Prospective cohort	Serum AST, ALT and ALP were monitored monthly until the end of the treatment	The anti-tuberculosis treatment was based on the association of INH (5 mg/kg/day), RMP (10 mg/kg/day), PZA (25 mg/kg/day) and EMB (15 mg/kg/day) for the first 2 months, followed by INH and RMP for 4–7 additional months, depending on TB clinical presentation	Patients diagnosed with PTB and EPTB	71	INH-induced-hepatotoxicity
Gupta, 2013 (GI: GUPTA)	India	Prospective cohort	The patients were monitored for ALT, AST, andtotal bilirubin levels weekly for 1 month and then monthly until the completion of treatment	Initially patients received a combination regimen including INH 5 mg/kg (max 300 mg daily), RMP 10 mg/kg (max 600 mg daily), PZA 25 mg/kg (max 1500 mg daily) and EMB 15–25 mg/kg (max 2000 mg daily) for a period of 2 months, followed by an additional 4 months with INH and RMP	Inclusion criteria: 1) age > 18 years; 2) smear and/or culture positive for mycobacteria in clinical samples; and 3) normal ALT, AST and total bilirubin levels Exclusion criteria: 1) patients presenting clinically and laboratory-confirmed chronic liver disease such as jaundice; 2) acute and chronic hepatitis B and/or C or HIV; 3) alcoholic liver diseases; 4) a rise of two times the ULN of ALT, AST and total bilirubin levels; 5) medication with anti-tuberculosis drugs before start of treatment and/or other potentially hepatotoxic drugs; and 6) refusal to provide blood sample or signed informed consent form	215	ATDH
Higuchi, 2007 (GI: HIGUCHI)	Japan	Prospective cohort	NR	Treated with a INH (400 mg/d) and RMP (450 mg/d) containing regimen for 6 or 9 months	Patients with new onset of PTB treated with a INH (400 mg/d) and RMP (450 mg/d) containing regimen for 6 or 9 months Exclusion criteria: patients with liver cirrhosis, chronic and acute hepatitis, alcoholic liver disease and other chronic liver diseases	100	ATDH Skin rash Eosinophilia
Ho, 2013	Taiwan	Prospective cohort	180 days	All patients received oral INH 300 mg, RMP 600 mg (or 450 mg if body weight was <50 kg), PZA 25 mg/kg of body weight (max daily dose 2000 mg) and EMB 15 mg/kg of body weight daily (max daily dose 1600 mg) for the first 2 months. PZA was then discontinued, whereas INH, RMP and EMB were continued for another 4 months	The inclusion criteria included a sputum smear with AFB or culture positive for M. tuberculosis, undergoing anti-tuberculosis treatment, including INH or starting treatment during the recruitment period, hepatitis serology recorded in the medical chart with signed written consent Patients were excluded if they were infected with hepatitis B virus, hepatitis C virus or HIV, had any other hepatic disease, or did not have at least two visits documented in the medical records	348	ATDH
Huang, 2003 (GI: HUANG)	Taiwan	Prospective cohort	Serum ALT, AST and total bilirubin levels were monitored monthly until the end of treatment or checked whenever patients had symptoms of suspected hepatitis	Their standard daily anti-tuberculosis regimen for the first 2 months included INH (300 mg), RMP (600 mg or 450 mg if body weight <50 kg), PZA (20 mg/kg body weight) and EMB (25 mg/kg body weight). PZA was then discontinued, whereas INH, RMP, and EMB (15 mg/kg body weight) were continued for another 4 months	Patients with incident PTB or EPTB Exclusion criteria: 1) abnormal serum ALT, AST or bilirubin before anti-tuberculosis treatment; and 2) refusal of blood sampling or informed written consent	318	ADIH
Jung, 2015	Korea	Prospective cohort	4 weeks	Standard 4-drug treatment for 6 months: INH (5 mg/kg, usually 300 mg), RMP (450 mg for <50 kg or 600 mg for ⩾50 kg of body weight), EMB (15 mg/kg) and PZA (20–30 mg/kg), daily for 2 months, followed by INH and RMP with or without EMB for 4 months. Doses were adjusted when applying model-based treatment	Eligible participants were patients newly diagnosed with active TB, who underwent standard 4-drug treatment for 6 months Exclusion criteria: patients with abnormal hepatic function on laboratory testing (increased serum AST, ALT or total bilirubin) before anti-tuberculosis treatment and underlying liver disease or systemic illness such as congestive heart failure, acute life-threatening disease, or alcoholism or disease that was resistant to INH at the start of treatment	206	ATDH
Khalili, 2011	Iran	Case-control	2 months	Treated daily with INH (300 mg), RMP (600 mg), PZA (20 mg/kg), EMB (15 mg/kg) for the first 2 months, followed by INH and RMP daily for 4 additional months	Inclusion criteria: newly diagnosed patients (>18 years) with active PTB, who had been planned to be treated daily with anti-tuberculosis treatment (see drugs and dosage) Exclusion criteria: other possibilities of hepatitis, including patients with HIV infection, hepatic insufficiency (ALT or AST > 2× ULN) or clinically signs and symptoms of liver diseases such as jaundice and ascites, chronic hepatitis (B or C) and renal insufficiency (creatinine clearance < 50 ml/min based on Cockcroft-Gault equation) and history or current use of herbal, supplemental and hepatotoxic non-tuberculosis drugs	100	Hepatotoxicity
Kim, 2009 (GI: KIM)	Korea	Case-control	Assessments performed 2 weeks after onset of treatment and bi-monthly thereafter	All patients with PTB were treated daily with a combination regimen comprising INH (300–400 mg daily), RMP (450–600 mg daily), EMB (600–800 mg daily) and PZA (1000–1500 mg daily) for 2 months and then without PZA for ⩾4 following months. Doses of each drug were adjusted based on body weight of the patient	Newly diagnosed and treated patients with PTB. Exclusion criteria: patients with active or chronic hepatitis, including alcoholic hepatitis, fatty liver disease, liver cirrhosis, carriers of the hepatitis B or C virus, heavy alcohol intake, decreased renal function and severe cardiac diseases requiring several medications	226	ATDH
Kim, 2011 (GI: KIM)	Korea	Case-control	NR	The treatment comprised an initial phase of 2 months and a subsequent continuation phase of ⩾4 months. During the initial phase, 4 drugs were administered, including INH (300–400 mg daily), RMP (450–600 mg daily), EMB (600–800 mg daily) and PZA (1000–1500 mg daily). Doses of each drug were adjusted based on the body weight of the patient. In the following continuation phase, only PZA was discontinued, while the other three drugs were continued	Patients newly diagnosed with PTB and/or TB pleuritis and treated with first-line anti-tuberculosis medications such as INH, RMP, EMB and PZA Exclusion criteria: 1) patients with skin diseases before treatment, 2) chronic renal failure and chronic liver diseases affecting drug metabolism, 3) chronic alcoholism, 4) other chronic medical conditions requiring medication, and 5) non-adherence to treatment	221	ATD-induced MPE
Lee, 2010	Taiwan	Prospective cohort	NR	All patients received oral INH 300 mg, RMP 600 mg (or 450 mg, if body weight was <50 kg), PZA 200 mg/kg of body weight and EMB 800 mg daily for the first 2 months. PZA was then discontinued, while INH, RMP and EMB were continued for another 4 months	Inclusion criteria: adult patients newly diagnosed with active TB, having evident lesions of TB using simple X-ray, computed tomography, sputum smears and cultures positive for mycobacteria Exclusion criteria: 1) positive serum hepatitis B virus surface antigen, antibody to hepatitis C virus; 2) alcoholic liver disease or habitual alcohol drinking; 3) any other hepatic or systemic diseases that may cause liver dysfunction; 4) abnormal serum ALT, AST or bilirubin levels before anti-tuberculosis treatment	140	ATDH
Leiro-Fernandez, 2011	Spain	Case-control	Routine follow-up of clinical assessments every 2 weeks during the first month and monthly thereafter untilcompletion of treatment	Treatment with regimens that included INH, RMP and PZA at the usual dosages (INH 5 mg/kg/day to maximum 300 mg/day, RMP10 mg/kg/day to maximum 600 mg/day and PZA 25–30 mg/kg/day to maximum 2500 mg/day	Inclusion criteria: 1) age 15–75 years; 2) microbiological demonstration of active TB; 3) treatment with regimens that included INH, RMP and PZA at the usual dosages (INH 5 mg/kg/day to max 300 mg/day, RMP 10 mg/kg/day to max 600 mg/day and PZA 25–30 mg/kg/day to max 2500 mg/day); and 4) adequate treatment adherence Exclusion criteria: 1) increased baseline serum transaminases (AST and/or ALT; normal values ⩽ 40 IU/l); 2) positive serological testing for HIV, hepatitis B virus or hepatitis C virus; 3) regular alcohol intake or concomitant use of hepatotoxic drugs; 4) history of chronic liver disease; 5) pregnancy; or 6) no or poor adherence to treatment	117	ATDH
Lv, 2012	China	Case-control	6–9 months	All primary/retreatment patients took INH (600 mg), RMP (600 mg or 450 mg if body weight was <50 kg), PZA (2000 mg) and EMB (1250 mg) every other day in the first 2 months and then INH and RMP were continued for another 4/6 months. The retreatment patients received SM (750 mg) every other day in the first 2 months and continued receiving EMB for another 6 months	Inclusion criteria: sputum smear-positive PTB patients who received standard short-course chemotherapy recommended by the WHO Exclusion criteria: 1) positive serum hepatitis B virus surface antigen or other liver disease, 2) potentially hepatotoxic medications that would confound the picture, 3) abnormal serum ALT, AST or total bilirubin levels before anti-tuberculosis treatment	445	ATDH
Mahmoud, 2012	Tunisia	Prospective cohort	3 months	Treated with INH and RMP containing regimen	Inclusion criteria: adult patients (>18 years) newly diagnosed with TB treated with INH- and RMP-containing regimen, LFTs before initiation of treatment showed completely normal findings on serum ALT, AST, total bilirubin, ALP and gGTP Exclusion criteria: 1) patients receiving other potentially hepatotoxic drugs in addition to anti-tuberculosis agents; 2) positive serum hepatitis B surface antigen or hepatitis C antibody; 3) alcoholic liver disease; 4) any other hepatic or systemic diseases that may cause liver dysfunction	66	INH hepatotoxicity
Ng, 2014	Cases recruited in the UK; controls were samples from elsewhere	Case-control	NR	All had been prescribed INH with all but one also taking additional anti-tuberculosis drugs. Patients were prescribed 300 mg INH/day in line with UK/WHO guidelines	Cases: patients diagnosed with DILI either in the past or at the time of sample collection. Onlycases assessed as having DILI that was highly probably, probably or possibly relating to anti-tuberculosis drug exposure were enrolled in the study. Other possible causes of liver toxicity, particularly hepatitis A, B, C and CMV infection were excluded. No patients included were HIV-positive or had pre-existing liver disease. None had been treated for TB under directly observed treatment Controls: 52 DNA samples from individuals of European ancestry; inclusion/exclusion criteria not reported	127	DILI
Ohno, 2000	Japan	Prospective cohort	3 months	Initial chemotherapy always included INH (400 mg/day; 8.2 ± 2.0 mg/kg/day) and RMP (450 mg/day; 9.2 ± 2.2 mg/kg/day); the third drug used was EMB or SM.	Inclusion criteria: patients had no history of alcohol abuse and were negative for hepatitis B surface antigen and hepatitis C antibody. At the beginning of treatment, LFTs showed completely normal findings on serum AST, ALT, bilirubin, ALP and gGTP Patients receiving other potentially hepatopathic drugs in addition to anti-tuberculosis agents were excluded from the study	77	INH + RMP-induced hepatotoxicity
Possuelo, 2008 (GI: POSSUELO)	Brazil	Prospective cohort	NR	Treatment daily with INH, RMP and PZA for the first 2 months followed by INH and RMP daily for 4 additional months. Drug dosages used were calculated according to patient's weight (weight <45 kg: RMP 300 mg, INH 200 mg, PZA 1000 mg; 45–55 kg: RMP 450 mg, INH 300 mg, PZA 1500 mg; >55 kg: RMP 600 mg, INH 400 mg, PZA 2000 mg)	Inclusion criteria: adult patients (>18 years) newly diagnosed with active TB, who had been treated daily with anti-tuberculosis treatment (see drugs and dosage) Exclusion criteria: patients presenting clinically and laboratory-confirmed liver chronic disease, patients using anti-tuberculosis drugs before enrolment in the study, patients presenting results of LFTs before the beginning of treatment higher that were 2× ULN and refusal to participate of the study	254	ATDH Gastrointestinal ADRs
Rana, 2014 (GI: RANA)	India	Prospective cohort	Patients were monitored every month until the end of treatment or whenever the patients had symptoms or signs of hepatotoxicity	Daily anti-tuberculosis treatment for the first 2 months included INH (300 mg), RMP (600 or 450 mg for body weight/50 kg), PZA (20 mg/kg body weight) and EMB (25 mg/kg body weight). After 2 months, EMB and PZA were discontinued, whereas INH and RMP were continued for an additional 4 months	Inclusion criteria: patients with pulmonary and EPTB Exclusion criteria: patients with history of alcohol abuse and/or any other liver disease; patients who had received other potentially hepatotoxic drugs in addition to anti-tuberculosis drugs; patients who had abnormal serum ALT, AST or bilirubin before starting anti-tuberculosis treatment; patientswho developed viral hepatitis during anti-tuberculosis treatment; patients with renal failure or cancer; and patients who refused blood sampling or providing informed written consent	300	Hepatotoxicity
Santos, 2013 (GI: SANTOS)	Brazil	Prospective cohort	NR	Treatment with INH, RMP and PZA for the first 2 months, followed by INH and RMP daily for 4 months	Inclusion criteria: patients diagnosed with TB and treated with anti-tuberculosis treatment (see drugs and dosage) Exclusion criteria: patients aged <18 years, those with mental disabilities, chronic liver disease confirmed by clinical and laboratory data, users of anti-tuberculosis drugs before enrolment in the study, and those with liver function results > 2× ULN before beginning treatment	270	ATDH
Shimizu, 2006	Japan	Prospective cohort	3 months	Treatment with INH (300–400 mg) and RMP (300–450 mg)	Individuals with PTB without abnormal serum levels of ALT, AST, direct or total bilirubin, or serum hepatitis B virus surface antigen or antibody to hepatitis C virus at the beginning of the study	42	Hepatotoxicity
Singla, 2014	India	Prospective cohort	NR	NR	Inclusion criteria: newly diagnosed patients with TB Exclusion criteria: history of heavy use of alcohol or chronic liver diseases or liver cirrhosis;patients who were settled in the area for a minimum of three generations; people infected with HIV	408	ATDH
Sotsuka, 2011	Japan	Prospective cohort	3 months	INH, RMP and PZA, plus EMB or SM during the first 2 months, followed by administration of INH and RMP plus EMB or SM during the final 4 months	Inclusion criteria: in-patients with active PTB who were treated with the standard Japanese chemotherapy regimen, followed up for >3 months after treatment, and who consented to this study	144	Hepatotoxicity
Teixeira, 2011	Brazil	Case-control	NR	Anti-tuberculosis drug regimens that include INH at the usual dosage (400 mg/day)	Inclusion criteria: 1) age > 18 years, 2) diagnosis of active TB, 3) treatment with anti-tuberculosis drug regimens that include INH at the usual dosage (400 mg/day) and 4) normal baseline serum transaminases (ALT and AST) before treatment Exclusion criteria: 1) positive serological test for the HIV, hepatitis B virus or hepatitis C virus, 2) alcohol abuse, 3) history of chronic liver disease and 4) pregnancy	167	Hepatitis
Vuilleumier, 2006	Switzerland	Prospective cohort	NR	INH 300 mg daily and vitamin B6 40 mg per day for a period of 6 months	Inclusion criteria: patients having LTBI as defined by the American Thoracic Society with normal plasma AST and ALT levels before the beginning of INH monotherapy Exclusion criteria: 1) a history of alcohol consumption, 2) positive serology for the HAV, HBV or HCV, 3) poor chemotherapy compliance (negative urine INH 3 times during the follow-up), loss during the follow-up, 4) patients had to receive supplementary anti-tuberculosis agents or other potentially hepatotoxic drugs	89	INH-induced hepatotoxicity
Wang, 2015 (GI: NTUH)	Taiwan	Prospective cohort	6 months	Daily INH, RMP, EMB and PZA in the first 2 months, and daily INH and RMP for the next 4 months The daily dosage of each drug was calculated by weight	Adult Taiwanese (>16 years) patients with culture-confirmed PTB Subjects were excluded if they were pregnant, had a life expectancy < 6 months, had abnormal baseline LFT or were resistant to INH, RMP or both	355 in the derivation cohort, 182 in the validation cohort	Hepatotoxicity during anti-tuberculosis treatment
Xiang, 2014	China	Prospective cohort	2 months	All patients were prescribed INH (600 mg), RMP (600 mg or 450 mg if the body weight was <50 kg), PZA (2000 mg) and EMB (1250 mg) every other day in the first 2 months. After 2 months, INH and RMP were continued for a further 4–6 months. Retreatment patients in addition received SM (750 mg) every other day in the first 2 months and continued receiving EMB for another 6 months	Inclusion criteria: newly diagnosed PTB patients belonging to the Uyghur ethnic group, who were receiving standard short-course chemotherapy recommended by the WHO, who attended for a 2-month assessment, and any patients attending clinic with suspected liver disease after the start of treatment, before the 2-month visit Exclusion criteria: patients who had signs of abnormal liver function when they started treatment (jaundice or elevated ALT, AST or bilirubin levels) or disease associated with liver dysfunction	2244	ATLI
Yamada, 2009 (GI: YAMADA)	Canada	Prospective cohort	9 months	INH 300 mg	Subjects who were receiving treatment with INH 300 mg daily self-administered for LTBI.Subjects were included if they were aged ⩾19 years; not concurrently receiving other anti-tuberculosis drugs; non-reactive to hepatitis B surface antigen and antibody to hepatitis C virus; absence of any liver or metabolic diseases; without a HIV-positive test result; not consuming ⩾7 alcoholic beverages per day and undergoing sufficiently frequentAST tests to detect hepatotoxicity	170	Hepatotoxicity
Yimer, 2011	Ethiopia	Prospective cohort	followed up for development of DILI for up to 56 weeks	All study participants received RMP based short-course chemotherapy for TB following the national TB treatment guideline. ART was then initiated	Newly diagnosed ART and anti-tuberculosis treatment-naïve adult TB and HIV co-infected patients. The eligibility criteria were age >18 years, CD4 count, 200 cells/Ul, not pregnant and not on other known hepatotoxic drugs concurrently (except cotrimoxazole, 960 mg per day, which was given to all participants before enrolment and during the follow-up period according to the treatment guideline)	353	Anti-tubercular and antiretroviral DILI
Yuliwulandari, 2016	Indonesia	Case-control	NR	NR (anti-tuberculosis treatment)	Patients included in the study were male and female aged 15–70 years, suffering from TB according to the WHO standards, and were under monitored treatment with anti-tuberculosis drugs Exclusion criteria: 1) history of liver disease such as: hepatitis A B or C; hepatoma; liver cirrhosis or cholelithiasis positive; 2) abnormal levels of any LFT (ALT, AST or total bilirubin) before anti-tuberculosis treatment	241	ATLI
Zaverucha-do-Valle, 2014	Brazil	Retrospective cohort	Follow-up at days 15 and 30 and at least one visit monthly until the end of treatment	600 mg/day of RMP, 400 mg/day of INH and 2 g/day of PZA for all patients with corporal weight >45 kg or adjusted for corporal weight <45 kg. After 2 months of treatment, PZA was discontinued	Inclusion criteria: signed written consent, sputum smear with AFB or culture positive for M. tuberculosis; ongoing TB treatment and laboratory tests of liver function Exclusion criteria: age <18 years, pregnancy and no >1 visit registered	131	ATDH

INH =isoniazid; RMP =rifampicin; PZA =pyrazinamide; EMB =ethambutol; ALT =alanine aminotransferase; AST =aspartate aminotransferase; RCT =randomised controlled trial; HIV =human immunodeficiency virus; SM =streptomycin; PTB =pulmonary tuberculosis; IU =international unit; ALP =alkaline phosphatase; DILI =drug-induced liver injury; LFT =liver function test; DIH =drug-induced hepatotoxicity; NR =not reported; ATDH =anti-tuberculosis drug-induced hepatotoxicity; GI =group identifier; ADR =adverse drug reaction; ULN =upper limit of normal; EPTB =extra-pulmonary TB; ADIH =anti-tuberculosis drug-induced hepatitis; ATD =anti-tuberculosis drug; MPE =maculopapular eruption; WHO = World Health Organization; gGTP = gamma-glutamyltranspeptidase; CMV = cytomegalovirus ; LTBI = latent tuberculous infection; ART =antiretroviral therapy; ATLI = anti-tuberculosis drug-induced liver injury.

**Table A.3:** (*continued*)

Author, year	Country	Study design	Follow-up time	Drugs and dosage	Selection criteria	Sample size *n*	Toxicity outcomes
An, 2012	China	App	6 months	Daily treatment with INH, RMP, PZA and EMB for 2 months, followed by 4 months treatment with INH and RMP, with drug dosages calculated according to body weight Body weight < 45 kg: RMP 300 mg, INH 200 mg, PZA 1000 mg Body weight of 45–55 kg: RMP 450 mg, INH 300 mg, PZA 1500 mg Body weight > 55 kg: RMP 600 mg, INH 400 mg, PZA 2000 mg	Inclusion criteria: Normal serum ALT, AST and bilirubin levels, no symptoms related to abnormal liver function (i.e., jaundice) before anti-tuberculosis drug treatment and close monitoring of changes in liver functions within 6 months of treatment Patients with and without hepatotoxicity during treatment Exclusion criteria: Malnutrition HIV type 1 infection Alcoholic liver disease or habitual drinking Hepatitis B or C infection, liver disease, systemic diseases and/or treatment with drugs other than the anti-tuberculosis drugs that can induce hepatotoxicity Severe TB or cardiac dysfunction that may cause liver dysfunction Transient increases in ALT	208	Hepatotoxocity
Azuma, 2013	Japan	RCT	6 months	All patients were treated with a 6-month regimen comprising INH, RMP, PZA and EMB/SM for the first 2 months, followed by RMP and INH for 4 months. All patients were started on the standard oral dose (~5 mg/kg body weight, once-daily). For pharmacogenetics-treatment patients, dosages were adjusted based on individual *NAT2* status within 3 days. Modified daily INH doses were respectively ~7.5, ~5 and ~2.5 mg/kg for rapid, intermediate and slow acetylators. Regarding the other drugs for the standard regimen, standard daily doses of RMP (10 mg/kg, maximum 600 mg/body), PZA (25 mg/kg, 1500 mg/body), EMB (15 mg/kg, 750 mg/body; 20 mg/kg, 1000 mg/body) and SM (15 mg/kg, 750 mg/body) were recommended with the following dose ranges allowed at the discretion of the physician in charge: RMP 8–12 mg/kg; PZA 20–30 mg/kg; EMB 15–20 mg/kg; SM 12–18 mg/kg	The eligible population was identified from a series of patients newly diagnosed with PTB (male and female aged 20–75 years), requiring the 6-month 4-drug standard treatment for the first time Exclusion criteria: abnormal test results for liver and kidney function (serum AST > 45 IU/l, ALT > 50 IU/l, ALP > 444 IU/l, total bilirubin > 1.6 mg/dl and creatinine > 1.4 mg/dl before the study treatments commenced, long-term use of steroids and/or immunosuppressants, inadequate clinical conditions such as hyperglycaemia, diabetes mellitus, acute life-threatening chronic progressive disease, pregnancy or lactation and alcoholism. Patients not expected to complete the study protocol for social reasons were not recruited	172	INH-DILI Peripheral neuropathy
Bose, 2011	India	Prospective cohort	Patients were followed up to 8 weeks after they had started with anti-tuberculosis treatment	All patients received anti-tuberculosis treatment (RMP, INH, EMB and PZA) according to body weight. All four drugs were given for 2 months. PZA and EMB were discontinued, while INH and RMP were continued for another 4 months RMP doses as follows: ⩽35 kg: 300 mg/day 36–50 kg: 450 mg/day >50 kg: 600 mg/day INH doses as follows: ⩽35 kg: 200 mg/day >35 kg: 400 mg/day PZA doses as follows: ⩽50 kg: 1.0 g/day >50 kg: 1.5 g/day EMB 20 mg/kg/day	Newly diagnosed patients of PTB. The baseline LFT of the patients was normal when they were started on anti-tuberculosis treatment Exclusion criteria: no history of habitual alcoholism, chronic liver diseases or steatosis	218	ADIH ADIH outcome
Çetintaş, 2008	Turkey	Prospective cohort	NR	Patients received INH 5 mg/kg (maximum 300 mg/day), RMP 10 mg/kg (maximum 600 mg/day), PZA 25 mg/kg (maximum 2000 mg/day), EMB 15–25 mg/kg (maximum 1500 mg/day)	Patients diagnosed with TB. Only patients with serum levels before initiation of treatment within the following ranges were included in the study: ALT 0–40 U/l, AST 5–45 U/l and total bilirubin 0.2–1.6 mg/dl Exclusion criteria: patients with positive serum hepatitis B surface antigen, hepatitis C antibody or hepatitis A immunoglobulin M antibody, and patients with alcoholic liver disease or any hepatic or systemic disease that could cause liver function disorder	100	DIH
Chamorro, 2013	Argentina	Prospective cohort	NR	The patients began a standard anti-tuberculosis treatment protocol for the first 2 months (INH 5 mg/kg/days, maximum 300 mg/day, RMP 10 mg/kg/day maximum 600 mg/day, PZA 20 mg/kg/day, EMB 20 mg/kg/day), followed by INH and RMP for ⩾4 months, depending on the disease severity or the presence ofextra-pulmonary foci	Inclusion criteria: TB patients aged >18 years with stable haemodynamic levels, normal renal function and negative for pregnancy Exclusion criteria: presence of diseases that directly affected the liver (acute hepatitis A, active hepatitis B and C; cirrhosis; encephalopathy and cancer), INH allergy, HIV, autoimmunity, concomitant hepatotoxic medications, a history of TB treatment failures, refusal of blood extraction and refusal to sign the written informed consent	175	ATDH
Chang, 2012	Taiwan	Prospective cohort	NR	First-line anti-tuberculosis medications	Patients diagnosed with TB and treated with first-line anti-tuberculosis medications Exclusion criteria: pregnancy, abnormal liver function and history of positive viral hepatitis before starting treatment with first-line anti-tuberculosis drugs	98	ATDH
Cho, 2007	Korea	Prospective cohort	Serum AST, ALT and total bilirubin levels were then monitored monthly until the end of treatment	All patients received oral INH (300 mg), RMP (600 mg), PZA (20 mg/kg body weight) and EMB (800 mg) daily for the first 2 months. PZA was then discontinued, while INH, RMP and EMB were continued for another 4 months	Adult patients newly diagnosed with active TB, with evident lesion of TB using simple X-ray or computed tomography or positive results on sputum smear or culture for detection of mycobacteria Exclusion criteria: 1) abnormal serum ALT, AST, or bilirubin levels or symptoms related to abnormal liver function such as jaundice before anti-tuberculosis treatment; 2) alcoholic liver disease or habitual alcohol drinking; 3) any other hepatic or systemic diseases that may cause liver dysfunction	132	ATDH
Costa, 2012	Brazil	Prospective cohort	NR	All patients were treated with the first-line anti-tuberculosis drug regimen INH (300 mg/kg/day), RMP (300 mg/kg/day) and PZA (1500 mg/kg/day) for the first 2 months, followed by INH and RMP for a further 4 months	Male or female subjects aged ⩾18 years, with no previously described renal, allergic or hepatic diseases and not pregnant	129	ADRs
Dhoro, 2013	Zimbabwe	Case-control	NR	NR	TB patients	Not clear—189 received INH	Peripheral neuropathy
Feng, 2014	China	Case-control	6 months	Treatment with anti-tuberculosis drug regimens at the usual dosage—300 mg/day INH, 450 mg/day RMP and 1500 mg/day PZA	Selection of cases: cases selected based on liver functions, i.e., all indices of liver function were normal before anti-tuberculosis chemotherapy, and became abnormal indicating hepatic injury after 6 months of chemotherapy. Cases were patients who showed ATDH based on increased serum transaminase values that were three-fold higher than the ULN (40 IU/l ALT) and symptoms compatible with hepatitis Selection of controls: controls underwent the same anti-tuberculosis chemotherapy with selected cases and were not tested for abnormality in liver functions after 6 months of the chemotherapy. The controls selected matched the criteria compared to the cases: 1) sex; 2) age discrepancy of <5 years; 3) living in the same regions; and 4) treatment with anti-tuberculosis drug regimens at the usual dosage, including 300 mg/day INH, 450 mg/day RMP and 1500 mg/day PZA	346	ATDH
Fredj, 2016	Tunisia	Prospective cohort	Serum AST, ALT and ALP were monitored monthly until the end of the treatment	The anti-tuberculosis treatment was based on the association of INH (5 mg/kg/day), RMP (10 mg/kg/day), PZA (25 mg/kg/day) and EMB (15 mg/kg/day) for the first 2 months, followed by INH and RMP for 4–7 additional months, depending on TB clinical presentation	Patients diagnosed with PTB and EPTB	71	INH-induced-hepatotoxicity
Gupta, 2013 (GI: GUPTA)	India	Prospective cohort	The patients were monitored for ALT, AST, andtotal bilirubin levels weekly for 1 month and then monthly until the completion of treatment	Initially patients received a combination regimen including INH 5 mg/kg (max 300 mg daily), RMP 10 mg/kg (max 600 mg daily), PZA 25 mg/kg (max 1500 mg daily) and EMB 15–25 mg/kg (max 2000 mg daily) for a period of 2 months, followed by an additional 4 months with INH and RMP	Inclusion criteria: 1) age > 18 years; 2) smear and/or culture positive for mycobacteria in clinical samples; and 3) normal ALT, AST and total bilirubin levels Exclusion criteria: 1) patients presenting clinically and laboratory-confirmed chronic liver disease such as jaundice; 2) acute and chronic hepatitis B and/or C or HIV; 3) alcoholic liver diseases; 4) a rise of two times the ULN of ALT, AST and total bilirubin levels; 5) medication with anti-tuberculosis drugs before start of treatment and/or other potentially hepatotoxic drugs; and 6) refusal to provide blood sample or signed informed consent form	215	ATDH
Higuchi, 2007 (GI: HIGUCHI)	Japan	Prospective cohort	NR	Treated with a INH (400 mg/d) and RMP (450 mg/d) containing regimen for 6 or 9 months	Patients with new onset of PTB treated with a INH (400 mg/d) and RMP (450 mg/d) containing regimen for 6 or 9 months Exclusion criteria: patients with liver cirrhosis, chronic and acute hepatitis, alcoholic liver disease and other chronic liver diseases	100	ATDH Skin rash Eosinophilia
Ho, 2013	Taiwan	Prospective cohort	180 days	All patients received oral INH 300 mg, RMP 600 mg (or 450 mg if body weight was <50 kg), PZA 25 mg/kg of body weight (max daily dose 2000 mg) and EMB 15 mg/kg of body weight daily (max daily dose 1600 mg) for the first 2 months. PZA was then discontinued, whereas INH, RMP and EMB were continued for another 4 months	The inclusion criteria included a sputum smear with AFB or culture positive for M. tuberculosis, undergoing anti-tuberculosis treatment, including INH or starting treatment during the recruitment period, hepatitis serology recorded in the medical chart with signed written consent Patients were excluded if they were infected with hepatitis B virus, hepatitis C virus or HIV, had any other hepatic disease, or did not have at least two visits documented in the medical records	348	ATDH
Huang, 2003 (GI: HUANG)	Taiwan	Prospective cohort	Serum ALT, AST and total bilirubin levels were monitored monthly until the end of treatment or checked whenever patients had symptoms of suspected hepatitis	Their standard daily anti-tuberculosis regimen for the first 2 months included INH (300 mg), RMP (600 mg or 450 mg if body weight <50 kg), PZA (20 mg/kg body weight) and EMB (25 mg/kg body weight). PZA was then discontinued, whereas INH, RMP, and EMB (15 mg/kg body weight) were continued for another 4 months	Patients with incident PTB or EPTB Exclusion criteria: 1) abnormal serum ALT, AST or bilirubin before anti-tuberculosis treatment; and 2) refusal of blood sampling or informed written consent	318	ADIH
Jung, 2015	Korea	Prospective cohort	4 weeks	Standard 4-drug treatment for 6 months: INH (5 mg/kg, usually 300 mg), RMP (450 mg for <50 kg or 600 mg for ⩾50 kg of body weight), EMB (15 mg/kg) and PZA (20–30 mg/kg), daily for 2 months, followed by INH and RMP with or without EMB for 4 months. Doses were adjusted when applying model-based treatment	Eligible participants were patients newly diagnosed with active TB, who underwent standard 4-drug treatment for 6 months Exclusion criteria: patients with abnormal hepatic function on laboratory testing (increased serum AST, ALT or total bilirubin) before anti-tuberculosis treatment and underlying liver disease or systemic illness such as congestive heart failure, acute life-threatening disease, or alcoholism or disease that was resistant to INH at the start of treatment	206	ATDH
Khalili, 2011	Iran	Case-control	2 months	Treated daily with INH (300 mg), RMP (600 mg), PZA (20 mg/kg), EMB (15 mg/kg) for the first 2 months, followed by INH and RMP daily for 4 additional months	Inclusion criteria: newly diagnosed patients (>18 years) with active PTB, who had been planned to be treated daily with anti-tuberculosis treatment (see drugs and dosage) Exclusion criteria: other possibilities of hepatitis, including patients with HIV infection, hepatic insufficiency (ALT or AST > 2× ULN) or clinically signs and symptoms of liver diseases such as jaundice and ascites, chronic hepatitis (B or C) and renal insufficiency (creatinine clearance < 50 ml/min based on Cockcroft-Gault equation) and history or current use of herbal, supplemental and hepatotoxic non-tuberculosis drugs	100	Hepatotoxicity
Kim, 2009 (GI: KIM)	Korea	Case-control	Assessments performed 2 weeks after onset of treatment and bi-monthly thereafter	All patients with PTB were treated daily with a combination regimen comprising INH (300–400 mg daily), RMP (450–600 mg daily), EMB (600–800 mg daily) and PZA (1000–1500 mg daily) for 2 months and then without PZA for ⩾4 following months. Doses of each drug were adjusted based on body weight of the patient	Newly diagnosed and treated patients with PTB. Exclusion criteria: patients with active or chronic hepatitis, including alcoholic hepatitis, fatty liver disease, liver cirrhosis, carriers of the hepatitis B or C virus, heavy alcohol intake, decreased renal function and severe cardiac diseases requiring several medications	226	ATDH
Kim, 2011 (GI: KIM)	Korea	Case-control	NR	The treatment comprised an initial phase of 2 months and a subsequent continuation phase of ⩾4 months. During the initial phase, 4 drugs were administered, including INH (300–400 mg daily), RMP (450–600 mg daily), EMB (600–800 mg daily) and PZA (1000–1500 mg daily). Doses of each drug were adjusted based on the body weight of the patient. In the following continuation phase, only PZA was discontinued, while the other three drugs were continued	Patients newly diagnosed with PTB and/or TB pleuritis and treated with first-line anti-tuberculosis medications such as INH, RMP, EMB and PZA Exclusion criteria: 1) patients with skin diseases before treatment, 2) chronic renal failure and chronic liver diseases affecting drug metabolism, 3) chronic alcoholism, 4) other chronic medical conditions requiring medication, and 5) non-adherence to treatment	221	ATD-induced MPE
Lee, 2010	Taiwan	Prospective cohort	NR	All patients received oral INH 300 mg, RMP 600 mg (or 450 mg, if body weight was <50 kg), PZA 200 mg/kg of body weight and EMB 800 mg daily for the first 2 months. PZA was then discontinued, while INH, RMP and EMB were continued for another 4 months	Inclusion criteria: adult patients newly diagnosed with active TB, having evident lesions of TB using simple X-ray, computed tomography, sputum smears and cultures positive for mycobacteria Exclusion criteria: 1) positive serum hepatitis B virus surface antigen, antibody to hepatitis C virus; 2) alcoholic liver disease or habitual alcohol drinking; 3) any other hepatic or systemic diseases that may cause liver dysfunction; 4) abnormal serum ALT, AST or bilirubin levels before anti-tuberculosis treatment	140	ATDH
Leiro-Fernandez, 2011	Spain	Case-control	Routine follow-up of clinical assessments every 2 weeks during the first month and monthly thereafter untilcompletion of treatment	Treatment with regimens that included INH, RMP and PZA at the usual dosages (INH 5 mg/kg/day to maximum 300 mg/day, RMP10 mg/kg/day to maximum 600 mg/day and PZA 25–30 mg/kg/day to maximum 2500 mg/day	Inclusion criteria: 1) age 15–75 years; 2) microbiological demonstration of active TB; 3) treatment with regimens that included INH, RMP and PZA at the usual dosages (INH 5 mg/kg/day to max 300 mg/day, RMP 10 mg/kg/day to max 600 mg/day and PZA 25–30 mg/kg/day to max 2500 mg/day); and 4) adequate treatment adherence Exclusion criteria: 1) increased baseline serum transaminases (AST and/or ALT; normal values ⩽ 40 IU/l); 2) positive serological testing for HIV, hepatitis B virus or hepatitis C virus; 3) regular alcohol intake or concomitant use of hepatotoxic drugs; 4) history of chronic liver disease; 5) pregnancy; or 6) no or poor adherence to treatment	117	ATDH
Lv, 2012	China	Case-control	6–9 months	All primary/retreatment patients took INH (600 mg), RMP (600 mg or 450 mg if body weight was <50 kg), PZA (2000 mg) and EMB (1250 mg) every other day in the first 2 months and then INH and RMP were continued for another 4/6 months. The retreatment patients received SM (750 mg) every other day in the first 2 months and continued receiving EMB for another 6 months	Inclusion criteria: sputum smear-positive PTB patients who received standard short-course chemotherapy recommended by the WHO Exclusion criteria: 1) positive serum hepatitis B virus surface antigen or other liver disease, 2) potentially hepatotoxic medications that would confound the picture, 3) abnormal serum ALT, AST or total bilirubin levels before anti-tuberculosis treatment	445	ATDH
Mahmoud, 2012	Tunisia	Prospective cohort	3 months	Treated with INH and RMP containing regimen	Inclusion criteria: adult patients (>18 years) newly diagnosed with TB treated with INH- and RMP-containing regimen, LFTs before initiation of treatment showed completely normal findings on serum ALT, AST, total bilirubin, ALP and gGTP Exclusion criteria: 1) patients receiving other potentially hepatotoxic drugs in addition to anti-tuberculosis agents; 2) positive serum hepatitis B surface antigen or hepatitis C antibody; 3) alcoholic liver disease; 4) any other hepatic or systemic diseases that may cause liver dysfunction	66	INH hepatotoxicity
Ng, 2014	Cases recruited in the UK; controls were samples from elsewhere	Case-control	NR	All had been prescribed INH with all but one also taking additional anti-tuberculosis drugs. Patients were prescribed 300 mg INH/day in line with UK/WHO guidelines	Cases: patients diagnosed with DILI either in the past or at the time of sample collection. Onlycases assessed as having DILI that was highly probably, probably or possibly relating to anti-tuberculosis drug exposure were enrolled in the study. Other possible causes of liver toxicity, particularly hepatitis A, B, C and CMV infection were excluded. No patients included were HIV-positive or had pre-existing liver disease. None had been treated for TB under directly observed treatment Controls: 52 DNA samples from individuals of European ancestry; inclusion/exclusion criteria not reported	127	DILI
Ohno, 2000	Japan	Prospective cohort	3 months	Initial chemotherapy always included INH (400 mg/day; 8.2 ± 2.0 mg/kg/day) and RMP (450 mg/day; 9.2 ± 2.2 mg/kg/day); the third drug used was EMB or SM.	Inclusion criteria: patients had no history of alcohol abuse and were negative for hepatitis B surface antigen and hepatitis C antibody. At the beginning of treatment, LFTs showed completely normal findings on serum AST, ALT, bilirubin, ALP and gGTP Patients receiving other potentially hepatopathic drugs in addition to anti-tuberculosis agents were excluded from the study	77	INH + RMP-induced hepatotoxicity
Possuelo, 2008 (GI: POSSUELO)	Brazil	Prospective cohort	NR	Treatment daily with INH, RMP and PZA for the first 2 months followed by INH and RMP daily for 4 additional months. Drug dosages used were calculated according to patient's weight (weight <45 kg: RMP 300 mg, INH 200 mg, PZA 1000 mg; 45–55 kg: RMP 450 mg, INH 300 mg, PZA 1500 mg; >55 kg: RMP 600 mg, INH 400 mg, PZA 2000 mg)	Inclusion criteria: adult patients (>18 years) newly diagnosed with active TB, who had been treated daily with anti-tuberculosis treatment (see drugs and dosage) Exclusion criteria: patients presenting clinically and laboratory-confirmed liver chronic disease, patients using anti-tuberculosis drugs before enrolment in the study, patients presenting results of LFTs before the beginning of treatment higher that were 2× ULN and refusal to participate of the study	254	ATDH Gastrointestinal ADRs
Rana, 2014 (GI: RANA)	India	Prospective cohort	Patients were monitored every month until the end of treatment or whenever the patients had symptoms or signs of hepatotoxicity	Daily anti-tuberculosis treatment for the first 2 months included INH (300 mg), RMP (600 or 450 mg for body weight/50 kg), PZA (20 mg/kg body weight) and EMB (25 mg/kg body weight). After 2 months, EMB and PZA were discontinued, whereas INH and RMP were continued for an additional 4 months	Inclusion criteria: patients with pulmonary and EPTB Exclusion criteria: patients with history of alcohol abuse and/or any other liver disease; patients who had received other potentially hepatotoxic drugs in addition to anti-tuberculosis drugs; patients who had abnormal serum ALT, AST or bilirubin before starting anti-tuberculosis treatment; patientswho developed viral hepatitis during anti-tuberculosis treatment; patients with renal failure or cancer; and patients who refused blood sampling or providing informed written consent	300	Hepatotoxicity
Santos, 2013 (GI: SANTOS)	Brazil	Prospective cohort	NR	Treatment with INH, RMP and PZA for the first 2 months, followed by INH and RMP daily for 4 months	Inclusion criteria: patients diagnosed with TB and treated with anti-tuberculosis treatment (see drugs and dosage) Exclusion criteria: patients aged <18 years, those with mental disabilities, chronic liver disease confirmed by clinical and laboratory data, users of anti-tuberculosis drugs before enrolment in the study, and those with liver function results > 2× ULN before beginning treatment	270	ATDH
Shimizu, 2006	Japan	Prospective cohort	3 months	Treatment with INH (300–400 mg) and RMP (300–450 mg)	Individuals with PTB without abnormal serum levels of ALT, AST, direct or total bilirubin, or serum hepatitis B virus surface antigen or antibody to hepatitis C virus at the beginning of the study	42	Hepatotoxicity
Singla, 2014	India	Prospective cohort	NR	NR	Inclusion criteria: newly diagnosed patients with TB Exclusion criteria: history of heavy use of alcohol or chronic liver diseases or liver cirrhosis;patients who were settled in the area for a minimum of three generations; people infected with HIV	408	ATDH
Sotsuka, 2011	Japan	Prospective cohort	3 months	INH, RMP and PZA, plus EMB or SM during the first 2 months, followed by administration of INH and RMP plus EMB or SM during the final 4 months	Inclusion criteria: in-patients with active PTB who were treated with the standard Japanese chemotherapy regimen, followed up for >3 months after treatment, and who consented to this study	144	Hepatotoxicity
Teixeira, 2011	Brazil	Case-control	NR	Anti-tuberculosis drug regimens that include INH at the usual dosage (400 mg/day)	Inclusion criteria: 1) age > 18 years, 2) diagnosis of active TB, 3) treatment with anti-tuberculosis drug regimens that include INH at the usual dosage (400 mg/day) and 4) normal baseline serum transaminases (ALT and AST) before treatment Exclusion criteria: 1) positive serological test for the HIV, hepatitis B virus or hepatitis C virus, 2) alcohol abuse, 3) history of chronic liver disease and 4) pregnancy	167	Hepatitis
Vuilleumier, 2006	Switzerland	Prospective cohort	NR	INH 300 mg daily and vitamin B6 40 mg per day for a period of 6 months	Inclusion criteria: patients having LTBI as defined by the American Thoracic Society with normal plasma AST and ALT levels before the beginning of INH monotherapy Exclusion criteria: 1) a history of alcohol consumption, 2) positive serology for the HAV, HBV or HCV, 3) poor chemotherapy compliance (negative urine INH 3 times during the follow-up), loss during the follow-up, 4) patients had to receive supplementary anti-tuberculosis agents or other potentially hepatotoxic drugs	89	INH-induced hepatotoxicity
Wang, 2015 (GI: NTUH)	Taiwan	Prospective cohort	6 months	Daily INH, RMP, EMB and PZA in the first 2 months, and daily INH and RMP for the next 4 months The daily dosage of each drug was calculated by weight	Adult Taiwanese (>16 years) patients with culture-confirmed PTB Subjects were excluded if they were pregnant, had a life expectancy < 6 months, had abnormal baseline LFT or were resistant to INH, RMP or both	355 in the derivation cohort, 182 in the validation cohort	Hepatotoxicity during anti-tuberculosis treatment
Xiang, 2014	China	Prospective cohort	2 months	All patients were prescribed INH (600 mg), RMP (600 mg or 450 mg if the body weight was <50 kg), PZA (2000 mg) and EMB (1250 mg) every other day in the first 2 months. After 2 months, INH and RMP were continued for a further 4–6 months. Retreatment patients in addition received SM (750 mg) every other day in the first 2 months and continued receiving EMB for another 6 months	Inclusion criteria: newly diagnosed PTB patients belonging to the Uyghur ethnic group, who were receiving standard short-course chemotherapy recommended by the WHO, who attended for a 2-month assessment, and any patients attending clinic with suspected liver disease after the start of treatment, before the 2-month visit Exclusion criteria: patients who had signs of abnormal liver function when they started treatment (jaundice or elevated ALT, AST or bilirubin levels) or disease associated with liver dysfunction	2244	ATLI
Yamada, 2009 (GI: YAMADA)	Canada	Prospective cohort	9 months	INH 300 mg	Subjects who were receiving treatment with INH 300 mg daily self-administered for LTBI.Subjects were included if they were aged ⩾19 years; not concurrently receiving other anti-tuberculosis drugs; non-reactive to hepatitis B surface antigen and antibody to hepatitis C virus; absence of any liver or metabolic diseases; without a HIV-positive test result; not consuming ⩾7 alcoholic beverages per day and undergoing sufficiently frequentAST tests to detect hepatotoxicity	170	Hepatotoxicity
Yimer, 2011	Ethiopia	Prospective cohort	followed up for development of DILI for up to 56 weeks	All study participants received RMP based short-course chemotherapy for TB following the national TB treatment guideline. ART was then initiated	Newly diagnosed ART and anti-tuberculosis treatment-naïve adult TB and HIV co-infected patients. The eligibility criteria were age >18 years, CD4 count, 200 cells/Ul, not pregnant and not on other known hepatotoxic drugs concurrently (except cotrimoxazole, 960 mg per day, which was given to all participants before enrolment and during the follow-up period according to the treatment guideline)	353	Anti-tubercular and antiretroviral DILI
Yuliwulandari, 2016	Indonesia	Case-control	NR	NR (anti-tuberculosis treatment)	Patients included in the study were male and female aged 15–70 years, suffering from TB according to the WHO standards, and were under monitored treatment with anti-tuberculosis drugs Exclusion criteria: 1) history of liver disease such as: hepatitis A B or C; hepatoma; liver cirrhosis or cholelithiasis positive; 2) abnormal levels of any LFT (ALT, AST or total bilirubin) before anti-tuberculosis treatment	241	ATLI
Zaverucha-do-Valle, 2014	Brazil	Retrospective cohort	Follow-up at days 15 and 30 and at least one visit monthly until the end of treatment	600 mg/day of RMP, 400 mg/day of INH and 2 g/day of PZA for all patients with corporal weight >45 kg or adjusted for corporal weight <45 kg. After 2 months of treatment, PZA was discontinued	Inclusion criteria: signed written consent, sputum smear with AFB or culture positive for M. tuberculosis; ongoing TB treatment and laboratory tests of liver function Exclusion criteria: age <18 years, pregnancy and no >1 visit registered	131	ATDH

INH =isoniazid; RMP =rifampicin; PZA =pyrazinamide; EMB =ethambutol; ALT =alanine aminotransferase; AST =aspartate aminotransferase; RCT =randomised controlled trial; HIV =human immunodeficiency virus; SM =streptomycin; PTB =pulmonary tuberculosis; IU =international unit; ALP =alkaline phosphatase; DILI =drug-induced liver injury; LFT =liver function test; DIH =drug-induced hepatotoxicity; NR =not reported; ATDH =anti-tuberculosis drug-induced hepatotoxicity; GI =group identifier; ADR =adverse drug reaction; ULN =upper limit of normal; EPTB =extra-pulmonary TB; ADIH =anti-tuberculosis drug-induced hepatitis; ATD =anti-tuberculosis drug; MPE =maculopapular eruption; WHO = World Health Organization; gGTP = gamma-glutamyltranspeptidase; CMV = cytomegalovirus ; LTBI = latent tuberculous infection; ART =antiretroviral therapy; ATLI = anti-tuberculosis drug-induced liver injury.

**Table A.3:** (*continued*)

Author, year	Country	Study design	Follow-up time	Drugs and dosage	Selection criteria	Sample size *n*	Toxicity outcomes
An, 2012	China	App	6 months	Daily treatment with INH, RMP, PZA and EMB for 2 months, followed by 4 months treatment with INH and RMP, with drug dosages calculated according to body weight Body weight < 45 kg: RMP 300 mg, INH 200 mg, PZA 1000 mg Body weight of 45–55 kg: RMP 450 mg, INH 300 mg, PZA 1500 mg Body weight > 55 kg: RMP 600 mg, INH 400 mg, PZA 2000 mg	Inclusion criteria: Normal serum ALT, AST and bilirubin levels, no symptoms related to abnormal liver function (i.e., jaundice) before anti-tuberculosis drug treatment and close monitoring of changes in liver functions within 6 months of treatment Patients with and without hepatotoxicity during treatment Exclusion criteria: Malnutrition HIV type 1 infection Alcoholic liver disease or habitual drinking Hepatitis B or C infection, liver disease, systemic diseases and/or treatment with drugs other than the anti-tuberculosis drugs that can induce hepatotoxicity Severe TB or cardiac dysfunction that may cause liver dysfunction Transient increases in ALT	208	Hepatotoxocity
Azuma, 2013	Japan	RCT	6 months	All patients were treated with a 6-month regimen comprising INH, RMP, PZA and EMB/SM for the first 2 months, followed by RMP and INH for 4 months. All patients were started on the standard oral dose (~5 mg/kg body weight, once-daily). For pharmacogenetics-treatment patients, dosages were adjusted based on individual *NAT2* status within 3 days. Modified daily INH doses were respectively ~7.5, ~5 and ~2.5 mg/kg for rapid, intermediate and slow acetylators. Regarding the other drugs for the standard regimen, standard daily doses of RMP (10 mg/kg, maximum 600 mg/body), PZA (25 mg/kg, 1500 mg/body), EMB (15 mg/kg, 750 mg/body; 20 mg/kg, 1000 mg/body) and SM (15 mg/kg, 750 mg/body) were recommended with the following dose ranges allowed at the discretion of the physician in charge: RMP 8–12 mg/kg; PZA 20–30 mg/kg; EMB 15–20 mg/kg; SM 12–18 mg/kg	The eligible population was identified from a series of patients newly diagnosed with PTB (male and female aged 20–75 years), requiring the 6-month 4-drug standard treatment for the first time Exclusion criteria: abnormal test results for liver and kidney function (serum AST > 45 IU/l, ALT > 50 IU/l, ALP > 444 IU/l, total bilirubin > 1.6 mg/dl and creatinine > 1.4 mg/dl before the study treatments commenced, long-term use of steroids and/or immunosuppressants, inadequate clinical conditions such as hyperglycaemia, diabetes mellitus, acute life-threatening chronic progressive disease, pregnancy or lactation and alcoholism. Patients not expected to complete the study protocol for social reasons were not recruited	172	INH-DILI Peripheral neuropathy
Bose, 2011	India	Prospective cohort	Patients were followed up to 8 weeks after they had started with anti-tuberculosis treatment	All patients received anti-tuberculosis treatment (RMP, INH, EMB and PZA) according to body weight. All four drugs were given for 2 months. PZA and EMB were discontinued, while INH and RMP were continued for another 4 months RMP doses as follows: ⩽35 kg: 300 mg/day 36–50 kg: 450 mg/day >50 kg: 600 mg/day INH doses as follows: ⩽35 kg: 200 mg/day >35 kg: 400 mg/day PZA doses as follows: ⩽50 kg: 1.0 g/day >50 kg: 1.5 g/day EMB 20 mg/kg/day	Newly diagnosed patients of PTB. The baseline LFT of the patients was normal when they were started on anti-tuberculosis treatment Exclusion criteria: no history of habitual alcoholism, chronic liver diseases or steatosis	218	ADIH ADIH outcome
Çetintaş, 2008	Turkey	Prospective cohort	NR	Patients received INH 5 mg/kg (maximum 300 mg/day), RMP 10 mg/kg (maximum 600 mg/day), PZA 25 mg/kg (maximum 2000 mg/day), EMB 15–25 mg/kg (maximum 1500 mg/day)	Patients diagnosed with TB. Only patients with serum levels before initiation of treatment within the following ranges were included in the study: ALT 0–40 U/l, AST 5–45 U/l and total bilirubin 0.2–1.6 mg/dl Exclusion criteria: patients with positive serum hepatitis B surface antigen, hepatitis C antibody or hepatitis A immunoglobulin M antibody, and patients with alcoholic liver disease or any hepatic or systemic disease that could cause liver function disorder	100	DIH
Chamorro, 2013	Argentina	Prospective cohort	NR	The patients began a standard anti-tuberculosis treatment protocol for the first 2 months (INH 5 mg/kg/days, maximum 300 mg/day, RMP 10 mg/kg/day maximum 600 mg/day, PZA 20 mg/kg/day, EMB 20 mg/kg/day), followed by INH and RMP for ⩾4 months, depending on the disease severity or the presence ofextra-pulmonary foci	Inclusion criteria: TB patients aged >18 years with stable haemodynamic levels, normal renal function and negative for pregnancy Exclusion criteria: presence of diseases that directly affected the liver (acute hepatitis A, active hepatitis B and C; cirrhosis; encephalopathy and cancer), INH allergy, HIV, autoimmunity, concomitant hepatotoxic medications, a history of TB treatment failures, refusal of blood extraction and refusal to sign the written informed consent	175	ATDH
Chang, 2012	Taiwan	Prospective cohort	NR	First-line anti-tuberculosis medications	Patients diagnosed with TB and treated with first-line anti-tuberculosis medications Exclusion criteria: pregnancy, abnormal liver function and history of positive viral hepatitis before starting treatment with first-line anti-tuberculosis drugs	98	ATDH
Cho, 2007	Korea	Prospective cohort	Serum AST, ALT and total bilirubin levels were then monitored monthly until the end of treatment	All patients received oral INH (300 mg), RMP (600 mg), PZA (20 mg/kg body weight) and EMB (800 mg) daily for the first 2 months. PZA was then discontinued, while INH, RMP and EMB were continued for another 4 months	Adult patients newly diagnosed with active TB, with evident lesion of TB using simple X-ray or computed tomography or positive results on sputum smear or culture for detection of mycobacteria Exclusion criteria: 1) abnormal serum ALT, AST, or bilirubin levels or symptoms related to abnormal liver function such as jaundice before anti-tuberculosis treatment; 2) alcoholic liver disease or habitual alcohol drinking; 3) any other hepatic or systemic diseases that may cause liver dysfunction	132	ATDH
Costa, 2012	Brazil	Prospective cohort	NR	All patients were treated with the first-line anti-tuberculosis drug regimen INH (300 mg/kg/day), RMP (300 mg/kg/day) and PZA (1500 mg/kg/day) for the first 2 months, followed by INH and RMP for a further 4 months	Male or female subjects aged ⩾18 years, with no previously described renal, allergic or hepatic diseases and not pregnant	129	ADRs
Dhoro, 2013	Zimbabwe	Case-control	NR	NR	TB patients	Not clear—189 received INH	Peripheral neuropathy
Feng, 2014	China	Case-control	6 months	Treatment with anti-tuberculosis drug regimens at the usual dosage—300 mg/day INH, 450 mg/day RMP and 1500 mg/day PZA	Selection of cases: cases selected based on liver functions, i.e., all indices of liver function were normal before anti-tuberculosis chemotherapy, and became abnormal indicating hepatic injury after 6 months of chemotherapy. Cases were patients who showed ATDH based on increased serum transaminase values that were three-fold higher than the ULN (40 IU/l ALT) and symptoms compatible with hepatitis Selection of controls: controls underwent the same anti-tuberculosis chemotherapy with selected cases and were not tested for abnormality in liver functions after 6 months of the chemotherapy. The controls selected matched the criteria compared to the cases: 1) sex; 2) age discrepancy of <5 years; 3) living in the same regions; and 4) treatment with anti-tuberculosis drug regimens at the usual dosage, including 300 mg/day INH, 450 mg/day RMP and 1500 mg/day PZA	346	ATDH
Fredj, 2016	Tunisia	Prospective cohort	Serum AST, ALT and ALP were monitored monthly until the end of the treatment	The anti-tuberculosis treatment was based on the association of INH (5 mg/kg/day), RMP (10 mg/kg/day), PZA (25 mg/kg/day) and EMB (15 mg/kg/day) for the first 2 months, followed by INH and RMP for 4–7 additional months, depending on TB clinical presentation	Patients diagnosed with PTB and EPTB	71	INH-induced-hepatotoxicity
Gupta, 2013 (GI: GUPTA)	India	Prospective cohort	The patients were monitored for ALT, AST, andtotal bilirubin levels weekly for 1 month and then monthly until the completion of treatment	Initially patients received a combination regimen including INH 5 mg/kg (max 300 mg daily), RMP 10 mg/kg (max 600 mg daily), PZA 25 mg/kg (max 1500 mg daily) and EMB 15–25 mg/kg (max 2000 mg daily) for a period of 2 months, followed by an additional 4 months with INH and RMP	Inclusion criteria: 1) age > 18 years; 2) smear and/or culture positive for mycobacteria in clinical samples; and 3) normal ALT, AST and total bilirubin levels Exclusion criteria: 1) patients presenting clinically and laboratory-confirmed chronic liver disease such as jaundice; 2) acute and chronic hepatitis B and/or C or HIV; 3) alcoholic liver diseases; 4) a rise of two times the ULN of ALT, AST and total bilirubin levels; 5) medication with anti-tuberculosis drugs before start of treatment and/or other potentially hepatotoxic drugs; and 6) refusal to provide blood sample or signed informed consent form	215	ATDH
Higuchi, 2007 (GI: HIGUCHI)	Japan	Prospective cohort	NR	Treated with a INH (400 mg/d) and RMP (450 mg/d) containing regimen for 6 or 9 months	Patients with new onset of PTB treated with a INH (400 mg/d) and RMP (450 mg/d) containing regimen for 6 or 9 months Exclusion criteria: patients with liver cirrhosis, chronic and acute hepatitis, alcoholic liver disease and other chronic liver diseases	100	ATDH Skin rash Eosinophilia
Ho, 2013	Taiwan	Prospective cohort	180 days	All patients received oral INH 300 mg, RMP 600 mg (or 450 mg if body weight was <50 kg), PZA 25 mg/kg of body weight (max daily dose 2000 mg) and EMB 15 mg/kg of body weight daily (max daily dose 1600 mg) for the first 2 months. PZA was then discontinued, whereas INH, RMP and EMB were continued for another 4 months	The inclusion criteria included a sputum smear with AFB or culture positive for M. tuberculosis, undergoing anti-tuberculosis treatment, including INH or starting treatment during the recruitment period, hepatitis serology recorded in the medical chart with signed written consent Patients were excluded if they were infected with hepatitis B virus, hepatitis C virus or HIV, had any other hepatic disease, or did not have at least two visits documented in the medical records	348	ATDH
Huang, 2003 (GI: HUANG)	Taiwan	Prospective cohort	Serum ALT, AST and total bilirubin levels were monitored monthly until the end of treatment or checked whenever patients had symptoms of suspected hepatitis	Their standard daily anti-tuberculosis regimen for the first 2 months included INH (300 mg), RMP (600 mg or 450 mg if body weight <50 kg), PZA (20 mg/kg body weight) and EMB (25 mg/kg body weight). PZA was then discontinued, whereas INH, RMP, and EMB (15 mg/kg body weight) were continued for another 4 months	Patients with incident PTB or EPTB Exclusion criteria: 1) abnormal serum ALT, AST or bilirubin before anti-tuberculosis treatment; and 2) refusal of blood sampling or informed written consent	318	ADIH
Jung, 2015	Korea	Prospective cohort	4 weeks	Standard 4-drug treatment for 6 months: INH (5 mg/kg, usually 300 mg), RMP (450 mg for <50 kg or 600 mg for ⩾50 kg of body weight), EMB (15 mg/kg) and PZA (20–30 mg/kg), daily for 2 months, followed by INH and RMP with or without EMB for 4 months. Doses were adjusted when applying model-based treatment	Eligible participants were patients newly diagnosed with active TB, who underwent standard 4-drug treatment for 6 months Exclusion criteria: patients with abnormal hepatic function on laboratory testing (increased serum AST, ALT or total bilirubin) before anti-tuberculosis treatment and underlying liver disease or systemic illness such as congestive heart failure, acute life-threatening disease, or alcoholism or disease that was resistant to INH at the start of treatment	206	ATDH
Khalili, 2011	Iran	Case-control	2 months	Treated daily with INH (300 mg), RMP (600 mg), PZA (20 mg/kg), EMB (15 mg/kg) for the first 2 months, followed by INH and RMP daily for 4 additional months	Inclusion criteria: newly diagnosed patients (>18 years) with active PTB, who had been planned to be treated daily with anti-tuberculosis treatment (see drugs and dosage) Exclusion criteria: other possibilities of hepatitis, including patients with HIV infection, hepatic insufficiency (ALT or AST > 2× ULN) or clinically signs and symptoms of liver diseases such as jaundice and ascites, chronic hepatitis (B or C) and renal insufficiency (creatinine clearance < 50 ml/min based on Cockcroft-Gault equation) and history or current use of herbal, supplemental and hepatotoxic non-tuberculosis drugs	100	Hepatotoxicity
Kim, 2009 (GI: KIM)	Korea	Case-control	Assessments performed 2 weeks after onset of treatment and bi-monthly thereafter	All patients with PTB were treated daily with a combination regimen comprising INH (300–400 mg daily), RMP (450–600 mg daily), EMB (600–800 mg daily) and PZA (1000–1500 mg daily) for 2 months and then without PZA for ⩾4 following months. Doses of each drug were adjusted based on body weight of the patient	Newly diagnosed and treated patients with PTB. Exclusion criteria: patients with active or chronic hepatitis, including alcoholic hepatitis, fatty liver disease, liver cirrhosis, carriers of the hepatitis B or C virus, heavy alcohol intake, decreased renal function and severe cardiac diseases requiring several medications	226	ATDH
Kim, 2011 (GI: KIM)	Korea	Case-control	NR	The treatment comprised an initial phase of 2 months and a subsequent continuation phase of ⩾4 months. During the initial phase, 4 drugs were administered, including INH (300–400 mg daily), RMP (450–600 mg daily), EMB (600–800 mg daily) and PZA (1000–1500 mg daily). Doses of each drug were adjusted based on the body weight of the patient. In the following continuation phase, only PZA was discontinued, while the other three drugs were continued	Patients newly diagnosed with PTB and/or TB pleuritis and treated with first-line anti-tuberculosis medications such as INH, RMP, EMB and PZA Exclusion criteria: 1) patients with skin diseases before treatment, 2) chronic renal failure and chronic liver diseases affecting drug metabolism, 3) chronic alcoholism, 4) other chronic medical conditions requiring medication, and 5) non-adherence to treatment	221	ATD-induced MPE
Lee, 2010	Taiwan	Prospective cohort	NR	All patients received oral INH 300 mg, RMP 600 mg (or 450 mg, if body weight was <50 kg), PZA 200 mg/kg of body weight and EMB 800 mg daily for the first 2 months. PZA was then discontinued, while INH, RMP and EMB were continued for another 4 months	Inclusion criteria: adult patients newly diagnosed with active TB, having evident lesions of TB using simple X-ray, computed tomography, sputum smears and cultures positive for mycobacteria Exclusion criteria: 1) positive serum hepatitis B virus surface antigen, antibody to hepatitis C virus; 2) alcoholic liver disease or habitual alcohol drinking; 3) any other hepatic or systemic diseases that may cause liver dysfunction; 4) abnormal serum ALT, AST or bilirubin levels before anti-tuberculosis treatment	140	ATDH
Leiro-Fernandez, 2011	Spain	Case-control	Routine follow-up of clinical assessments every 2 weeks during the first month and monthly thereafter untilcompletion of treatment	Treatment with regimens that included INH, RMP and PZA at the usual dosages (INH 5 mg/kg/day to maximum 300 mg/day, RMP10 mg/kg/day to maximum 600 mg/day and PZA 25–30 mg/kg/day to maximum 2500 mg/day	Inclusion criteria: 1) age 15–75 years; 2) microbiological demonstration of active TB; 3) treatment with regimens that included INH, RMP and PZA at the usual dosages (INH 5 mg/kg/day to max 300 mg/day, RMP 10 mg/kg/day to max 600 mg/day and PZA 25–30 mg/kg/day to max 2500 mg/day); and 4) adequate treatment adherence Exclusion criteria: 1) increased baseline serum transaminases (AST and/or ALT; normal values ⩽ 40 IU/l); 2) positive serological testing for HIV, hepatitis B virus or hepatitis C virus; 3) regular alcohol intake or concomitant use of hepatotoxic drugs; 4) history of chronic liver disease; 5) pregnancy; or 6) no or poor adherence to treatment	117	ATDH
Lv, 2012	China	Case-control	6–9 months	All primary/retreatment patients took INH (600 mg), RMP (600 mg or 450 mg if body weight was <50 kg), PZA (2000 mg) and EMB (1250 mg) every other day in the first 2 months and then INH and RMP were continued for another 4/6 months. The retreatment patients received SM (750 mg) every other day in the first 2 months and continued receiving EMB for another 6 months	Inclusion criteria: sputum smear-positive PTB patients who received standard short-course chemotherapy recommended by the WHO Exclusion criteria: 1) positive serum hepatitis B virus surface antigen or other liver disease, 2) potentially hepatotoxic medications that would confound the picture, 3) abnormal serum ALT, AST or total bilirubin levels before anti-tuberculosis treatment	445	ATDH
Mahmoud, 2012	Tunisia	Prospective cohort	3 months	Treated with INH and RMP containing regimen	Inclusion criteria: adult patients (>18 years) newly diagnosed with TB treated with INH- and RMP-containing regimen, LFTs before initiation of treatment showed completely normal findings on serum ALT, AST, total bilirubin, ALP and gGTP Exclusion criteria: 1) patients receiving other potentially hepatotoxic drugs in addition to anti-tuberculosis agents; 2) positive serum hepatitis B surface antigen or hepatitis C antibody; 3) alcoholic liver disease; 4) any other hepatic or systemic diseases that may cause liver dysfunction	66	INH hepatotoxicity
Ng, 2014	Cases recruited in the UK; controls were samples from elsewhere	Case-control	NR	All had been prescribed INH with all but one also taking additional anti-tuberculosis drugs. Patients were prescribed 300 mg INH/day in line with UK/WHO guidelines	Cases: patients diagnosed with DILI either in the past or at the time of sample collection. Onlycases assessed as having DILI that was highly probably, probably or possibly relating to anti-tuberculosis drug exposure were enrolled in the study. Other possible causes of liver toxicity, particularly hepatitis A, B, C and CMV infection were excluded. No patients included were HIV-positive or had pre-existing liver disease. None had been treated for TB under directly observed treatment Controls: 52 DNA samples from individuals of European ancestry; inclusion/exclusion criteria not reported	127	DILI
Ohno, 2000	Japan	Prospective cohort	3 months	Initial chemotherapy always included INH (400 mg/day; 8.2 ± 2.0 mg/kg/day) and RMP (450 mg/day; 9.2 ± 2.2 mg/kg/day); the third drug used was EMB or SM.	Inclusion criteria: patients had no history of alcohol abuse and were negative for hepatitis B surface antigen and hepatitis C antibody. At the beginning of treatment, LFTs showed completely normal findings on serum AST, ALT, bilirubin, ALP and gGTP Patients receiving other potentially hepatopathic drugs in addition to anti-tuberculosis agents were excluded from the study	77	INH + RMP-induced hepatotoxicity
Possuelo, 2008 (GI: POSSUELO)	Brazil	Prospective cohort	NR	Treatment daily with INH, RMP and PZA for the first 2 months followed by INH and RMP daily for 4 additional months. Drug dosages used were calculated according to patient's weight (weight <45 kg: RMP 300 mg, INH 200 mg, PZA 1000 mg; 45–55 kg: RMP 450 mg, INH 300 mg, PZA 1500 mg; >55 kg: RMP 600 mg, INH 400 mg, PZA 2000 mg)	Inclusion criteria: adult patients (>18 years) newly diagnosed with active TB, who had been treated daily with anti-tuberculosis treatment (see drugs and dosage) Exclusion criteria: patients presenting clinically and laboratory-confirmed liver chronic disease, patients using anti-tuberculosis drugs before enrolment in the study, patients presenting results of LFTs before the beginning of treatment higher that were 2× ULN and refusal to participate of the study	254	ATDH Gastrointestinal ADRs
Rana, 2014 (GI: RANA)	India	Prospective cohort	Patients were monitored every month until the end of treatment or whenever the patients had symptoms or signs of hepatotoxicity	Daily anti-tuberculosis treatment for the first 2 months included INH (300 mg), RMP (600 or 450 mg for body weight/50 kg), PZA (20 mg/kg body weight) and EMB (25 mg/kg body weight). After 2 months, EMB and PZA were discontinued, whereas INH and RMP were continued for an additional 4 months	Inclusion criteria: patients with pulmonary and EPTB Exclusion criteria: patients with history of alcohol abuse and/or any other liver disease; patients who had received other potentially hepatotoxic drugs in addition to anti-tuberculosis drugs; patients who had abnormal serum ALT, AST or bilirubin before starting anti-tuberculosis treatment; patientswho developed viral hepatitis during anti-tuberculosis treatment; patients with renal failure or cancer; and patients who refused blood sampling or providing informed written consent	300	Hepatotoxicity
Santos, 2013 (GI: SANTOS)	Brazil	Prospective cohort	NR	Treatment with INH, RMP and PZA for the first 2 months, followed by INH and RMP daily for 4 months	Inclusion criteria: patients diagnosed with TB and treated with anti-tuberculosis treatment (see drugs and dosage) Exclusion criteria: patients aged <18 years, those with mental disabilities, chronic liver disease confirmed by clinical and laboratory data, users of anti-tuberculosis drugs before enrolment in the study, and those with liver function results > 2× ULN before beginning treatment	270	ATDH
Shimizu, 2006	Japan	Prospective cohort	3 months	Treatment with INH (300–400 mg) and RMP (300–450 mg)	Individuals with PTB without abnormal serum levels of ALT, AST, direct or total bilirubin, or serum hepatitis B virus surface antigen or antibody to hepatitis C virus at the beginning of the study	42	Hepatotoxicity
Singla, 2014	India	Prospective cohort	NR	NR	Inclusion criteria: newly diagnosed patients with TB Exclusion criteria: history of heavy use of alcohol or chronic liver diseases or liver cirrhosis;patients who were settled in the area for a minimum of three generations; people infected with HIV	408	ATDH
Sotsuka, 2011	Japan	Prospective cohort	3 months	INH, RMP and PZA, plus EMB or SM during the first 2 months, followed by administration of INH and RMP plus EMB or SM during the final 4 months	Inclusion criteria: in-patients with active PTB who were treated with the standard Japanese chemotherapy regimen, followed up for >3 months after treatment, and who consented to this study	144	Hepatotoxicity
Teixeira, 2011	Brazil	Case-control	NR	Anti-tuberculosis drug regimens that include INH at the usual dosage (400 mg/day)	Inclusion criteria: 1) age > 18 years, 2) diagnosis of active TB, 3) treatment with anti-tuberculosis drug regimens that include INH at the usual dosage (400 mg/day) and 4) normal baseline serum transaminases (ALT and AST) before treatment Exclusion criteria: 1) positive serological test for the HIV, hepatitis B virus or hepatitis C virus, 2) alcohol abuse, 3) history of chronic liver disease and 4) pregnancy	167	Hepatitis
Vuilleumier, 2006	Switzerland	Prospective cohort	NR	INH 300 mg daily and vitamin B6 40 mg per day for a period of 6 months	Inclusion criteria: patients having LTBI as defined by the American Thoracic Society with normal plasma AST and ALT levels before the beginning of INH monotherapy Exclusion criteria: 1) a history of alcohol consumption, 2) positive serology for the HAV, HBV or HCV, 3) poor chemotherapy compliance (negative urine INH 3 times during the follow-up), loss during the follow-up, 4) patients had to receive supplementary anti-tuberculosis agents or other potentially hepatotoxic drugs	89	INH-induced hepatotoxicity
Wang, 2015 (GI: NTUH)	Taiwan	Prospective cohort	6 months	Daily INH, RMP, EMB and PZA in the first 2 months, and daily INH and RMP for the next 4 months The daily dosage of each drug was calculated by weight	Adult Taiwanese (>16 years) patients with culture-confirmed PTB Subjects were excluded if they were pregnant, had a life expectancy < 6 months, had abnormal baseline LFT or were resistant to INH, RMP or both	355 in the derivation cohort, 182 in the validation cohort	Hepatotoxicity during anti-tuberculosis treatment
Xiang, 2014	China	Prospective cohort	2 months	All patients were prescribed INH (600 mg), RMP (600 mg or 450 mg if the body weight was <50 kg), PZA (2000 mg) and EMB (1250 mg) every other day in the first 2 months. After 2 months, INH and RMP were continued for a further 4–6 months. Retreatment patients in addition received SM (750 mg) every other day in the first 2 months and continued receiving EMB for another 6 months	Inclusion criteria: newly diagnosed PTB patients belonging to the Uyghur ethnic group, who were receiving standard short-course chemotherapy recommended by the WHO, who attended for a 2-month assessment, and any patients attending clinic with suspected liver disease after the start of treatment, before the 2-month visit Exclusion criteria: patients who had signs of abnormal liver function when they started treatment (jaundice or elevated ALT, AST or bilirubin levels) or disease associated with liver dysfunction	2244	ATLI
Yamada, 2009 (GI: YAMADA)	Canada	Prospective cohort	9 months	INH 300 mg	Subjects who were receiving treatment with INH 300 mg daily self-administered for LTBI.Subjects were included if they were aged ⩾19 years; not concurrently receiving other anti-tuberculosis drugs; non-reactive to hepatitis B surface antigen and antibody to hepatitis C virus; absence of any liver or metabolic diseases; without a HIV-positive test result; not consuming ⩾7 alcoholic beverages per day and undergoing sufficiently frequentAST tests to detect hepatotoxicity	170	Hepatotoxicity
Yimer, 2011	Ethiopia	Prospective cohort	followed up for development of DILI for up to 56 weeks	All study participants received RMP based short-course chemotherapy for TB following the national TB treatment guideline. ART was then initiated	Newly diagnosed ART and anti-tuberculosis treatment-naïve adult TB and HIV co-infected patients. The eligibility criteria were age >18 years, CD4 count, 200 cells/Ul, not pregnant and not on other known hepatotoxic drugs concurrently (except cotrimoxazole, 960 mg per day, which was given to all participants before enrolment and during the follow-up period according to the treatment guideline)	353	Anti-tubercular and antiretroviral DILI
Yuliwulandari, 2016	Indonesia	Case-control	NR	NR (anti-tuberculosis treatment)	Patients included in the study were male and female aged 15–70 years, suffering from TB according to the WHO standards, and were under monitored treatment with anti-tuberculosis drugs Exclusion criteria: 1) history of liver disease such as: hepatitis A B or C; hepatoma; liver cirrhosis or cholelithiasis positive; 2) abnormal levels of any LFT (ALT, AST or total bilirubin) before anti-tuberculosis treatment	241	ATLI
Zaverucha-do-Valle, 2014	Brazil	Retrospective cohort	Follow-up at days 15 and 30 and at least one visit monthly until the end of treatment	600 mg/day of RMP, 400 mg/day of INH and 2 g/day of PZA for all patients with corporal weight >45 kg or adjusted for corporal weight <45 kg. After 2 months of treatment, PZA was discontinued	Inclusion criteria: signed written consent, sputum smear with AFB or culture positive for M. tuberculosis; ongoing TB treatment and laboratory tests of liver function Exclusion criteria: age <18 years, pregnancy and no >1 visit registered	131	ATDH

INH =isoniazid; RMP =rifampicin; PZA =pyrazinamide; EMB =ethambutol; ALT =alanine aminotransferase; AST =aspartate aminotransferase; RCT =randomised controlled trial; HIV =human immunodeficiency virus; SM =streptomycin; PTB =pulmonary tuberculosis; IU =international unit; ALP =alkaline phosphatase; DILI =drug-induced liver injury; LFT =liver function test; DIH =drug-induced hepatotoxicity; NR =not reported; ATDH =anti-tuberculosis drug-induced hepatotoxicity; GI =group identifier; ADR =adverse drug reaction; ULN =upper limit of normal; EPTB =extra-pulmonary TB; ADIH =anti-tuberculosis drug-induced hepatitis; ATD =anti-tuberculosis drug; MPE =maculopapular eruption; WHO = World Health Organization; gGTP = gamma-glutamyltranspeptidase; CMV = cytomegalovirus ; LTBI = latent tuberculous infection; ART =antiretroviral therapy; ATLI = anti-tuberculosis drug-induced liver injury.

**Table A.3:** (*continued*)

Author, year	Country	Study design	Follow-up time	Drugs and dosage	Selection criteria	Sample size *n*	Toxicity outcomes
An, 2012	China	App	6 months	Daily treatment with INH, RMP, PZA and EMB for 2 months, followed by 4 months treatment with INH and RMP, with drug dosages calculated according to body weight Body weight < 45 kg: RMP 300 mg, INH 200 mg, PZA 1000 mg Body weight of 45–55 kg: RMP 450 mg, INH 300 mg, PZA 1500 mg Body weight > 55 kg: RMP 600 mg, INH 400 mg, PZA 2000 mg	Inclusion criteria: Normal serum ALT, AST and bilirubin levels, no symptoms related to abnormal liver function (i.e., jaundice) before anti-tuberculosis drug treatment and close monitoring of changes in liver functions within 6 months of treatment Patients with and without hepatotoxicity during treatment Exclusion criteria: Malnutrition HIV type 1 infection Alcoholic liver disease or habitual drinking Hepatitis B or C infection, liver disease, systemic diseases and/or treatment with drugs other than the anti-tuberculosis drugs that can induce hepatotoxicity Severe TB or cardiac dysfunction that may cause liver dysfunction Transient increases in ALT	208	Hepatotoxocity
Azuma, 2013	Japan	RCT	6 months	All patients were treated with a 6-month regimen comprising INH, RMP, PZA and EMB/SM for the first 2 months, followed by RMP and INH for 4 months. All patients were started on the standard oral dose (~5 mg/kg body weight, once-daily). For pharmacogenetics-treatment patients, dosages were adjusted based on individual *NAT2* status within 3 days. Modified daily INH doses were respectively ~7.5, ~5 and ~2.5 mg/kg for rapid, intermediate and slow acetylators. Regarding the other drugs for the standard regimen, standard daily doses of RMP (10 mg/kg, maximum 600 mg/body), PZA (25 mg/kg, 1500 mg/body), EMB (15 mg/kg, 750 mg/body; 20 mg/kg, 1000 mg/body) and SM (15 mg/kg, 750 mg/body) were recommended with the following dose ranges allowed at the discretion of the physician in charge: RMP 8–12 mg/kg; PZA 20–30 mg/kg; EMB 15–20 mg/kg; SM 12–18 mg/kg	The eligible population was identified from a series of patients newly diagnosed with PTB (male and female aged 20–75 years), requiring the 6-month 4-drug standard treatment for the first time Exclusion criteria: abnormal test results for liver and kidney function (serum AST > 45 IU/l, ALT > 50 IU/l, ALP > 444 IU/l, total bilirubin > 1.6 mg/dl and creatinine > 1.4 mg/dl before the study treatments commenced, long-term use of steroids and/or immunosuppressants, inadequate clinical conditions such as hyperglycaemia, diabetes mellitus, acute life-threatening chronic progressive disease, pregnancy or lactation and alcoholism. Patients not expected to complete the study protocol for social reasons were not recruited	172	INH-DILI Peripheral neuropathy
Bose, 2011	India	Prospective cohort	Patients were followed up to 8 weeks after they had started with anti-tuberculosis treatment	All patients received anti-tuberculosis treatment (RMP, INH, EMB and PZA) according to body weight. All four drugs were given for 2 months. PZA and EMB were discontinued, while INH and RMP were continued for another 4 months RMP doses as follows: ⩽35 kg: 300 mg/day 36–50 kg: 450 mg/day >50 kg: 600 mg/day INH doses as follows: ⩽35 kg: 200 mg/day >35 kg: 400 mg/day PZA doses as follows: ⩽50 kg: 1.0 g/day >50 kg: 1.5 g/day EMB 20 mg/kg/day	Newly diagnosed patients of PTB. The baseline LFT of the patients was normal when they were started on anti-tuberculosis treatment Exclusion criteria: no history of habitual alcoholism, chronic liver diseases or steatosis	218	ADIH ADIH outcome
Çetintaş, 2008	Turkey	Prospective cohort	NR	Patients received INH 5 mg/kg (maximum 300 mg/day), RMP 10 mg/kg (maximum 600 mg/day), PZA 25 mg/kg (maximum 2000 mg/day), EMB 15–25 mg/kg (maximum 1500 mg/day)	Patients diagnosed with TB. Only patients with serum levels before initiation of treatment within the following ranges were included in the study: ALT 0–40 U/l, AST 5–45 U/l and total bilirubin 0.2–1.6 mg/dl Exclusion criteria: patients with positive serum hepatitis B surface antigen, hepatitis C antibody or hepatitis A immunoglobulin M antibody, and patients with alcoholic liver disease or any hepatic or systemic disease that could cause liver function disorder	100	DIH
Chamorro, 2013	Argentina	Prospective cohort	NR	The patients began a standard anti-tuberculosis treatment protocol for the first 2 months (INH 5 mg/kg/days, maximum 300 mg/day, RMP 10 mg/kg/day maximum 600 mg/day, PZA 20 mg/kg/day, EMB 20 mg/kg/day), followed by INH and RMP for ⩾4 months, depending on the disease severity or the presence ofextra-pulmonary foci	Inclusion criteria: TB patients aged >18 years with stable haemodynamic levels, normal renal function and negative for pregnancy Exclusion criteria: presence of diseases that directly affected the liver (acute hepatitis A, active hepatitis B and C; cirrhosis; encephalopathy and cancer), INH allergy, HIV, autoimmunity, concomitant hepatotoxic medications, a history of TB treatment failures, refusal of blood extraction and refusal to sign the written informed consent	175	ATDH
Chang, 2012	Taiwan	Prospective cohort	NR	First-line anti-tuberculosis medications	Patients diagnosed with TB and treated with first-line anti-tuberculosis medications Exclusion criteria: pregnancy, abnormal liver function and history of positive viral hepatitis before starting treatment with first-line anti-tuberculosis drugs	98	ATDH
Cho, 2007	Korea	Prospective cohort	Serum AST, ALT and total bilirubin levels were then monitored monthly until the end of treatment	All patients received oral INH (300 mg), RMP (600 mg), PZA (20 mg/kg body weight) and EMB (800 mg) daily for the first 2 months. PZA was then discontinued, while INH, RMP and EMB were continued for another 4 months	Adult patients newly diagnosed with active TB, with evident lesion of TB using simple X-ray or computed tomography or positive results on sputum smear or culture for detection of mycobacteria Exclusion criteria: 1) abnormal serum ALT, AST, or bilirubin levels or symptoms related to abnormal liver function such as jaundice before anti-tuberculosis treatment; 2) alcoholic liver disease or habitual alcohol drinking; 3) any other hepatic or systemic diseases that may cause liver dysfunction	132	ATDH
Costa, 2012	Brazil	Prospective cohort	NR	All patients were treated with the first-line anti-tuberculosis drug regimen INH (300 mg/kg/day), RMP (300 mg/kg/day) and PZA (1500 mg/kg/day) for the first 2 months, followed by INH and RMP for a further 4 months	Male or female subjects aged ⩾18 years, with no previously described renal, allergic or hepatic diseases and not pregnant	129	ADRs
Dhoro, 2013	Zimbabwe	Case-control	NR	NR	TB patients	Not clear—189 received INH	Peripheral neuropathy
Feng, 2014	China	Case-control	6 months	Treatment with anti-tuberculosis drug regimens at the usual dosage—300 mg/day INH, 450 mg/day RMP and 1500 mg/day PZA	Selection of cases: cases selected based on liver functions, i.e., all indices of liver function were normal before anti-tuberculosis chemotherapy, and became abnormal indicating hepatic injury after 6 months of chemotherapy. Cases were patients who showed ATDH based on increased serum transaminase values that were three-fold higher than the ULN (40 IU/l ALT) and symptoms compatible with hepatitis Selection of controls: controls underwent the same anti-tuberculosis chemotherapy with selected cases and were not tested for abnormality in liver functions after 6 months of the chemotherapy. The controls selected matched the criteria compared to the cases: 1) sex; 2) age discrepancy of <5 years; 3) living in the same regions; and 4) treatment with anti-tuberculosis drug regimens at the usual dosage, including 300 mg/day INH, 450 mg/day RMP and 1500 mg/day PZA	346	ATDH
Fredj, 2016	Tunisia	Prospective cohort	Serum AST, ALT and ALP were monitored monthly until the end of the treatment	The anti-tuberculosis treatment was based on the association of INH (5 mg/kg/day), RMP (10 mg/kg/day), PZA (25 mg/kg/day) and EMB (15 mg/kg/day) for the first 2 months, followed by INH and RMP for 4–7 additional months, depending on TB clinical presentation	Patients diagnosed with PTB and EPTB	71	INH-induced-hepatotoxicity
Gupta, 2013 (GI: GUPTA)	India	Prospective cohort	The patients were monitored for ALT, AST, andtotal bilirubin levels weekly for 1 month and then monthly until the completion of treatment	Initially patients received a combination regimen including INH 5 mg/kg (max 300 mg daily), RMP 10 mg/kg (max 600 mg daily), PZA 25 mg/kg (max 1500 mg daily) and EMB 15–25 mg/kg (max 2000 mg daily) for a period of 2 months, followed by an additional 4 months with INH and RMP	Inclusion criteria: 1) age > 18 years; 2) smear and/or culture positive for mycobacteria in clinical samples; and 3) normal ALT, AST and total bilirubin levels Exclusion criteria: 1) patients presenting clinically and laboratory-confirmed chronic liver disease such as jaundice; 2) acute and chronic hepatitis B and/or C or HIV; 3) alcoholic liver diseases; 4) a rise of two times the ULN of ALT, AST and total bilirubin levels; 5) medication with anti-tuberculosis drugs before start of treatment and/or other potentially hepatotoxic drugs; and 6) refusal to provide blood sample or signed informed consent form	215	ATDH
Higuchi, 2007 (GI: HIGUCHI)	Japan	Prospective cohort	NR	Treated with a INH (400 mg/d) and RMP (450 mg/d) containing regimen for 6 or 9 months	Patients with new onset of PTB treated with a INH (400 mg/d) and RMP (450 mg/d) containing regimen for 6 or 9 months Exclusion criteria: patients with liver cirrhosis, chronic and acute hepatitis, alcoholic liver disease and other chronic liver diseases	100	ATDH Skin rash Eosinophilia
Ho, 2013	Taiwan	Prospective cohort	180 days	All patients received oral INH 300 mg, RMP 600 mg (or 450 mg if body weight was <50 kg), PZA 25 mg/kg of body weight (max daily dose 2000 mg) and EMB 15 mg/kg of body weight daily (max daily dose 1600 mg) for the first 2 months. PZA was then discontinued, whereas INH, RMP and EMB were continued for another 4 months	The inclusion criteria included a sputum smear with AFB or culture positive for M. tuberculosis, undergoing anti-tuberculosis treatment, including INH or starting treatment during the recruitment period, hepatitis serology recorded in the medical chart with signed written consent Patients were excluded if they were infected with hepatitis B virus, hepatitis C virus or HIV, had any other hepatic disease, or did not have at least two visits documented in the medical records	348	ATDH
Huang, 2003 (GI: HUANG)	Taiwan	Prospective cohort	Serum ALT, AST and total bilirubin levels were monitored monthly until the end of treatment or checked whenever patients had symptoms of suspected hepatitis	Their standard daily anti-tuberculosis regimen for the first 2 months included INH (300 mg), RMP (600 mg or 450 mg if body weight <50 kg), PZA (20 mg/kg body weight) and EMB (25 mg/kg body weight). PZA was then discontinued, whereas INH, RMP, and EMB (15 mg/kg body weight) were continued for another 4 months	Patients with incident PTB or EPTB Exclusion criteria: 1) abnormal serum ALT, AST or bilirubin before anti-tuberculosis treatment; and 2) refusal of blood sampling or informed written consent	318	ADIH
Jung, 2015	Korea	Prospective cohort	4 weeks	Standard 4-drug treatment for 6 months: INH (5 mg/kg, usually 300 mg), RMP (450 mg for <50 kg or 600 mg for ⩾50 kg of body weight), EMB (15 mg/kg) and PZA (20–30 mg/kg), daily for 2 months, followed by INH and RMP with or without EMB for 4 months. Doses were adjusted when applying model-based treatment	Eligible participants were patients newly diagnosed with active TB, who underwent standard 4-drug treatment for 6 months Exclusion criteria: patients with abnormal hepatic function on laboratory testing (increased serum AST, ALT or total bilirubin) before anti-tuberculosis treatment and underlying liver disease or systemic illness such as congestive heart failure, acute life-threatening disease, or alcoholism or disease that was resistant to INH at the start of treatment	206	ATDH
Khalili, 2011	Iran	Case-control	2 months	Treated daily with INH (300 mg), RMP (600 mg), PZA (20 mg/kg), EMB (15 mg/kg) for the first 2 months, followed by INH and RMP daily for 4 additional months	Inclusion criteria: newly diagnosed patients (>18 years) with active PTB, who had been planned to be treated daily with anti-tuberculosis treatment (see drugs and dosage) Exclusion criteria: other possibilities of hepatitis, including patients with HIV infection, hepatic insufficiency (ALT or AST > 2× ULN) or clinically signs and symptoms of liver diseases such as jaundice and ascites, chronic hepatitis (B or C) and renal insufficiency (creatinine clearance < 50 ml/min based on Cockcroft-Gault equation) and history or current use of herbal, supplemental and hepatotoxic non-tuberculosis drugs	100	Hepatotoxicity
Kim, 2009 (GI: KIM)	Korea	Case-control	Assessments performed 2 weeks after onset of treatment and bi-monthly thereafter	All patients with PTB were treated daily with a combination regimen comprising INH (300–400 mg daily), RMP (450–600 mg daily), EMB (600–800 mg daily) and PZA (1000–1500 mg daily) for 2 months and then without PZA for ⩾4 following months. Doses of each drug were adjusted based on body weight of the patient	Newly diagnosed and treated patients with PTB. Exclusion criteria: patients with active or chronic hepatitis, including alcoholic hepatitis, fatty liver disease, liver cirrhosis, carriers of the hepatitis B or C virus, heavy alcohol intake, decreased renal function and severe cardiac diseases requiring several medications	226	ATDH
Kim, 2011 (GI: KIM)	Korea	Case-control	NR	The treatment comprised an initial phase of 2 months and a subsequent continuation phase of ⩾4 months. During the initial phase, 4 drugs were administered, including INH (300–400 mg daily), RMP (450–600 mg daily), EMB (600–800 mg daily) and PZA (1000–1500 mg daily). Doses of each drug were adjusted based on the body weight of the patient. In the following continuation phase, only PZA was discontinued, while the other three drugs were continued	Patients newly diagnosed with PTB and/or TB pleuritis and treated with first-line anti-tuberculosis medications such as INH, RMP, EMB and PZA Exclusion criteria: 1) patients with skin diseases before treatment, 2) chronic renal failure and chronic liver diseases affecting drug metabolism, 3) chronic alcoholism, 4) other chronic medical conditions requiring medication, and 5) non-adherence to treatment	221	ATD-induced MPE
Lee, 2010	Taiwan	Prospective cohort	NR	All patients received oral INH 300 mg, RMP 600 mg (or 450 mg, if body weight was <50 kg), PZA 200 mg/kg of body weight and EMB 800 mg daily for the first 2 months. PZA was then discontinued, while INH, RMP and EMB were continued for another 4 months	Inclusion criteria: adult patients newly diagnosed with active TB, having evident lesions of TB using simple X-ray, computed tomography, sputum smears and cultures positive for mycobacteria Exclusion criteria: 1) positive serum hepatitis B virus surface antigen, antibody to hepatitis C virus; 2) alcoholic liver disease or habitual alcohol drinking; 3) any other hepatic or systemic diseases that may cause liver dysfunction; 4) abnormal serum ALT, AST or bilirubin levels before anti-tuberculosis treatment	140	ATDH
Leiro-Fernandez, 2011	Spain	Case-control	Routine follow-up of clinical assessments every 2 weeks during the first month and monthly thereafter untilcompletion of treatment	Treatment with regimens that included INH, RMP and PZA at the usual dosages (INH 5 mg/kg/day to maximum 300 mg/day, RMP10 mg/kg/day to maximum 600 mg/day and PZA 25–30 mg/kg/day to maximum 2500 mg/day	Inclusion criteria: 1) age 15–75 years; 2) microbiological demonstration of active TB; 3) treatment with regimens that included INH, RMP and PZA at the usual dosages (INH 5 mg/kg/day to max 300 mg/day, RMP 10 mg/kg/day to max 600 mg/day and PZA 25–30 mg/kg/day to max 2500 mg/day); and 4) adequate treatment adherence Exclusion criteria: 1) increased baseline serum transaminases (AST and/or ALT; normal values ⩽ 40 IU/l); 2) positive serological testing for HIV, hepatitis B virus or hepatitis C virus; 3) regular alcohol intake or concomitant use of hepatotoxic drugs; 4) history of chronic liver disease; 5) pregnancy; or 6) no or poor adherence to treatment	117	ATDH
Lv, 2012	China	Case-control	6–9 months	All primary/retreatment patients took INH (600 mg), RMP (600 mg or 450 mg if body weight was <50 kg), PZA (2000 mg) and EMB (1250 mg) every other day in the first 2 months and then INH and RMP were continued for another 4/6 months. The retreatment patients received SM (750 mg) every other day in the first 2 months and continued receiving EMB for another 6 months	Inclusion criteria: sputum smear-positive PTB patients who received standard short-course chemotherapy recommended by the WHO Exclusion criteria: 1) positive serum hepatitis B virus surface antigen or other liver disease, 2) potentially hepatotoxic medications that would confound the picture, 3) abnormal serum ALT, AST or total bilirubin levels before anti-tuberculosis treatment	445	ATDH
Mahmoud, 2012	Tunisia	Prospective cohort	3 months	Treated with INH and RMP containing regimen	Inclusion criteria: adult patients (>18 years) newly diagnosed with TB treated with INH- and RMP-containing regimen, LFTs before initiation of treatment showed completely normal findings on serum ALT, AST, total bilirubin, ALP and gGTP Exclusion criteria: 1) patients receiving other potentially hepatotoxic drugs in addition to anti-tuberculosis agents; 2) positive serum hepatitis B surface antigen or hepatitis C antibody; 3) alcoholic liver disease; 4) any other hepatic or systemic diseases that may cause liver dysfunction	66	INH hepatotoxicity
Ng, 2014	Cases recruited in the UK; controls were samples from elsewhere	Case-control	NR	All had been prescribed INH with all but one also taking additional anti-tuberculosis drugs. Patients were prescribed 300 mg INH/day in line with UK/WHO guidelines	Cases: patients diagnosed with DILI either in the past or at the time of sample collection. Onlycases assessed as having DILI that was highly probably, probably or possibly relating to anti-tuberculosis drug exposure were enrolled in the study. Other possible causes of liver toxicity, particularly hepatitis A, B, C and CMV infection were excluded. No patients included were HIV-positive or had pre-existing liver disease. None had been treated for TB under directly observed treatment Controls: 52 DNA samples from individuals of European ancestry; inclusion/exclusion criteria not reported	127	DILI
Ohno, 2000	Japan	Prospective cohort	3 months	Initial chemotherapy always included INH (400 mg/day; 8.2 ± 2.0 mg/kg/day) and RMP (450 mg/day; 9.2 ± 2.2 mg/kg/day); the third drug used was EMB or SM.	Inclusion criteria: patients had no history of alcohol abuse and were negative for hepatitis B surface antigen and hepatitis C antibody. At the beginning of treatment, LFTs showed completely normal findings on serum AST, ALT, bilirubin, ALP and gGTP Patients receiving other potentially hepatopathic drugs in addition to anti-tuberculosis agents were excluded from the study	77	INH + RMP-induced hepatotoxicity
Possuelo, 2008 (GI: POSSUELO)	Brazil	Prospective cohort	NR	Treatment daily with INH, RMP and PZA for the first 2 months followed by INH and RMP daily for 4 additional months. Drug dosages used were calculated according to patient's weight (weight <45 kg: RMP 300 mg, INH 200 mg, PZA 1000 mg; 45–55 kg: RMP 450 mg, INH 300 mg, PZA 1500 mg; >55 kg: RMP 600 mg, INH 400 mg, PZA 2000 mg)	Inclusion criteria: adult patients (>18 years) newly diagnosed with active TB, who had been treated daily with anti-tuberculosis treatment (see drugs and dosage) Exclusion criteria: patients presenting clinically and laboratory-confirmed liver chronic disease, patients using anti-tuberculosis drugs before enrolment in the study, patients presenting results of LFTs before the beginning of treatment higher that were 2× ULN and refusal to participate of the study	254	ATDH Gastrointestinal ADRs
Rana, 2014 (GI: RANA)	India	Prospective cohort	Patients were monitored every month until the end of treatment or whenever the patients had symptoms or signs of hepatotoxicity	Daily anti-tuberculosis treatment for the first 2 months included INH (300 mg), RMP (600 or 450 mg for body weight/50 kg), PZA (20 mg/kg body weight) and EMB (25 mg/kg body weight). After 2 months, EMB and PZA were discontinued, whereas INH and RMP were continued for an additional 4 months	Inclusion criteria: patients with pulmonary and EPTB Exclusion criteria: patients with history of alcohol abuse and/or any other liver disease; patients who had received other potentially hepatotoxic drugs in addition to anti-tuberculosis drugs; patients who had abnormal serum ALT, AST or bilirubin before starting anti-tuberculosis treatment; patientswho developed viral hepatitis during anti-tuberculosis treatment; patients with renal failure or cancer; and patients who refused blood sampling or providing informed written consent	300	Hepatotoxicity
Santos, 2013 (GI: SANTOS)	Brazil	Prospective cohort	NR	Treatment with INH, RMP and PZA for the first 2 months, followed by INH and RMP daily for 4 months	Inclusion criteria: patients diagnosed with TB and treated with anti-tuberculosis treatment (see drugs and dosage) Exclusion criteria: patients aged <18 years, those with mental disabilities, chronic liver disease confirmed by clinical and laboratory data, users of anti-tuberculosis drugs before enrolment in the study, and those with liver function results > 2× ULN before beginning treatment	270	ATDH
Shimizu, 2006	Japan	Prospective cohort	3 months	Treatment with INH (300–400 mg) and RMP (300–450 mg)	Individuals with PTB without abnormal serum levels of ALT, AST, direct or total bilirubin, or serum hepatitis B virus surface antigen or antibody to hepatitis C virus at the beginning of the study	42	Hepatotoxicity
Singla, 2014	India	Prospective cohort	NR	NR	Inclusion criteria: newly diagnosed patients with TB Exclusion criteria: history of heavy use of alcohol or chronic liver diseases or liver cirrhosis;patients who were settled in the area for a minimum of three generations; people infected with HIV	408	ATDH
Sotsuka, 2011	Japan	Prospective cohort	3 months	INH, RMP and PZA, plus EMB or SM during the first 2 months, followed by administration of INH and RMP plus EMB or SM during the final 4 months	Inclusion criteria: in-patients with active PTB who were treated with the standard Japanese chemotherapy regimen, followed up for >3 months after treatment, and who consented to this study	144	Hepatotoxicity
Teixeira, 2011	Brazil	Case-control	NR	Anti-tuberculosis drug regimens that include INH at the usual dosage (400 mg/day)	Inclusion criteria: 1) age > 18 years, 2) diagnosis of active TB, 3) treatment with anti-tuberculosis drug regimens that include INH at the usual dosage (400 mg/day) and 4) normal baseline serum transaminases (ALT and AST) before treatment Exclusion criteria: 1) positive serological test for the HIV, hepatitis B virus or hepatitis C virus, 2) alcohol abuse, 3) history of chronic liver disease and 4) pregnancy	167	Hepatitis
Vuilleumier, 2006	Switzerland	Prospective cohort	NR	INH 300 mg daily and vitamin B6 40 mg per day for a period of 6 months	Inclusion criteria: patients having LTBI as defined by the American Thoracic Society with normal plasma AST and ALT levels before the beginning of INH monotherapy Exclusion criteria: 1) a history of alcohol consumption, 2) positive serology for the HAV, HBV or HCV, 3) poor chemotherapy compliance (negative urine INH 3 times during the follow-up), loss during the follow-up, 4) patients had to receive supplementary anti-tuberculosis agents or other potentially hepatotoxic drugs	89	INH-induced hepatotoxicity
Wang, 2015 (GI: NTUH)	Taiwan	Prospective cohort	6 months	Daily INH, RMP, EMB and PZA in the first 2 months, and daily INH and RMP for the next 4 months The daily dosage of each drug was calculated by weight	Adult Taiwanese (>16 years) patients with culture-confirmed PTB Subjects were excluded if they were pregnant, had a life expectancy < 6 months, had abnormal baseline LFT or were resistant to INH, RMP or both	355 in the derivation cohort, 182 in the validation cohort	Hepatotoxicity during anti-tuberculosis treatment
Xiang, 2014	China	Prospective cohort	2 months	All patients were prescribed INH (600 mg), RMP (600 mg or 450 mg if the body weight was <50 kg), PZA (2000 mg) and EMB (1250 mg) every other day in the first 2 months. After 2 months, INH and RMP were continued for a further 4–6 months. Retreatment patients in addition received SM (750 mg) every other day in the first 2 months and continued receiving EMB for another 6 months	Inclusion criteria: newly diagnosed PTB patients belonging to the Uyghur ethnic group, who were receiving standard short-course chemotherapy recommended by the WHO, who attended for a 2-month assessment, and any patients attending clinic with suspected liver disease after the start of treatment, before the 2-month visit Exclusion criteria: patients who had signs of abnormal liver function when they started treatment (jaundice or elevated ALT, AST or bilirubin levels) or disease associated with liver dysfunction	2244	ATLI
Yamada, 2009 (GI: YAMADA)	Canada	Prospective cohort	9 months	INH 300 mg	Subjects who were receiving treatment with INH 300 mg daily self-administered for LTBI.Subjects were included if they were aged ⩾19 years; not concurrently receiving other anti-tuberculosis drugs; non-reactive to hepatitis B surface antigen and antibody to hepatitis C virus; absence of any liver or metabolic diseases; without a HIV-positive test result; not consuming ⩾7 alcoholic beverages per day and undergoing sufficiently frequentAST tests to detect hepatotoxicity	170	Hepatotoxicity
Yimer, 2011	Ethiopia	Prospective cohort	followed up for development of DILI for up to 56 weeks	All study participants received RMP based short-course chemotherapy for TB following the national TB treatment guideline. ART was then initiated	Newly diagnosed ART and anti-tuberculosis treatment-naïve adult TB and HIV co-infected patients. The eligibility criteria were age >18 years, CD4 count, 200 cells/Ul, not pregnant and not on other known hepatotoxic drugs concurrently (except cotrimoxazole, 960 mg per day, which was given to all participants before enrolment and during the follow-up period according to the treatment guideline)	353	Anti-tubercular and antiretroviral DILI
Yuliwulandari, 2016	Indonesia	Case-control	NR	NR (anti-tuberculosis treatment)	Patients included in the study were male and female aged 15–70 years, suffering from TB according to the WHO standards, and were under monitored treatment with anti-tuberculosis drugs Exclusion criteria: 1) history of liver disease such as: hepatitis A B or C; hepatoma; liver cirrhosis or cholelithiasis positive; 2) abnormal levels of any LFT (ALT, AST or total bilirubin) before anti-tuberculosis treatment	241	ATLI
Zaverucha-do-Valle, 2014	Brazil	Retrospective cohort	Follow-up at days 15 and 30 and at least one visit monthly until the end of treatment	600 mg/day of RMP, 400 mg/day of INH and 2 g/day of PZA for all patients with corporal weight >45 kg or adjusted for corporal weight <45 kg. After 2 months of treatment, PZA was discontinued	Inclusion criteria: signed written consent, sputum smear with AFB or culture positive for M. tuberculosis; ongoing TB treatment and laboratory tests of liver function Exclusion criteria: age <18 years, pregnancy and no >1 visit registered	131	ATDH

INH =isoniazid; RMP =rifampicin; PZA =pyrazinamide; EMB =ethambutol; ALT =alanine aminotransferase; AST =aspartate aminotransferase; RCT =randomised controlled trial; HIV =human immunodeficiency virus; SM =streptomycin; PTB =pulmonary tuberculosis; IU =international unit; ALP =alkaline phosphatase; DILI =drug-induced liver injury; LFT =liver function test; DIH =drug-induced hepatotoxicity; NR =not reported; ATDH =anti-tuberculosis drug-induced hepatotoxicity; GI =group identifier; ADR =adverse drug reaction; ULN =upper limit of normal; EPTB =extra-pulmonary TB; ADIH =anti-tuberculosis drug-induced hepatitis; ATD =anti-tuberculosis drug; MPE =maculopapular eruption; WHO = World Health Organization; gGTP = gamma-glutamyltranspeptidase; CMV = cytomegalovirus ; LTBI = latent tuberculous infection; ART =antiretroviral therapy; ATLI = anti-tuberculosis drug-induced liver injury.

**Table A.3:** (*continued*)

Author, year	Country	Study design	Follow-up time	Drugs and dosage	Selection criteria	Sample size *n*	Toxicity outcomes
An, 2012	China	App	6 months	Daily treatment with INH, RMP, PZA and EMB for 2 months, followed by 4 months treatment with INH and RMP, with drug dosages calculated according to body weight Body weight < 45 kg: RMP 300 mg, INH 200 mg, PZA 1000 mg Body weight of 45–55 kg: RMP 450 mg, INH 300 mg, PZA 1500 mg Body weight > 55 kg: RMP 600 mg, INH 400 mg, PZA 2000 mg	Inclusion criteria: Normal serum ALT, AST and bilirubin levels, no symptoms related to abnormal liver function (i.e., jaundice) before anti-tuberculosis drug treatment and close monitoring of changes in liver functions within 6 months of treatment Patients with and without hepatotoxicity during treatment Exclusion criteria: Malnutrition HIV type 1 infection Alcoholic liver disease or habitual drinking Hepatitis B or C infection, liver disease, systemic diseases and/or treatment with drugs other than the anti-tuberculosis drugs that can induce hepatotoxicity Severe TB or cardiac dysfunction that may cause liver dysfunction Transient increases in ALT	208	Hepatotoxocity
Azuma, 2013	Japan	RCT	6 months	All patients were treated with a 6-month regimen comprising INH, RMP, PZA and EMB/SM for the first 2 months, followed by RMP and INH for 4 months. All patients were started on the standard oral dose (~5 mg/kg body weight, once-daily). For pharmacogenetics-treatment patients, dosages were adjusted based on individual *NAT2* status within 3 days. Modified daily INH doses were respectively ~7.5, ~5 and ~2.5 mg/kg for rapid, intermediate and slow acetylators. Regarding the other drugs for the standard regimen, standard daily doses of RMP (10 mg/kg, maximum 600 mg/body), PZA (25 mg/kg, 1500 mg/body), EMB (15 mg/kg, 750 mg/body; 20 mg/kg, 1000 mg/body) and SM (15 mg/kg, 750 mg/body) were recommended with the following dose ranges allowed at the discretion of the physician in charge: RMP 8–12 mg/kg; PZA 20–30 mg/kg; EMB 15–20 mg/kg; SM 12–18 mg/kg	The eligible population was identified from a series of patients newly diagnosed with PTB (male and female aged 20–75 years), requiring the 6-month 4-drug standard treatment for the first time Exclusion criteria: abnormal test results for liver and kidney function (serum AST > 45 IU/l, ALT > 50 IU/l, ALP > 444 IU/l, total bilirubin > 1.6 mg/dl and creatinine > 1.4 mg/dl before the study treatments commenced, long-term use of steroids and/or immunosuppressants, inadequate clinical conditions such as hyperglycaemia, diabetes mellitus, acute life-threatening chronic progressive disease, pregnancy or lactation and alcoholism. Patients not expected to complete the study protocol for social reasons were not recruited	172	INH-DILI Peripheral neuropathy
Bose, 2011	India	Prospective cohort	Patients were followed up to 8 weeks after they had started with anti-tuberculosis treatment	All patients received anti-tuberculosis treatment (RMP, INH, EMB and PZA) according to body weight. All four drugs were given for 2 months. PZA and EMB were discontinued, while INH and RMP were continued for another 4 months RMP doses as follows: ⩽35 kg: 300 mg/day 36–50 kg: 450 mg/day >50 kg: 600 mg/day INH doses as follows: ⩽35 kg: 200 mg/day >35 kg: 400 mg/day PZA doses as follows: ⩽50 kg: 1.0 g/day >50 kg: 1.5 g/day EMB 20 mg/kg/day	Newly diagnosed patients of PTB. The baseline LFT of the patients was normal when they were started on anti-tuberculosis treatment Exclusion criteria: no history of habitual alcoholism, chronic liver diseases or steatosis	218	ADIH ADIH outcome
Çetintaş, 2008	Turkey	Prospective cohort	NR	Patients received INH 5 mg/kg (maximum 300 mg/day), RMP 10 mg/kg (maximum 600 mg/day), PZA 25 mg/kg (maximum 2000 mg/day), EMB 15–25 mg/kg (maximum 1500 mg/day)	Patients diagnosed with TB. Only patients with serum levels before initiation of treatment within the following ranges were included in the study: ALT 0–40 U/l, AST 5–45 U/l and total bilirubin 0.2–1.6 mg/dl Exclusion criteria: patients with positive serum hepatitis B surface antigen, hepatitis C antibody or hepatitis A immunoglobulin M antibody, and patients with alcoholic liver disease or any hepatic or systemic disease that could cause liver function disorder	100	DIH
Chamorro, 2013	Argentina	Prospective cohort	NR	The patients began a standard anti-tuberculosis treatment protocol for the first 2 months (INH 5 mg/kg/days, maximum 300 mg/day, RMP 10 mg/kg/day maximum 600 mg/day, PZA 20 mg/kg/day, EMB 20 mg/kg/day), followed by INH and RMP for ⩾4 months, depending on the disease severity or the presence ofextra-pulmonary foci	Inclusion criteria: TB patients aged >18 years with stable haemodynamic levels, normal renal function and negative for pregnancy Exclusion criteria: presence of diseases that directly affected the liver (acute hepatitis A, active hepatitis B and C; cirrhosis; encephalopathy and cancer), INH allergy, HIV, autoimmunity, concomitant hepatotoxic medications, a history of TB treatment failures, refusal of blood extraction and refusal to sign the written informed consent	175	ATDH
Chang, 2012	Taiwan	Prospective cohort	NR	First-line anti-tuberculosis medications	Patients diagnosed with TB and treated with first-line anti-tuberculosis medications Exclusion criteria: pregnancy, abnormal liver function and history of positive viral hepatitis before starting treatment with first-line anti-tuberculosis drugs	98	ATDH
Cho, 2007	Korea	Prospective cohort	Serum AST, ALT and total bilirubin levels were then monitored monthly until the end of treatment	All patients received oral INH (300 mg), RMP (600 mg), PZA (20 mg/kg body weight) and EMB (800 mg) daily for the first 2 months. PZA was then discontinued, while INH, RMP and EMB were continued for another 4 months	Adult patients newly diagnosed with active TB, with evident lesion of TB using simple X-ray or computed tomography or positive results on sputum smear or culture for detection of mycobacteria Exclusion criteria: 1) abnormal serum ALT, AST, or bilirubin levels or symptoms related to abnormal liver function such as jaundice before anti-tuberculosis treatment; 2) alcoholic liver disease or habitual alcohol drinking; 3) any other hepatic or systemic diseases that may cause liver dysfunction	132	ATDH
Costa, 2012	Brazil	Prospective cohort	NR	All patients were treated with the first-line anti-tuberculosis drug regimen INH (300 mg/kg/day), RMP (300 mg/kg/day) and PZA (1500 mg/kg/day) for the first 2 months, followed by INH and RMP for a further 4 months	Male or female subjects aged ⩾18 years, with no previously described renal, allergic or hepatic diseases and not pregnant	129	ADRs
Dhoro, 2013	Zimbabwe	Case-control	NR	NR	TB patients	Not clear—189 received INH	Peripheral neuropathy
Feng, 2014	China	Case-control	6 months	Treatment with anti-tuberculosis drug regimens at the usual dosage—300 mg/day INH, 450 mg/day RMP and 1500 mg/day PZA	Selection of cases: cases selected based on liver functions, i.e., all indices of liver function were normal before anti-tuberculosis chemotherapy, and became abnormal indicating hepatic injury after 6 months of chemotherapy. Cases were patients who showed ATDH based on increased serum transaminase values that were three-fold higher than the ULN (40 IU/l ALT) and symptoms compatible with hepatitis Selection of controls: controls underwent the same anti-tuberculosis chemotherapy with selected cases and were not tested for abnormality in liver functions after 6 months of the chemotherapy. The controls selected matched the criteria compared to the cases: 1) sex; 2) age discrepancy of <5 years; 3) living in the same regions; and 4) treatment with anti-tuberculosis drug regimens at the usual dosage, including 300 mg/day INH, 450 mg/day RMP and 1500 mg/day PZA	346	ATDH
Fredj, 2016	Tunisia	Prospective cohort	Serum AST, ALT and ALP were monitored monthly until the end of the treatment	The anti-tuberculosis treatment was based on the association of INH (5 mg/kg/day), RMP (10 mg/kg/day), PZA (25 mg/kg/day) and EMB (15 mg/kg/day) for the first 2 months, followed by INH and RMP for 4–7 additional months, depending on TB clinical presentation	Patients diagnosed with PTB and EPTB	71	INH-induced-hepatotoxicity
Gupta, 2013 (GI: GUPTA)	India	Prospective cohort	The patients were monitored for ALT, AST, andtotal bilirubin levels weekly for 1 month and then monthly until the completion of treatment	Initially patients received a combination regimen including INH 5 mg/kg (max 300 mg daily), RMP 10 mg/kg (max 600 mg daily), PZA 25 mg/kg (max 1500 mg daily) and EMB 15–25 mg/kg (max 2000 mg daily) for a period of 2 months, followed by an additional 4 months with INH and RMP	Inclusion criteria: 1) age > 18 years; 2) smear and/or culture positive for mycobacteria in clinical samples; and 3) normal ALT, AST and total bilirubin levels Exclusion criteria: 1) patients presenting clinically and laboratory-confirmed chronic liver disease such as jaundice; 2) acute and chronic hepatitis B and/or C or HIV; 3) alcoholic liver diseases; 4) a rise of two times the ULN of ALT, AST and total bilirubin levels; 5) medication with anti-tuberculosis drugs before start of treatment and/or other potentially hepatotoxic drugs; and 6) refusal to provide blood sample or signed informed consent form	215	ATDH
Higuchi, 2007 (GI: HIGUCHI)	Japan	Prospective cohort	NR	Treated with a INH (400 mg/d) and RMP (450 mg/d) containing regimen for 6 or 9 months	Patients with new onset of PTB treated with a INH (400 mg/d) and RMP (450 mg/d) containing regimen for 6 or 9 months Exclusion criteria: patients with liver cirrhosis, chronic and acute hepatitis, alcoholic liver disease and other chronic liver diseases	100	ATDH Skin rash Eosinophilia
Ho, 2013	Taiwan	Prospective cohort	180 days	All patients received oral INH 300 mg, RMP 600 mg (or 450 mg if body weight was <50 kg), PZA 25 mg/kg of body weight (max daily dose 2000 mg) and EMB 15 mg/kg of body weight daily (max daily dose 1600 mg) for the first 2 months. PZA was then discontinued, whereas INH, RMP and EMB were continued for another 4 months	The inclusion criteria included a sputum smear with AFB or culture positive for M. tuberculosis, undergoing anti-tuberculosis treatment, including INH or starting treatment during the recruitment period, hepatitis serology recorded in the medical chart with signed written consent Patients were excluded if they were infected with hepatitis B virus, hepatitis C virus or HIV, had any other hepatic disease, or did not have at least two visits documented in the medical records	348	ATDH
Huang, 2003 (GI: HUANG)	Taiwan	Prospective cohort	Serum ALT, AST and total bilirubin levels were monitored monthly until the end of treatment or checked whenever patients had symptoms of suspected hepatitis	Their standard daily anti-tuberculosis regimen for the first 2 months included INH (300 mg), RMP (600 mg or 450 mg if body weight <50 kg), PZA (20 mg/kg body weight) and EMB (25 mg/kg body weight). PZA was then discontinued, whereas INH, RMP, and EMB (15 mg/kg body weight) were continued for another 4 months	Patients with incident PTB or EPTB Exclusion criteria: 1) abnormal serum ALT, AST or bilirubin before anti-tuberculosis treatment; and 2) refusal of blood sampling or informed written consent	318	ADIH
Jung, 2015	Korea	Prospective cohort	4 weeks	Standard 4-drug treatment for 6 months: INH (5 mg/kg, usually 300 mg), RMP (450 mg for <50 kg or 600 mg for ⩾50 kg of body weight), EMB (15 mg/kg) and PZA (20–30 mg/kg), daily for 2 months, followed by INH and RMP with or without EMB for 4 months. Doses were adjusted when applying model-based treatment	Eligible participants were patients newly diagnosed with active TB, who underwent standard 4-drug treatment for 6 months Exclusion criteria: patients with abnormal hepatic function on laboratory testing (increased serum AST, ALT or total bilirubin) before anti-tuberculosis treatment and underlying liver disease or systemic illness such as congestive heart failure, acute life-threatening disease, or alcoholism or disease that was resistant to INH at the start of treatment	206	ATDH
Khalili, 2011	Iran	Case-control	2 months	Treated daily with INH (300 mg), RMP (600 mg), PZA (20 mg/kg), EMB (15 mg/kg) for the first 2 months, followed by INH and RMP daily for 4 additional months	Inclusion criteria: newly diagnosed patients (>18 years) with active PTB, who had been planned to be treated daily with anti-tuberculosis treatment (see drugs and dosage) Exclusion criteria: other possibilities of hepatitis, including patients with HIV infection, hepatic insufficiency (ALT or AST > 2× ULN) or clinically signs and symptoms of liver diseases such as jaundice and ascites, chronic hepatitis (B or C) and renal insufficiency (creatinine clearance < 50 ml/min based on Cockcroft-Gault equation) and history or current use of herbal, supplemental and hepatotoxic non-tuberculosis drugs	100	Hepatotoxicity
Kim, 2009 (GI: KIM)	Korea	Case-control	Assessments performed 2 weeks after onset of treatment and bi-monthly thereafter	All patients with PTB were treated daily with a combination regimen comprising INH (300–400 mg daily), RMP (450–600 mg daily), EMB (600–800 mg daily) and PZA (1000–1500 mg daily) for 2 months and then without PZA for ⩾4 following months. Doses of each drug were adjusted based on body weight of the patient	Newly diagnosed and treated patients with PTB. Exclusion criteria: patients with active or chronic hepatitis, including alcoholic hepatitis, fatty liver disease, liver cirrhosis, carriers of the hepatitis B or C virus, heavy alcohol intake, decreased renal function and severe cardiac diseases requiring several medications	226	ATDH
Kim, 2011 (GI: KIM)	Korea	Case-control	NR	The treatment comprised an initial phase of 2 months and a subsequent continuation phase of ⩾4 months. During the initial phase, 4 drugs were administered, including INH (300–400 mg daily), RMP (450–600 mg daily), EMB (600–800 mg daily) and PZA (1000–1500 mg daily). Doses of each drug were adjusted based on the body weight of the patient. In the following continuation phase, only PZA was discontinued, while the other three drugs were continued	Patients newly diagnosed with PTB and/or TB pleuritis and treated with first-line anti-tuberculosis medications such as INH, RMP, EMB and PZA Exclusion criteria: 1) patients with skin diseases before treatment, 2) chronic renal failure and chronic liver diseases affecting drug metabolism, 3) chronic alcoholism, 4) other chronic medical conditions requiring medication, and 5) non-adherence to treatment	221	ATD-induced MPE
Lee, 2010	Taiwan	Prospective cohort	NR	All patients received oral INH 300 mg, RMP 600 mg (or 450 mg, if body weight was <50 kg), PZA 200 mg/kg of body weight and EMB 800 mg daily for the first 2 months. PZA was then discontinued, while INH, RMP and EMB were continued for another 4 months	Inclusion criteria: adult patients newly diagnosed with active TB, having evident lesions of TB using simple X-ray, computed tomography, sputum smears and cultures positive for mycobacteria Exclusion criteria: 1) positive serum hepatitis B virus surface antigen, antibody to hepatitis C virus; 2) alcoholic liver disease or habitual alcohol drinking; 3) any other hepatic or systemic diseases that may cause liver dysfunction; 4) abnormal serum ALT, AST or bilirubin levels before anti-tuberculosis treatment	140	ATDH
Leiro-Fernandez, 2011	Spain	Case-control	Routine follow-up of clinical assessments every 2 weeks during the first month and monthly thereafter untilcompletion of treatment	Treatment with regimens that included INH, RMP and PZA at the usual dosages (INH 5 mg/kg/day to maximum 300 mg/day, RMP10 mg/kg/day to maximum 600 mg/day and PZA 25–30 mg/kg/day to maximum 2500 mg/day	Inclusion criteria: 1) age 15–75 years; 2) microbiological demonstration of active TB; 3) treatment with regimens that included INH, RMP and PZA at the usual dosages (INH 5 mg/kg/day to max 300 mg/day, RMP 10 mg/kg/day to max 600 mg/day and PZA 25–30 mg/kg/day to max 2500 mg/day); and 4) adequate treatment adherence Exclusion criteria: 1) increased baseline serum transaminases (AST and/or ALT; normal values ⩽ 40 IU/l); 2) positive serological testing for HIV, hepatitis B virus or hepatitis C virus; 3) regular alcohol intake or concomitant use of hepatotoxic drugs; 4) history of chronic liver disease; 5) pregnancy; or 6) no or poor adherence to treatment	117	ATDH
Lv, 2012	China	Case-control	6–9 months	All primary/retreatment patients took INH (600 mg), RMP (600 mg or 450 mg if body weight was <50 kg), PZA (2000 mg) and EMB (1250 mg) every other day in the first 2 months and then INH and RMP were continued for another 4/6 months. The retreatment patients received SM (750 mg) every other day in the first 2 months and continued receiving EMB for another 6 months	Inclusion criteria: sputum smear-positive PTB patients who received standard short-course chemotherapy recommended by the WHO Exclusion criteria: 1) positive serum hepatitis B virus surface antigen or other liver disease, 2) potentially hepatotoxic medications that would confound the picture, 3) abnormal serum ALT, AST or total bilirubin levels before anti-tuberculosis treatment	445	ATDH
Mahmoud, 2012	Tunisia	Prospective cohort	3 months	Treated with INH and RMP containing regimen	Inclusion criteria: adult patients (>18 years) newly diagnosed with TB treated with INH- and RMP-containing regimen, LFTs before initiation of treatment showed completely normal findings on serum ALT, AST, total bilirubin, ALP and gGTP Exclusion criteria: 1) patients receiving other potentially hepatotoxic drugs in addition to anti-tuberculosis agents; 2) positive serum hepatitis B surface antigen or hepatitis C antibody; 3) alcoholic liver disease; 4) any other hepatic or systemic diseases that may cause liver dysfunction	66	INH hepatotoxicity
Ng, 2014	Cases recruited in the UK; controls were samples from elsewhere	Case-control	NR	All had been prescribed INH with all but one also taking additional anti-tuberculosis drugs. Patients were prescribed 300 mg INH/day in line with UK/WHO guidelines	Cases: patients diagnosed with DILI either in the past or at the time of sample collection. Onlycases assessed as having DILI that was highly probably, probably or possibly relating to anti-tuberculosis drug exposure were enrolled in the study. Other possible causes of liver toxicity, particularly hepatitis A, B, C and CMV infection were excluded. No patients included were HIV-positive or had pre-existing liver disease. None had been treated for TB under directly observed treatment Controls: 52 DNA samples from individuals of European ancestry; inclusion/exclusion criteria not reported	127	DILI
Ohno, 2000	Japan	Prospective cohort	3 months	Initial chemotherapy always included INH (400 mg/day; 8.2 ± 2.0 mg/kg/day) and RMP (450 mg/day; 9.2 ± 2.2 mg/kg/day); the third drug used was EMB or SM.	Inclusion criteria: patients had no history of alcohol abuse and were negative for hepatitis B surface antigen and hepatitis C antibody. At the beginning of treatment, LFTs showed completely normal findings on serum AST, ALT, bilirubin, ALP and gGTP Patients receiving other potentially hepatopathic drugs in addition to anti-tuberculosis agents were excluded from the study	77	INH + RMP-induced hepatotoxicity
Possuelo, 2008 (GI: POSSUELO)	Brazil	Prospective cohort	NR	Treatment daily with INH, RMP and PZA for the first 2 months followed by INH and RMP daily for 4 additional months. Drug dosages used were calculated according to patient's weight (weight <45 kg: RMP 300 mg, INH 200 mg, PZA 1000 mg; 45–55 kg: RMP 450 mg, INH 300 mg, PZA 1500 mg; >55 kg: RMP 600 mg, INH 400 mg, PZA 2000 mg)	Inclusion criteria: adult patients (>18 years) newly diagnosed with active TB, who had been treated daily with anti-tuberculosis treatment (see drugs and dosage) Exclusion criteria: patients presenting clinically and laboratory-confirmed liver chronic disease, patients using anti-tuberculosis drugs before enrolment in the study, patients presenting results of LFTs before the beginning of treatment higher that were 2× ULN and refusal to participate of the study	254	ATDH Gastrointestinal ADRs
Rana, 2014 (GI: RANA)	India	Prospective cohort	Patients were monitored every month until the end of treatment or whenever the patients had symptoms or signs of hepatotoxicity	Daily anti-tuberculosis treatment for the first 2 months included INH (300 mg), RMP (600 or 450 mg for body weight/50 kg), PZA (20 mg/kg body weight) and EMB (25 mg/kg body weight). After 2 months, EMB and PZA were discontinued, whereas INH and RMP were continued for an additional 4 months	Inclusion criteria: patients with pulmonary and EPTB Exclusion criteria: patients with history of alcohol abuse and/or any other liver disease; patients who had received other potentially hepatotoxic drugs in addition to anti-tuberculosis drugs; patients who had abnormal serum ALT, AST or bilirubin before starting anti-tuberculosis treatment; patientswho developed viral hepatitis during anti-tuberculosis treatment; patients with renal failure or cancer; and patients who refused blood sampling or providing informed written consent	300	Hepatotoxicity
Santos, 2013 (GI: SANTOS)	Brazil	Prospective cohort	NR	Treatment with INH, RMP and PZA for the first 2 months, followed by INH and RMP daily for 4 months	Inclusion criteria: patients diagnosed with TB and treated with anti-tuberculosis treatment (see drugs and dosage) Exclusion criteria: patients aged <18 years, those with mental disabilities, chronic liver disease confirmed by clinical and laboratory data, users of anti-tuberculosis drugs before enrolment in the study, and those with liver function results > 2× ULN before beginning treatment	270	ATDH
Shimizu, 2006	Japan	Prospective cohort	3 months	Treatment with INH (300–400 mg) and RMP (300–450 mg)	Individuals with PTB without abnormal serum levels of ALT, AST, direct or total bilirubin, or serum hepatitis B virus surface antigen or antibody to hepatitis C virus at the beginning of the study	42	Hepatotoxicity
Singla, 2014	India	Prospective cohort	NR	NR	Inclusion criteria: newly diagnosed patients with TB Exclusion criteria: history of heavy use of alcohol or chronic liver diseases or liver cirrhosis;patients who were settled in the area for a minimum of three generations; people infected with HIV	408	ATDH
Sotsuka, 2011	Japan	Prospective cohort	3 months	INH, RMP and PZA, plus EMB or SM during the first 2 months, followed by administration of INH and RMP plus EMB or SM during the final 4 months	Inclusion criteria: in-patients with active PTB who were treated with the standard Japanese chemotherapy regimen, followed up for >3 months after treatment, and who consented to this study	144	Hepatotoxicity
Teixeira, 2011	Brazil	Case-control	NR	Anti-tuberculosis drug regimens that include INH at the usual dosage (400 mg/day)	Inclusion criteria: 1) age > 18 years, 2) diagnosis of active TB, 3) treatment with anti-tuberculosis drug regimens that include INH at the usual dosage (400 mg/day) and 4) normal baseline serum transaminases (ALT and AST) before treatment Exclusion criteria: 1) positive serological test for the HIV, hepatitis B virus or hepatitis C virus, 2) alcohol abuse, 3) history of chronic liver disease and 4) pregnancy	167	Hepatitis
Vuilleumier, 2006	Switzerland	Prospective cohort	NR	INH 300 mg daily and vitamin B6 40 mg per day for a period of 6 months	Inclusion criteria: patients having LTBI as defined by the American Thoracic Society with normal plasma AST and ALT levels before the beginning of INH monotherapy Exclusion criteria: 1) a history of alcohol consumption, 2) positive serology for the HAV, HBV or HCV, 3) poor chemotherapy compliance (negative urine INH 3 times during the follow-up), loss during the follow-up, 4) patients had to receive supplementary anti-tuberculosis agents or other potentially hepatotoxic drugs	89	INH-induced hepatotoxicity
Wang, 2015 (GI: NTUH)	Taiwan	Prospective cohort	6 months	Daily INH, RMP, EMB and PZA in the first 2 months, and daily INH and RMP for the next 4 months The daily dosage of each drug was calculated by weight	Adult Taiwanese (>16 years) patients with culture-confirmed PTB Subjects were excluded if they were pregnant, had a life expectancy < 6 months, had abnormal baseline LFT or were resistant to INH, RMP or both	355 in the derivation cohort, 182 in the validation cohort	Hepatotoxicity during anti-tuberculosis treatment
Xiang, 2014	China	Prospective cohort	2 months	All patients were prescribed INH (600 mg), RMP (600 mg or 450 mg if the body weight was <50 kg), PZA (2000 mg) and EMB (1250 mg) every other day in the first 2 months. After 2 months, INH and RMP were continued for a further 4–6 months. Retreatment patients in addition received SM (750 mg) every other day in the first 2 months and continued receiving EMB for another 6 months	Inclusion criteria: newly diagnosed PTB patients belonging to the Uyghur ethnic group, who were receiving standard short-course chemotherapy recommended by the WHO, who attended for a 2-month assessment, and any patients attending clinic with suspected liver disease after the start of treatment, before the 2-month visit Exclusion criteria: patients who had signs of abnormal liver function when they started treatment (jaundice or elevated ALT, AST or bilirubin levels) or disease associated with liver dysfunction	2244	ATLI
Yamada, 2009 (GI: YAMADA)	Canada	Prospective cohort	9 months	INH 300 mg	Subjects who were receiving treatment with INH 300 mg daily self-administered for LTBI.Subjects were included if they were aged ⩾19 years; not concurrently receiving other anti-tuberculosis drugs; non-reactive to hepatitis B surface antigen and antibody to hepatitis C virus; absence of any liver or metabolic diseases; without a HIV-positive test result; not consuming ⩾7 alcoholic beverages per day and undergoing sufficiently frequentAST tests to detect hepatotoxicity	170	Hepatotoxicity
Yimer, 2011	Ethiopia	Prospective cohort	followed up for development of DILI for up to 56 weeks	All study participants received RMP based short-course chemotherapy for TB following the national TB treatment guideline. ART was then initiated	Newly diagnosed ART and anti-tuberculosis treatment-naïve adult TB and HIV co-infected patients. The eligibility criteria were age >18 years, CD4 count, 200 cells/Ul, not pregnant and not on other known hepatotoxic drugs concurrently (except cotrimoxazole, 960 mg per day, which was given to all participants before enrolment and during the follow-up period according to the treatment guideline)	353	Anti-tubercular and antiretroviral DILI
Yuliwulandari, 2016	Indonesia	Case-control	NR	NR (anti-tuberculosis treatment)	Patients included in the study were male and female aged 15–70 years, suffering from TB according to the WHO standards, and were under monitored treatment with anti-tuberculosis drugs Exclusion criteria: 1) history of liver disease such as: hepatitis A B or C; hepatoma; liver cirrhosis or cholelithiasis positive; 2) abnormal levels of any LFT (ALT, AST or total bilirubin) before anti-tuberculosis treatment	241	ATLI
Zaverucha-do-Valle, 2014	Brazil	Retrospective cohort	Follow-up at days 15 and 30 and at least one visit monthly until the end of treatment	600 mg/day of RMP, 400 mg/day of INH and 2 g/day of PZA for all patients with corporal weight >45 kg or adjusted for corporal weight <45 kg. After 2 months of treatment, PZA was discontinued	Inclusion criteria: signed written consent, sputum smear with AFB or culture positive for M. tuberculosis; ongoing TB treatment and laboratory tests of liver function Exclusion criteria: age <18 years, pregnancy and no >1 visit registered	131	ATDH

INH =isoniazid; RMP =rifampicin; PZA =pyrazinamide; EMB =ethambutol; ALT =alanine aminotransferase; AST =aspartate aminotransferase; RCT =randomised controlled trial; HIV =human immunodeficiency virus; SM =streptomycin; PTB =pulmonary tuberculosis; IU =international unit; ALP =alkaline phosphatase; DILI =drug-induced liver injury; LFT =liver function test; DIH =drug-induced hepatotoxicity; NR =not reported; ATDH =anti-tuberculosis drug-induced hepatotoxicity; GI =group identifier; ADR =adverse drug reaction; ULN =upper limit of normal; EPTB =extra-pulmonary TB; ADIH =anti-tuberculosis drug-induced hepatitis; ATD =anti-tuberculosis drug; MPE =maculopapular eruption; WHO = World Health Organization; gGTP = gamma-glutamyltranspeptidase; CMV = cytomegalovirus ; LTBI = latent tuberculous infection; ART =antiretroviral therapy; ATLI = anti-tuberculosis drug-induced liver injury.

**Table A.3:** (*continued*)

Author, year	Country	Study design	Follow-up time	Drugs and dosage	Selection criteria	Sample size *n*	Toxicity outcomes
An, 2012	China	App	6 months	Daily treatment with INH, RMP, PZA and EMB for 2 months, followed by 4 months treatment with INH and RMP, with drug dosages calculated according to body weight Body weight < 45 kg: RMP 300 mg, INH 200 mg, PZA 1000 mg Body weight of 45–55 kg: RMP 450 mg, INH 300 mg, PZA 1500 mg Body weight > 55 kg: RMP 600 mg, INH 400 mg, PZA 2000 mg	Inclusion criteria: Normal serum ALT, AST and bilirubin levels, no symptoms related to abnormal liver function (i.e., jaundice) before anti-tuberculosis drug treatment and close monitoring of changes in liver functions within 6 months of treatment Patients with and without hepatotoxicity during treatment Exclusion criteria: Malnutrition HIV type 1 infection Alcoholic liver disease or habitual drinking Hepatitis B or C infection, liver disease, systemic diseases and/or treatment with drugs other than the anti-tuberculosis drugs that can induce hepatotoxicity Severe TB or cardiac dysfunction that may cause liver dysfunction Transient increases in ALT	208	Hepatotoxocity
Azuma, 2013	Japan	RCT	6 months	All patients were treated with a 6-month regimen comprising INH, RMP, PZA and EMB/SM for the first 2 months, followed by RMP and INH for 4 months. All patients were started on the standard oral dose (~5 mg/kg body weight, once-daily). For pharmacogenetics-treatment patients, dosages were adjusted based on individual *NAT2* status within 3 days. Modified daily INH doses were respectively ~7.5, ~5 and ~2.5 mg/kg for rapid, intermediate and slow acetylators. Regarding the other drugs for the standard regimen, standard daily doses of RMP (10 mg/kg, maximum 600 mg/body), PZA (25 mg/kg, 1500 mg/body), EMB (15 mg/kg, 750 mg/body; 20 mg/kg, 1000 mg/body) and SM (15 mg/kg, 750 mg/body) were recommended with the following dose ranges allowed at the discretion of the physician in charge: RMP 8–12 mg/kg; PZA 20–30 mg/kg; EMB 15–20 mg/kg; SM 12–18 mg/kg	The eligible population was identified from a series of patients newly diagnosed with PTB (male and female aged 20–75 years), requiring the 6-month 4-drug standard treatment for the first time Exclusion criteria: abnormal test results for liver and kidney function (serum AST > 45 IU/l, ALT > 50 IU/l, ALP > 444 IU/l, total bilirubin > 1.6 mg/dl and creatinine > 1.4 mg/dl before the study treatments commenced, long-term use of steroids and/or immunosuppressants, inadequate clinical conditions such as hyperglycaemia, diabetes mellitus, acute life-threatening chronic progressive disease, pregnancy or lactation and alcoholism. Patients not expected to complete the study protocol for social reasons were not recruited	172	INH-DILI Peripheral neuropathy
Bose, 2011	India	Prospective cohort	Patients were followed up to 8 weeks after they had started with anti-tuberculosis treatment	All patients received anti-tuberculosis treatment (RMP, INH, EMB and PZA) according to body weight. All four drugs were given for 2 months. PZA and EMB were discontinued, while INH and RMP were continued for another 4 months RMP doses as follows: ⩽35 kg: 300 mg/day 36–50 kg: 450 mg/day >50 kg: 600 mg/day INH doses as follows: ⩽35 kg: 200 mg/day >35 kg: 400 mg/day PZA doses as follows: ⩽50 kg: 1.0 g/day >50 kg: 1.5 g/day EMB 20 mg/kg/day	Newly diagnosed patients of PTB. The baseline LFT of the patients was normal when they were started on anti-tuberculosis treatment Exclusion criteria: no history of habitual alcoholism, chronic liver diseases or steatosis	218	ADIH ADIH outcome
Çetintaş, 2008	Turkey	Prospective cohort	NR	Patients received INH 5 mg/kg (maximum 300 mg/day), RMP 10 mg/kg (maximum 600 mg/day), PZA 25 mg/kg (maximum 2000 mg/day), EMB 15–25 mg/kg (maximum 1500 mg/day)	Patients diagnosed with TB. Only patients with serum levels before initiation of treatment within the following ranges were included in the study: ALT 0–40 U/l, AST 5–45 U/l and total bilirubin 0.2–1.6 mg/dl Exclusion criteria: patients with positive serum hepatitis B surface antigen, hepatitis C antibody or hepatitis A immunoglobulin M antibody, and patients with alcoholic liver disease or any hepatic or systemic disease that could cause liver function disorder	100	DIH
Chamorro, 2013	Argentina	Prospective cohort	NR	The patients began a standard anti-tuberculosis treatment protocol for the first 2 months (INH 5 mg/kg/days, maximum 300 mg/day, RMP 10 mg/kg/day maximum 600 mg/day, PZA 20 mg/kg/day, EMB 20 mg/kg/day), followed by INH and RMP for ⩾4 months, depending on the disease severity or the presence ofextra-pulmonary foci	Inclusion criteria: TB patients aged >18 years with stable haemodynamic levels, normal renal function and negative for pregnancy Exclusion criteria: presence of diseases that directly affected the liver (acute hepatitis A, active hepatitis B and C; cirrhosis; encephalopathy and cancer), INH allergy, HIV, autoimmunity, concomitant hepatotoxic medications, a history of TB treatment failures, refusal of blood extraction and refusal to sign the written informed consent	175	ATDH
Chang, 2012	Taiwan	Prospective cohort	NR	First-line anti-tuberculosis medications	Patients diagnosed with TB and treated with first-line anti-tuberculosis medications Exclusion criteria: pregnancy, abnormal liver function and history of positive viral hepatitis before starting treatment with first-line anti-tuberculosis drugs	98	ATDH
Cho, 2007	Korea	Prospective cohort	Serum AST, ALT and total bilirubin levels were then monitored monthly until the end of treatment	All patients received oral INH (300 mg), RMP (600 mg), PZA (20 mg/kg body weight) and EMB (800 mg) daily for the first 2 months. PZA was then discontinued, while INH, RMP and EMB were continued for another 4 months	Adult patients newly diagnosed with active TB, with evident lesion of TB using simple X-ray or computed tomography or positive results on sputum smear or culture for detection of mycobacteria Exclusion criteria: 1) abnormal serum ALT, AST, or bilirubin levels or symptoms related to abnormal liver function such as jaundice before anti-tuberculosis treatment; 2) alcoholic liver disease or habitual alcohol drinking; 3) any other hepatic or systemic diseases that may cause liver dysfunction	132	ATDH
Costa, 2012	Brazil	Prospective cohort	NR	All patients were treated with the first-line anti-tuberculosis drug regimen INH (300 mg/kg/day), RMP (300 mg/kg/day) and PZA (1500 mg/kg/day) for the first 2 months, followed by INH and RMP for a further 4 months	Male or female subjects aged ⩾18 years, with no previously described renal, allergic or hepatic diseases and not pregnant	129	ADRs
Dhoro, 2013	Zimbabwe	Case-control	NR	NR	TB patients	Not clear—189 received INH	Peripheral neuropathy
Feng, 2014	China	Case-control	6 months	Treatment with anti-tuberculosis drug regimens at the usual dosage—300 mg/day INH, 450 mg/day RMP and 1500 mg/day PZA	Selection of cases: cases selected based on liver functions, i.e., all indices of liver function were normal before anti-tuberculosis chemotherapy, and became abnormal indicating hepatic injury after 6 months of chemotherapy. Cases were patients who showed ATDH based on increased serum transaminase values that were three-fold higher than the ULN (40 IU/l ALT) and symptoms compatible with hepatitis Selection of controls: controls underwent the same anti-tuberculosis chemotherapy with selected cases and were not tested for abnormality in liver functions after 6 months of the chemotherapy. The controls selected matched the criteria compared to the cases: 1) sex; 2) age discrepancy of <5 years; 3) living in the same regions; and 4) treatment with anti-tuberculosis drug regimens at the usual dosage, including 300 mg/day INH, 450 mg/day RMP and 1500 mg/day PZA	346	ATDH
Fredj, 2016	Tunisia	Prospective cohort	Serum AST, ALT and ALP were monitored monthly until the end of the treatment	The anti-tuberculosis treatment was based on the association of INH (5 mg/kg/day), RMP (10 mg/kg/day), PZA (25 mg/kg/day) and EMB (15 mg/kg/day) for the first 2 months, followed by INH and RMP for 4–7 additional months, depending on TB clinical presentation	Patients diagnosed with PTB and EPTB	71	INH-induced-hepatotoxicity
Gupta, 2013 (GI: GUPTA)	India	Prospective cohort	The patients were monitored for ALT, AST, andtotal bilirubin levels weekly for 1 month and then monthly until the completion of treatment	Initially patients received a combination regimen including INH 5 mg/kg (max 300 mg daily), RMP 10 mg/kg (max 600 mg daily), PZA 25 mg/kg (max 1500 mg daily) and EMB 15–25 mg/kg (max 2000 mg daily) for a period of 2 months, followed by an additional 4 months with INH and RMP	Inclusion criteria: 1) age > 18 years; 2) smear and/or culture positive for mycobacteria in clinical samples; and 3) normal ALT, AST and total bilirubin levels Exclusion criteria: 1) patients presenting clinically and laboratory-confirmed chronic liver disease such as jaundice; 2) acute and chronic hepatitis B and/or C or HIV; 3) alcoholic liver diseases; 4) a rise of two times the ULN of ALT, AST and total bilirubin levels; 5) medication with anti-tuberculosis drugs before start of treatment and/or other potentially hepatotoxic drugs; and 6) refusal to provide blood sample or signed informed consent form	215	ATDH
Higuchi, 2007 (GI: HIGUCHI)	Japan	Prospective cohort	NR	Treated with a INH (400 mg/d) and RMP (450 mg/d) containing regimen for 6 or 9 months	Patients with new onset of PTB treated with a INH (400 mg/d) and RMP (450 mg/d) containing regimen for 6 or 9 months Exclusion criteria: patients with liver cirrhosis, chronic and acute hepatitis, alcoholic liver disease and other chronic liver diseases	100	ATDH Skin rash Eosinophilia
Ho, 2013	Taiwan	Prospective cohort	180 days	All patients received oral INH 300 mg, RMP 600 mg (or 450 mg if body weight was <50 kg), PZA 25 mg/kg of body weight (max daily dose 2000 mg) and EMB 15 mg/kg of body weight daily (max daily dose 1600 mg) for the first 2 months. PZA was then discontinued, whereas INH, RMP and EMB were continued for another 4 months	The inclusion criteria included a sputum smear with AFB or culture positive for M. tuberculosis, undergoing anti-tuberculosis treatment, including INH or starting treatment during the recruitment period, hepatitis serology recorded in the medical chart with signed written consent Patients were excluded if they were infected with hepatitis B virus, hepatitis C virus or HIV, had any other hepatic disease, or did not have at least two visits documented in the medical records	348	ATDH
Huang, 2003 (GI: HUANG)	Taiwan	Prospective cohort	Serum ALT, AST and total bilirubin levels were monitored monthly until the end of treatment or checked whenever patients had symptoms of suspected hepatitis	Their standard daily anti-tuberculosis regimen for the first 2 months included INH (300 mg), RMP (600 mg or 450 mg if body weight <50 kg), PZA (20 mg/kg body weight) and EMB (25 mg/kg body weight). PZA was then discontinued, whereas INH, RMP, and EMB (15 mg/kg body weight) were continued for another 4 months	Patients with incident PTB or EPTB Exclusion criteria: 1) abnormal serum ALT, AST or bilirubin before anti-tuberculosis treatment; and 2) refusal of blood sampling or informed written consent	318	ADIH
Jung, 2015	Korea	Prospective cohort	4 weeks	Standard 4-drug treatment for 6 months: INH (5 mg/kg, usually 300 mg), RMP (450 mg for <50 kg or 600 mg for ⩾50 kg of body weight), EMB (15 mg/kg) and PZA (20–30 mg/kg), daily for 2 months, followed by INH and RMP with or without EMB for 4 months. Doses were adjusted when applying model-based treatment	Eligible participants were patients newly diagnosed with active TB, who underwent standard 4-drug treatment for 6 months Exclusion criteria: patients with abnormal hepatic function on laboratory testing (increased serum AST, ALT or total bilirubin) before anti-tuberculosis treatment and underlying liver disease or systemic illness such as congestive heart failure, acute life-threatening disease, or alcoholism or disease that was resistant to INH at the start of treatment	206	ATDH
Khalili, 2011	Iran	Case-control	2 months	Treated daily with INH (300 mg), RMP (600 mg), PZA (20 mg/kg), EMB (15 mg/kg) for the first 2 months, followed by INH and RMP daily for 4 additional months	Inclusion criteria: newly diagnosed patients (>18 years) with active PTB, who had been planned to be treated daily with anti-tuberculosis treatment (see drugs and dosage) Exclusion criteria: other possibilities of hepatitis, including patients with HIV infection, hepatic insufficiency (ALT or AST > 2× ULN) or clinically signs and symptoms of liver diseases such as jaundice and ascites, chronic hepatitis (B or C) and renal insufficiency (creatinine clearance < 50 ml/min based on Cockcroft-Gault equation) and history or current use of herbal, supplemental and hepatotoxic non-tuberculosis drugs	100	Hepatotoxicity
Kim, 2009 (GI: KIM)	Korea	Case-control	Assessments performed 2 weeks after onset of treatment and bi-monthly thereafter	All patients with PTB were treated daily with a combination regimen comprising INH (300–400 mg daily), RMP (450–600 mg daily), EMB (600–800 mg daily) and PZA (1000–1500 mg daily) for 2 months and then without PZA for ⩾4 following months. Doses of each drug were adjusted based on body weight of the patient	Newly diagnosed and treated patients with PTB. Exclusion criteria: patients with active or chronic hepatitis, including alcoholic hepatitis, fatty liver disease, liver cirrhosis, carriers of the hepatitis B or C virus, heavy alcohol intake, decreased renal function and severe cardiac diseases requiring several medications	226	ATDH
Kim, 2011 (GI: KIM)	Korea	Case-control	NR	The treatment comprised an initial phase of 2 months and a subsequent continuation phase of ⩾4 months. During the initial phase, 4 drugs were administered, including INH (300–400 mg daily), RMP (450–600 mg daily), EMB (600–800 mg daily) and PZA (1000–1500 mg daily). Doses of each drug were adjusted based on the body weight of the patient. In the following continuation phase, only PZA was discontinued, while the other three drugs were continued	Patients newly diagnosed with PTB and/or TB pleuritis and treated with first-line anti-tuberculosis medications such as INH, RMP, EMB and PZA Exclusion criteria: 1) patients with skin diseases before treatment, 2) chronic renal failure and chronic liver diseases affecting drug metabolism, 3) chronic alcoholism, 4) other chronic medical conditions requiring medication, and 5) non-adherence to treatment	221	ATD-induced MPE
Lee, 2010	Taiwan	Prospective cohort	NR	All patients received oral INH 300 mg, RMP 600 mg (or 450 mg, if body weight was <50 kg), PZA 200 mg/kg of body weight and EMB 800 mg daily for the first 2 months. PZA was then discontinued, while INH, RMP and EMB were continued for another 4 months	Inclusion criteria: adult patients newly diagnosed with active TB, having evident lesions of TB using simple X-ray, computed tomography, sputum smears and cultures positive for mycobacteria Exclusion criteria: 1) positive serum hepatitis B virus surface antigen, antibody to hepatitis C virus; 2) alcoholic liver disease or habitual alcohol drinking; 3) any other hepatic or systemic diseases that may cause liver dysfunction; 4) abnormal serum ALT, AST or bilirubin levels before anti-tuberculosis treatment	140	ATDH
Leiro-Fernandez, 2011	Spain	Case-control	Routine follow-up of clinical assessments every 2 weeks during the first month and monthly thereafter untilcompletion of treatment	Treatment with regimens that included INH, RMP and PZA at the usual dosages (INH 5 mg/kg/day to maximum 300 mg/day, RMP10 mg/kg/day to maximum 600 mg/day and PZA 25–30 mg/kg/day to maximum 2500 mg/day	Inclusion criteria: 1) age 15–75 years; 2) microbiological demonstration of active TB; 3) treatment with regimens that included INH, RMP and PZA at the usual dosages (INH 5 mg/kg/day to max 300 mg/day, RMP 10 mg/kg/day to max 600 mg/day and PZA 25–30 mg/kg/day to max 2500 mg/day); and 4) adequate treatment adherence Exclusion criteria: 1) increased baseline serum transaminases (AST and/or ALT; normal values ⩽ 40 IU/l); 2) positive serological testing for HIV, hepatitis B virus or hepatitis C virus; 3) regular alcohol intake or concomitant use of hepatotoxic drugs; 4) history of chronic liver disease; 5) pregnancy; or 6) no or poor adherence to treatment	117	ATDH
Lv, 2012	China	Case-control	6–9 months	All primary/retreatment patients took INH (600 mg), RMP (600 mg or 450 mg if body weight was <50 kg), PZA (2000 mg) and EMB (1250 mg) every other day in the first 2 months and then INH and RMP were continued for another 4/6 months. The retreatment patients received SM (750 mg) every other day in the first 2 months and continued receiving EMB for another 6 months	Inclusion criteria: sputum smear-positive PTB patients who received standard short-course chemotherapy recommended by the WHO Exclusion criteria: 1) positive serum hepatitis B virus surface antigen or other liver disease, 2) potentially hepatotoxic medications that would confound the picture, 3) abnormal serum ALT, AST or total bilirubin levels before anti-tuberculosis treatment	445	ATDH
Mahmoud, 2012	Tunisia	Prospective cohort	3 months	Treated with INH and RMP containing regimen	Inclusion criteria: adult patients (>18 years) newly diagnosed with TB treated with INH- and RMP-containing regimen, LFTs before initiation of treatment showed completely normal findings on serum ALT, AST, total bilirubin, ALP and gGTP Exclusion criteria: 1) patients receiving other potentially hepatotoxic drugs in addition to anti-tuberculosis agents; 2) positive serum hepatitis B surface antigen or hepatitis C antibody; 3) alcoholic liver disease; 4) any other hepatic or systemic diseases that may cause liver dysfunction	66	INH hepatotoxicity
Ng, 2014	Cases recruited in the UK; controls were samples from elsewhere	Case-control	NR	All had been prescribed INH with all but one also taking additional anti-tuberculosis drugs. Patients were prescribed 300 mg INH/day in line with UK/WHO guidelines	Cases: patients diagnosed with DILI either in the past or at the time of sample collection. Onlycases assessed as having DILI that was highly probably, probably or possibly relating to anti-tuberculosis drug exposure were enrolled in the study. Other possible causes of liver toxicity, particularly hepatitis A, B, C and CMV infection were excluded. No patients included were HIV-positive or had pre-existing liver disease. None had been treated for TB under directly observed treatment Controls: 52 DNA samples from individuals of European ancestry; inclusion/exclusion criteria not reported	127	DILI
Ohno, 2000	Japan	Prospective cohort	3 months	Initial chemotherapy always included INH (400 mg/day; 8.2 ± 2.0 mg/kg/day) and RMP (450 mg/day; 9.2 ± 2.2 mg/kg/day); the third drug used was EMB or SM.	Inclusion criteria: patients had no history of alcohol abuse and were negative for hepatitis B surface antigen and hepatitis C antibody. At the beginning of treatment, LFTs showed completely normal findings on serum AST, ALT, bilirubin, ALP and gGTP Patients receiving other potentially hepatopathic drugs in addition to anti-tuberculosis agents were excluded from the study	77	INH + RMP-induced hepatotoxicity
Possuelo, 2008 (GI: POSSUELO)	Brazil	Prospective cohort	NR	Treatment daily with INH, RMP and PZA for the first 2 months followed by INH and RMP daily for 4 additional months. Drug dosages used were calculated according to patient's weight (weight <45 kg: RMP 300 mg, INH 200 mg, PZA 1000 mg; 45–55 kg: RMP 450 mg, INH 300 mg, PZA 1500 mg; >55 kg: RMP 600 mg, INH 400 mg, PZA 2000 mg)	Inclusion criteria: adult patients (>18 years) newly diagnosed with active TB, who had been treated daily with anti-tuberculosis treatment (see drugs and dosage) Exclusion criteria: patients presenting clinically and laboratory-confirmed liver chronic disease, patients using anti-tuberculosis drugs before enrolment in the study, patients presenting results of LFTs before the beginning of treatment higher that were 2× ULN and refusal to participate of the study	254	ATDH Gastrointestinal ADRs
Rana, 2014 (GI: RANA)	India	Prospective cohort	Patients were monitored every month until the end of treatment or whenever the patients had symptoms or signs of hepatotoxicity	Daily anti-tuberculosis treatment for the first 2 months included INH (300 mg), RMP (600 or 450 mg for body weight/50 kg), PZA (20 mg/kg body weight) and EMB (25 mg/kg body weight). After 2 months, EMB and PZA were discontinued, whereas INH and RMP were continued for an additional 4 months	Inclusion criteria: patients with pulmonary and EPTB Exclusion criteria: patients with history of alcohol abuse and/or any other liver disease; patients who had received other potentially hepatotoxic drugs in addition to anti-tuberculosis drugs; patients who had abnormal serum ALT, AST or bilirubin before starting anti-tuberculosis treatment; patientswho developed viral hepatitis during anti-tuberculosis treatment; patients with renal failure or cancer; and patients who refused blood sampling or providing informed written consent	300	Hepatotoxicity
Santos, 2013 (GI: SANTOS)	Brazil	Prospective cohort	NR	Treatment with INH, RMP and PZA for the first 2 months, followed by INH and RMP daily for 4 months	Inclusion criteria: patients diagnosed with TB and treated with anti-tuberculosis treatment (see drugs and dosage) Exclusion criteria: patients aged <18 years, those with mental disabilities, chronic liver disease confirmed by clinical and laboratory data, users of anti-tuberculosis drugs before enrolment in the study, and those with liver function results > 2× ULN before beginning treatment	270	ATDH
Shimizu, 2006	Japan	Prospective cohort	3 months	Treatment with INH (300–400 mg) and RMP (300–450 mg)	Individuals with PTB without abnormal serum levels of ALT, AST, direct or total bilirubin, or serum hepatitis B virus surface antigen or antibody to hepatitis C virus at the beginning of the study	42	Hepatotoxicity
Singla, 2014	India	Prospective cohort	NR	NR	Inclusion criteria: newly diagnosed patients with TB Exclusion criteria: history of heavy use of alcohol or chronic liver diseases or liver cirrhosis;patients who were settled in the area for a minimum of three generations; people infected with HIV	408	ATDH
Sotsuka, 2011	Japan	Prospective cohort	3 months	INH, RMP and PZA, plus EMB or SM during the first 2 months, followed by administration of INH and RMP plus EMB or SM during the final 4 months	Inclusion criteria: in-patients with active PTB who were treated with the standard Japanese chemotherapy regimen, followed up for >3 months after treatment, and who consented to this study	144	Hepatotoxicity
Teixeira, 2011	Brazil	Case-control	NR	Anti-tuberculosis drug regimens that include INH at the usual dosage (400 mg/day)	Inclusion criteria: 1) age > 18 years, 2) diagnosis of active TB, 3) treatment with anti-tuberculosis drug regimens that include INH at the usual dosage (400 mg/day) and 4) normal baseline serum transaminases (ALT and AST) before treatment Exclusion criteria: 1) positive serological test for the HIV, hepatitis B virus or hepatitis C virus, 2) alcohol abuse, 3) history of chronic liver disease and 4) pregnancy	167	Hepatitis
Vuilleumier, 2006	Switzerland	Prospective cohort	NR	INH 300 mg daily and vitamin B6 40 mg per day for a period of 6 months	Inclusion criteria: patients having LTBI as defined by the American Thoracic Society with normal plasma AST and ALT levels before the beginning of INH monotherapy Exclusion criteria: 1) a history of alcohol consumption, 2) positive serology for the HAV, HBV or HCV, 3) poor chemotherapy compliance (negative urine INH 3 times during the follow-up), loss during the follow-up, 4) patients had to receive supplementary anti-tuberculosis agents or other potentially hepatotoxic drugs	89	INH-induced hepatotoxicity
Wang, 2015 (GI: NTUH)	Taiwan	Prospective cohort	6 months	Daily INH, RMP, EMB and PZA in the first 2 months, and daily INH and RMP for the next 4 months The daily dosage of each drug was calculated by weight	Adult Taiwanese (>16 years) patients with culture-confirmed PTB Subjects were excluded if they were pregnant, had a life expectancy < 6 months, had abnormal baseline LFT or were resistant to INH, RMP or both	355 in the derivation cohort, 182 in the validation cohort	Hepatotoxicity during anti-tuberculosis treatment
Xiang, 2014	China	Prospective cohort	2 months	All patients were prescribed INH (600 mg), RMP (600 mg or 450 mg if the body weight was <50 kg), PZA (2000 mg) and EMB (1250 mg) every other day in the first 2 months. After 2 months, INH and RMP were continued for a further 4–6 months. Retreatment patients in addition received SM (750 mg) every other day in the first 2 months and continued receiving EMB for another 6 months	Inclusion criteria: newly diagnosed PTB patients belonging to the Uyghur ethnic group, who were receiving standard short-course chemotherapy recommended by the WHO, who attended for a 2-month assessment, and any patients attending clinic with suspected liver disease after the start of treatment, before the 2-month visit Exclusion criteria: patients who had signs of abnormal liver function when they started treatment (jaundice or elevated ALT, AST or bilirubin levels) or disease associated with liver dysfunction	2244	ATLI
Yamada, 2009 (GI: YAMADA)	Canada	Prospective cohort	9 months	INH 300 mg	Subjects who were receiving treatment with INH 300 mg daily self-administered for LTBI.Subjects were included if they were aged ⩾19 years; not concurrently receiving other anti-tuberculosis drugs; non-reactive to hepatitis B surface antigen and antibody to hepatitis C virus; absence of any liver or metabolic diseases; without a HIV-positive test result; not consuming ⩾7 alcoholic beverages per day and undergoing sufficiently frequentAST tests to detect hepatotoxicity	170	Hepatotoxicity
Yimer, 2011	Ethiopia	Prospective cohort	followed up for development of DILI for up to 56 weeks	All study participants received RMP based short-course chemotherapy for TB following the national TB treatment guideline. ART was then initiated	Newly diagnosed ART and anti-tuberculosis treatment-naïve adult TB and HIV co-infected patients. The eligibility criteria were age >18 years, CD4 count, 200 cells/Ul, not pregnant and not on other known hepatotoxic drugs concurrently (except cotrimoxazole, 960 mg per day, which was given to all participants before enrolment and during the follow-up period according to the treatment guideline)	353	Anti-tubercular and antiretroviral DILI
Yuliwulandari, 2016	Indonesia	Case-control	NR	NR (anti-tuberculosis treatment)	Patients included in the study were male and female aged 15–70 years, suffering from TB according to the WHO standards, and were under monitored treatment with anti-tuberculosis drugs Exclusion criteria: 1) history of liver disease such as: hepatitis A B or C; hepatoma; liver cirrhosis or cholelithiasis positive; 2) abnormal levels of any LFT (ALT, AST or total bilirubin) before anti-tuberculosis treatment	241	ATLI
Zaverucha-do-Valle, 2014	Brazil	Retrospective cohort	Follow-up at days 15 and 30 and at least one visit monthly until the end of treatment	600 mg/day of RMP, 400 mg/day of INH and 2 g/day of PZA for all patients with corporal weight >45 kg or adjusted for corporal weight <45 kg. After 2 months of treatment, PZA was discontinued	Inclusion criteria: signed written consent, sputum smear with AFB or culture positive for M. tuberculosis; ongoing TB treatment and laboratory tests of liver function Exclusion criteria: age <18 years, pregnancy and no >1 visit registered	131	ATDH

INH =isoniazid; RMP =rifampicin; PZA =pyrazinamide; EMB =ethambutol; ALT =alanine aminotransferase; AST =aspartate aminotransferase; RCT =randomised controlled trial; HIV =human immunodeficiency virus; SM =streptomycin; PTB =pulmonary tuberculosis; IU =international unit; ALP =alkaline phosphatase; DILI =drug-induced liver injury; LFT =liver function test; DIH =drug-induced hepatotoxicity; NR =not reported; ATDH =anti-tuberculosis drug-induced hepatotoxicity; GI =group identifier; ADR =adverse drug reaction; ULN =upper limit of normal; EPTB =extra-pulmonary TB; ADIH =anti-tuberculosis drug-induced hepatitis; ATD =anti-tuberculosis drug; MPE =maculopapular eruption; WHO = World Health Organization; gGTP = gamma-glutamyltranspeptidase; CMV = cytomegalovirus ; LTBI = latent tuberculous infection; ART =antiretroviral therapy; ATLI = anti-tuberculosis drug-induced liver injury.

**Table A.4:** Definitions of hepatotoxicity in the included studies

Author, year	Outcome and definition
An, 2012	ATDH was defined as an increase of >2× ULN range in ALT or conjugated bilirubin levels or a concurrent increase in AST levels, according to the criteria of DILI developed at an international consensus meeting^1^
Azuma, 2013	INH-DILI was assessed according to the diagnostic criteria of the Manual for Serious Side Effects of Drug-induced Liver Injury from the Ministry of Health, Labor and Welfare of Japan.^2^,^3^ In brief, hepatocellular injury was defined as a >2-fold increase in the ULN concentration of ALT alone or a serum ALT ratio/ALP ratio > 5, where the ALT ratio = ALT value/ULN of ALT, and ALP ratio = ALP value/ULN of ALP. Cholestatic injury was defined as an increase above 2-fold of the ULN range of ALP or a serum ALT ratio/ALP ratio < 2. Mixed injury was defined as a serum ALT ratio/ALP ratio of between 2 and 5. Causality assessments showed a relationship to the INH administration if the total score was more than grade 3, i.e. ‘possible’
Bose, 2011; Yimer 2011	ATDH (Bose 2011)/DILI (Yimer 2011) in patients was defined according to the international consensus criteria.^1^ Liver biochemical parameters >2 times the ULN value was considered as hepatotoxicity
Çetintaş 3 2008	Drug-induced hepatitis criteria were defined as follows: 1) an increase in AST and ALT levels of >3-fold above normal or >5-fold above starting level or, 2) a greater than normal increase in ALT and AST levels together with hepatitis symptoms or, 3) a high bilirubin level
Chamorro, 2013	Hepatotoxicity was defined as when serum transaminase concentrations were at least 3× ULN (normal values: AST 0–32 IU/l and ALT 0–31 IU/l) with report of jaundice (bilirubin normal values: 0–1 mg/dl) and/or hepatitis symptoms (nausea, vomiting, abdominal pain), or >5× ULN with or without symptoms
Chang, 2012	ATDH was ‘defined according to the classification of the CIOMS’.^1^ No further information was provided
Cho, 2007; Jung, 2015; Lee, 2010	ATDH was designated as an increase in serum ALT level > 2× ULN after anti-tuberculosis treatment, according to the criteria for DILI developed by the international consensus meeting^1^
Feng, 2014; Teixeira, 2011	Anti-tuberculosis drug-induced hepatitis (Teixeira 2011)/anti-tuberculosis drug-induced hepatic injury (Feng 2014): an increase in serum transaminase values to >3× ULN values (40 IU/l ALT in Feng) and symptoms compatible with hepatitis
Fredj, 2016	The causality of drug-induced hepatotoxicity was determined according to the report of an international consensus meeting.^1^ These criteria include 1) an increase of liver transaminases levels of >2 times above the normal value (<40 IU/l) for AST and ALT, 2) an improvement of this pattern after the drug withdrawal, and 3) the absence of alternative causes of this disorder
Gupta, 2013 (GI: GUPTA)	Increase in ALT > 2× ULN or a combined increase in AST and bilirubin levels, provided one of them is >2× ULN, was defined as ATDH according to the international consensus meeting^1^
Higuchi, 2007	DIH was defined according to the criteria of the international consensus meeting,^1^ i.e., development of a ⩾2-fold increase in serum ALT level above the ULN range: N (642 IU/l), or a combined increase of > 2 N in serum AST (N ⩽ 33 IU/l) and total bilirubin (N ⩽ 1.5 mg/dl)
Ho, 2013	The criteria for the diagnosis of hepatotoxicity was an elevation in liver function tests, AST and/or ALT of >5 × ULN; or AST and/or ALT of >3× ULN in the presence of symptoms such as nausea, vomiting, poor appetite, abdominal pain or jaundice; or AST and/or ALT of >3× ULN in the presence of total bilirubin of >2× ULN
Huang, 2003 (GI: HUANG)	Anti-tuberculosis drug-induced hepatitis was diagnosed as 1) an increase in serum ALT level > 2× ULN during treatment, according to the criteria established by the international consensus meeting;^1^ 2) negative serum HBV surface antigen, IgM antibody to HAV, and antibody to HCV when ALT or AST is elevated; 3) without any other major hepatic or systemic diseases that may induce elevation of liver biochemical tests, such as alcoholic liver disease, autoimmune hepatitis, congestive heart failure, hypoxia, and bacteremia; and 4) a causality assessment score > 5 (when classified as ‘probable’ or ‘highly probable’ drug-induced hepatitis), as derived from the international consensus meeting^1^
Khalili, 2011	Hepatotoxicity was defined as 1) increased levels of liver transaminases > 3 times above the normal value (<40 U/l for AST and ALT) with any other clinical signs and symptoms; or 2) elevation of transaminases > 5× ULN, if patients had no symptoms. For evaluation of causality, The Roussel Uclaf Causality Assessment Method scoring system was used^2^
Kim, 2009 (GI: KIM)	Anti-tuberculosis drug-induced hepatitis was defined as an elevation in the serum levels of ALT > 2× ULN (⩽40 U/ml) during treatment and normalisation of these values after cessation of medication according to the criteria from the international consensus meeting^1^
Leiro-Fernandez, 2011	ATDH was defined as an increase in serum transaminase (either AST or ALT) to values > 3× ULN (i.e., >120 IU/l) at any time during the treatment period
Lv, 2012	ATDH was designated as an increase of >2× ULN value in ALT or a combined increase in AST and total bilirubin provided one of them is >2× ULN. In this study, the ULN of ALT, AST and total bilirubin were respectively 40 U/l, 40 U/l and 19 μmol/l Causality assessment result was highly probable, probable or possible based on the CIOMS scale^1^
Mahmoud, 2012	DIH was diagnosed as 1) an increase in serum ALT level greater than twice the ULN during the treatment, according to the criteria established by the international consensus meeting;^1^ 2) negative serum HBV surface antigen, IgM antibody to HAV, and antibody to HCV when ALT or AST was elevated; 3) without any other major hepatic or systemic diseases that may induce elevation of liver biochemical tests, such as alcoholic liver disease, autoimmune hepatitis, congestive heart failure, hypoxia, and bacteremia; when the French imputability score^4^ was classified as ‘probable’ or ‘likely’ or ‘certain’
Ng, 2014	All cases of DILI met at least one of the following biochemical criteria for enrolment into this study: 1) ALT > 5× ULN, 2) ALP > 2× ULN, or 3) ALT > 3× ULN and bilirubin > 2× ULN
Ohno, 2000	Hepatotoxicity was estimated as follows: AST and/or ALT > 1.5× ULN and 2× before administration
Possuelo, 2008 (GI: POSSUELO)	Criteria for the diagnosis of hepatotoxicity was an elevation in liver function tests, AST and/or ALT of >3× ULN (reference: respectively 40 and 65 U/l) and/or in total bilirubin up to >2.0 mg/dl in the presence of such gastrointestinal symptoms as anorexia, nausea, vomiting and/or jaundice, with a normalisation of serum ALT level after discontinuation of the anti-tuberculosis drugs
Rana, 2014 (GI: RANA)	ATDH was defined according to international consensus criteria.^1^ Patients with a rise in serum AST or ALT levels ⩾ 5× ULN, irrespective of symptoms and serum bilirubin levels, or patients with rise in serum AST or ALT levels ⩾ 2× ULN with hyperbilirubinaemia and an absence of serological evidence of infection with hepatitis viruses (A, B, C and E) were considered as having ATDH
Santos, 2013 (GI: SANTOS)	Hepatotoxicity was defined as an increase in serum ALT level in excess of three times the ULN after INH treatment
Shimizu, 2006	Hepatotoxicity was defined as an ALT and/or AST level more than twice the institutional ULN according to the modified criteria of the international consensus meeting for drug-induced liver disorders.^1^ The ULN for AST was 33 IU/l and that for ALT was 42 IU/l
Singla, 2014	International consensus criteria^1^ define ATDH as development of >2× ULN value of ALT and AST. The ULN values used in this study were 35 U/l ALT and 40 U/l AST
Sotsuka, 2011	The severity of hepatotoxicity (hepatotoxicity A–D) was judged by the increase in either AST or ALT levels from the ULN range (AST, 33 U/l; ALT, 42 U/l): hepatotoxicity A, above the upper limit and less than 2-fold increase; hepatotoxicity B, 2- to 3-fold increase; hepatotoxicity C, 3- to 4-fold increase; hepatotoxicity D, greater than 4-fold increase. Results for grades B–D of hepatotoxicity were used in this review as clinical opinion was that the hepatotoxicity A patients would not have met the criteria for hepatotoxicity in many of the other studies included in this review
Vuilleumier, 2006	Criteria for the diagnosis of INH-H comprised elevation in AST and/or ALT levels 4-fold above the upper reference limit (168 UI/l) with or without symptoms. CDS were used to assess the likelihood of drug involvement when INH-H was suspected.^5^ Based on the CDS, causality assessment of INH-H was then categorised as definite (score > 17), probable (14–17), possible (10–13), unlikely (6–9) or excluded (<6). INH-H with possible to probable scores were considered for statistical analysis; unlikely scores were still considered when no other factor was identifiable
Wang, 2011 (GI: NTUH)	HATT was defined as increased serum AST and/or ALT > 1.5 times the baseline level. Results are presented for drug-induced HATT and virus-induced HATT separately. In this review, we used the results for drug-induced HATT. The diagnosis of INH- or RMP-induced HATT required a positive re-challenge test (at least doubling of serum AST or ALT level and recurrence of clinical symptoms of hepatitis after re-challenge), whereas PZA-induced HATT was diagnosed by exclusion
Wang, 2015 (GI: NTUH)	Hepatitis during anti-tuberculosis treatment was defined as increased serum AST and/or ALT > 3× ULN in symptomatic patients, or >5× ULN in asymptomatic patients. The diagnosis of INH- or RMP-induced hepatitis required a positive re-challenge test (at least doubling of serum AST or ALT levels and recurrence of clinical symptoms of hepatitis after re-challenge), whereas PZA-induced hepatitis was diagnosed either by a positive re-challenge test or by exclusion. Results are presented for overall drug-induced HATT and INH-induced HATT separately. In this review, we used the results for overall drug-induced HATT as our review focuses on hepatotoxicity induced by any anti-tuberculosis drug
Xiang, 2014	ATLI was defined as an ALT, AST or bilirubin value > 2× ULN. The ULN used in the study was 40 Ul for ALT, 40 U/l for AST, and 19 mmol/l for total bilirubin
Yamada, 2009	ATDH was defined as an increase in serum AST level > 2× ULN during 9 months of treatment with INH according to the criteria of the international consensus meeting in Paris;^1^ normalisation of serum AST level after discontinuation of INH; and a causality assessment score^2^ of >8, corresponding to the category of highly probable hepatotoxicity
Yuliwulandari, 2016	ATLI: definition not reported
Zaverucha-do-Valle, 2014	Hepatotoxicity was defined as 2× ULN (ALT 42 IU/l) or at least a 2-fold increase in ALT initial levels in patients with a baseline ALT of >84 IU/l during the treatment period

ULN = upper limit of normal; ALT =alanine aminotransferase; AST =aspartate aminotransferase; DILI = drug-induced liver injury; INH = isoniazid; ALP = alkaline phosphatase; ATDH=anti-tuberculosis drug-induced hepatotoxicity; IU=international unit; DIH=drug-induced hepatotoxicity; CIOMS=Council for International Organizations of Medical Sciences; GI=group identifier; HBV =hepatitis B virus; IgM =immunoglobulin M; HAV=hepatitis A virus; HCV =hepatitis C virus; CDS = Clinical Diagnostic Scale; INH-H =INH-induced hepatitis; RMP =rifampicin; PZA =pyrazinamide; HATT =hepatitis during anti-tuberculosis treatment; ATLI =anti-tuberculosis drug-induced liver injury;

**Table A.4:** (*continued*)

Author, year	Outcome and definition
An, 2012	ATDH was defined as an increase of >2× ULN range in ALT or conjugated bilirubin levels or a concurrent increase in AST levels, according to the criteria of DILI developed at an international consensus meeting^1^
Azuma, 2013	INH-DILI was assessed according to the diagnostic criteria of the Manual for Serious Side Effects of Drug-induced Liver Injury from the Ministry of Health, Labor and Welfare of Japan.^2^,^3^ In brief, hepatocellular injury was defined as a >2-fold increase in the ULN concentration of ALT alone or a serum ALT ratio/ALP ratio > 5, where the ALT ratio = ALT value/ULN of ALT, and ALP ratio = ALP value/ULN of ALP. Cholestatic injury was defined as an increase above 2-fold of the ULN range of ALP or a serum ALT ratio/ALP ratio < 2. Mixed injury was defined as a serum ALT ratio/ALP ratio of between 2 and 5. Causality assessments showed a relationship to the INH administration if the total score was more than grade 3, i.e. ‘possible’
Bose, 2011; Yimer 2011	ATDH (Bose 2011)/DILI (Yimer 2011) in patients was defined according to the international consensus criteria.^1^ Liver biochemical parameters >2 times the ULN value was considered as hepatotoxicity
Çetintaş 3 2008	Drug-induced hepatitis criteria were defined as follows: 1) an increase in AST and ALT levels of >3-fold above normal or >5-fold above starting level or, 2) a greater than normal increase in ALT and AST levels together with hepatitis symptoms or, 3) a high bilirubin level
Chamorro, 2013	Hepatotoxicity was defined as when serum transaminase concentrations were at least 3× ULN (normal values: AST 0–32 IU/l and ALT 0–31 IU/l) with report of jaundice (bilirubin normal values: 0–1 mg/dl) and/or hepatitis symptoms (nausea, vomiting, abdominal pain), or >5× ULN with or without symptoms
Chang, 2012	ATDH was ‘defined according to the classification of the CIOMS’.^1^ No further information was provided
Cho, 2007; Jung, 2015; Lee, 2010	ATDH was designated as an increase in serum ALT level > 2× ULN after anti-tuberculosis treatment, according to the criteria for DILI developed by the international consensus meeting^1^
Feng, 2014; Teixeira, 2011	Anti-tuberculosis drug-induced hepatitis (Teixeira 2011)/anti-tuberculosis drug-induced hepatic injury (Feng 2014): an increase in serum transaminase values to >3× ULN values (40 IU/l ALT in Feng) and symptoms compatible with hepatitis
Fredj, 2016	The causality of drug-induced hepatotoxicity was determined according to the report of an international consensus meeting.^1^ These criteria include 1) an increase of liver transaminases levels of >2 times above the normal value (<40 IU/l) for AST and ALT, 2) an improvement of this pattern after the drug withdrawal, and 3) the absence of alternative causes of this disorder
Gupta, 2013 (GI: GUPTA)	Increase in ALT > 2× ULN or a combined increase in AST and bilirubin levels, provided one of them is >2× ULN, was defined as ATDH according to the international consensus meeting^1^
Higuchi, 2007	DIH was defined according to the criteria of the international consensus meeting,^1^ i.e., development of a ⩾2-fold increase in serum ALT level above the ULN range: N (642 IU/l), or a combined increase of > 2 N in serum AST (N ⩽ 33 IU/l) and total bilirubin (N ⩽ 1.5 mg/dl)
Ho, 2013	The criteria for the diagnosis of hepatotoxicity was an elevation in liver function tests, AST and/or ALT of >5 × ULN; or AST and/or ALT of >3× ULN in the presence of symptoms such as nausea, vomiting, poor appetite, abdominal pain or jaundice; or AST and/or ALT of >3× ULN in the presence of total bilirubin of >2× ULN
Huang, 2003 (GI: HUANG)	Anti-tuberculosis drug-induced hepatitis was diagnosed as 1) an increase in serum ALT level > 2× ULN during treatment, according to the criteria established by the international consensus meeting;^1^ 2) negative serum HBV surface antigen, IgM antibody to HAV, and antibody to HCV when ALT or AST is elevated; 3) without any other major hepatic or systemic diseases that may induce elevation of liver biochemical tests, such as alcoholic liver disease, autoimmune hepatitis, congestive heart failure, hypoxia, and bacteremia; and 4) a causality assessment score > 5 (when classified as ‘probable’ or ‘highly probable’ drug-induced hepatitis), as derived from the international consensus meeting^1^
Khalili, 2011	Hepatotoxicity was defined as 1) increased levels of liver transaminases > 3 times above the normal value (<40 U/l for AST and ALT) with any other clinical signs and symptoms; or 2) elevation of transaminases > 5× ULN, if patients had no symptoms. For evaluation of causality, The Roussel Uclaf Causality Assessment Method scoring system was used^2^
Kim, 2009 (GI: KIM)	Anti-tuberculosis drug-induced hepatitis was defined as an elevation in the serum levels of ALT > 2× ULN (⩽40 U/ml) during treatment and normalisation of these values after cessation of medication according to the criteria from the international consensus meeting^1^
Leiro-Fernandez, 2011	ATDH was defined as an increase in serum transaminase (either AST or ALT) to values > 3× ULN (i.e., >120 IU/l) at any time during the treatment period
Lv, 2012	ATDH was designated as an increase of >2× ULN value in ALT or a combined increase in AST and total bilirubin provided one of them is >2× ULN. In this study, the ULN of ALT, AST and total bilirubin were respectively 40 U/l, 40 U/l and 19 μmol/l Causality assessment result was highly probable, probable or possible based on the CIOMS scale^1^
Mahmoud, 2012	DIH was diagnosed as 1) an increase in serum ALT level greater than twice the ULN during the treatment, according to the criteria established by the international consensus meeting;^1^ 2) negative serum HBV surface antigen, IgM antibody to HAV, and antibody to HCV when ALT or AST was elevated; 3) without any other major hepatic or systemic diseases that may induce elevation of liver biochemical tests, such as alcoholic liver disease, autoimmune hepatitis, congestive heart failure, hypoxia, and bacteremia; when the French imputability score^4^ was classified as ‘probable’ or ‘likely’ or ‘certain’
Ng, 2014	All cases of DILI met at least one of the following biochemical criteria for enrolment into this study: 1) ALT > 5× ULN, 2) ALP > 2× ULN, or 3) ALT > 3× ULN and bilirubin > 2× ULN
Ohno, 2000	Hepatotoxicity was estimated as follows: AST and/or ALT > 1.5× ULN and 2× before administration
Possuelo, 2008 (GI: POSSUELO)	Criteria for the diagnosis of hepatotoxicity was an elevation in liver function tests, AST and/or ALT of >3× ULN (reference: respectively 40 and 65 U/l) and/or in total bilirubin up to >2.0 mg/dl in the presence of such gastrointestinal symptoms as anorexia, nausea, vomiting and/or jaundice, with a normalisation of serum ALT level after discontinuation of the anti-tuberculosis drugs
Rana, 2014 (GI: RANA)	ATDH was defined according to international consensus criteria.^1^ Patients with a rise in serum AST or ALT levels ⩾ 5× ULN, irrespective of symptoms and serum bilirubin levels, or patients with rise in serum AST or ALT levels ⩾ 2× ULN with hyperbilirubinaemia and an absence of serological evidence of infection with hepatitis viruses (A, B, C and E) were considered as having ATDH
Santos, 2013 (GI: SANTOS)	Hepatotoxicity was defined as an increase in serum ALT level in excess of three times the ULN after INH treatment
Shimizu, 2006	Hepatotoxicity was defined as an ALT and/or AST level more than twice the institutional ULN according to the modified criteria of the international consensus meeting for drug-induced liver disorders.^1^ The ULN for AST was 33 IU/l and that for ALT was 42 IU/l
Singla, 2014	International consensus criteria^1^ define ATDH as development of >2× ULN value of ALT and AST. The ULN values used in this study were 35 U/l ALT and 40 U/l AST
Sotsuka, 2011	The severity of hepatotoxicity (hepatotoxicity A–D) was judged by the increase in either AST or ALT levels from the ULN range (AST, 33 U/l; ALT, 42 U/l): hepatotoxicity A, above the upper limit and less than 2-fold increase; hepatotoxicity B, 2- to 3-fold increase; hepatotoxicity C, 3- to 4-fold increase; hepatotoxicity D, greater than 4-fold increase. Results for grades B–D of hepatotoxicity were used in this review as clinical opinion was that the hepatotoxicity A patients would not have met the criteria for hepatotoxicity in many of the other studies included in this review
Vuilleumier, 2006	Criteria for the diagnosis of INH-H comprised elevation in AST and/or ALT levels 4-fold above the upper reference limit (168 UI/l) with or without symptoms. CDS were used to assess the likelihood of drug involvement when INH-H was suspected.^5^ Based on the CDS, causality assessment of INH-H was then categorised as definite (score > 17), probable (14–17), possible (10–13), unlikely (6–9) or excluded (<6). INH-H with possible to probable scores were considered for statistical analysis; unlikely scores were still considered when no other factor was identifiable
Wang, 2011 (GI: NTUH)	HATT was defined as increased serum AST and/or ALT > 1.5 times the baseline level. Results are presented for drug-induced HATT and virus-induced HATT separately. In this review, we used the results for drug-induced HATT. The diagnosis of INH- or RMP-induced HATT required a positive re-challenge test (at least doubling of serum AST or ALT level and recurrence of clinical symptoms of hepatitis after re-challenge), whereas PZA-induced HATT was diagnosed by exclusion
Wang, 2015 (GI: NTUH)	Hepatitis during anti-tuberculosis treatment was defined as increased serum AST and/or ALT > 3× ULN in symptomatic patients, or >5× ULN in asymptomatic patients. The diagnosis of INH- or RMP-induced hepatitis required a positive re-challenge test (at least doubling of serum AST or ALT levels and recurrence of clinical symptoms of hepatitis after re-challenge), whereas PZA-induced hepatitis was diagnosed either by a positive re-challenge test or by exclusion. Results are presented for overall drug-induced HATT and INH-induced HATT separately. In this review, we used the results for overall drug-induced HATT as our review focuses on hepatotoxicity induced by any anti-tuberculosis drug
Xiang, 2014	ATLI was defined as an ALT, AST or bilirubin value > 2× ULN. The ULN used in the study was 40 Ul for ALT, 40 U/l for AST, and 19 mmol/l for total bilirubin
Yamada, 2009	ATDH was defined as an increase in serum AST level > 2× ULN during 9 months of treatment with INH according to the criteria of the international consensus meeting in Paris;^1^ normalisation of serum AST level after discontinuation of INH; and a causality assessment score^2^ of >8, corresponding to the category of highly probable hepatotoxicity
Yuliwulandari, 2016	ATLI: definition not reported
Zaverucha-do-Valle, 2014	Hepatotoxicity was defined as 2× ULN (ALT 42 IU/l) or at least a 2-fold increase in ALT initial levels in patients with a baseline ALT of >84 IU/l during the treatment period

ULN = upper limit of normal; ALT =alanine aminotransferase; AST =aspartate aminotransferase; DILI = drug-induced liver injury; INH = isoniazid; ALP = alkaline phosphatase; ATDH=anti-tuberculosis drug-induced hepatotoxicity; IU=international unit; DIH=drug-induced hepatotoxicity; CIOMS=Council for International Organizations of Medical Sciences; GI=group identifier; HBV =hepatitis B virus; IgM =immunoglobulin M; HAV=hepatitis A virus; HCV =hepatitis C virus; CDS = Clinical Diagnostic Scale; INH-H =INH-induced hepatitis; RMP =rifampicin; PZA =pyrazinamide; HATT =hepatitis during anti-tuberculosis treatment; ATLI =anti-tuberculosis drug-induced liver injury;

**Table A.5:** Definitions of other toxicity outcomes in the included studies

Outcome	Author, year	Outcome definition
Peripheral neuropathy	Azuma, 2013	NR
	Dhoro, 2013	NR
Adverse DIH outcome	Bose, 2011	‘16 [patients] showed an adverse outcome of anti-tuberculosis treatment hepatotoxicity with icterus, severe nausea and vomiting’. No further details reported
ADRs	Costa, 2012	The presence of at least one of the following symptoms during the follow-up period: gastric, joint, neuromuscular or skin reactions; and hepatotoxicity (in accordance with the criteria of drug-induced liver injuries developed by the international consensus meeting)^6^
Skin rash	Higuchi, 2007	NR
Eosinophilia	Higuchi, 2007	The presence of >450 eosinophils/ml
ATD-induced MPE	Kim, 2011 (GI: KIM)	The development of MPE after receiving first-line ATD and the disappearance of MPE after discontinuing ATD due to MPE
Gastrointestinal ADRs	Possuelo, 2008 (GI: POSSUELO)	Anorexia, nausea, vomiting and/or abdominal pain

NR = not reported; DIH = drug-induced hepatotoxicity; ADR = adverse drug reaction; ATD = anti-tuberculosis drug; MPE = macropapular eruption; GI = group identifier.

## References

[1] World Health Organization (2017). Global tuberculosis report, 2017.

[2] Tostmann A, Boeree M J, Aarnoutse R E, De Lange W C M, Van Der Ven A J A M, Dekhuijzen R (2008). Antituberculosis drug-induced hepatotoxicity: concise up-to-date review. J Gastroenterol Hepatol.

[3] Dash L A, Comstock G W, Flynn J P (1980). Isoniazid preventive therapy: retrospect and prospect. Am Rev Respir Dis.

[4] Aarnoutse R, Donald P R, Helden P D (2011). Pharmacogenetics of anti-tuberculosis drugs. Antituberculosis chemotherapy.

[5] Roy P D, Majumder M, Roy B (2008). Pharmacogenomics of anti-TB drugs-related hepatotoxicity. Pharmacogenomics.

[6] Sun F, Chen Y, Xiang Y, Zhan S (2008). Drug-metabolising enzyme polymorphisms and predisposition to anti-tuberculosis drug-induced liver injury: a meta-analysis. Int J Tuberc Lung Dis.

[7] Lauterburg B, Smith C, Todd E, Mitchell J (1985). Pharmacokinetics of the toxic hydrazino metabolites formed from isoniazid in humans. J Pharmacol Exp Ther.

[8] Pandit A, Sachdeva T, Bafna P (2012). Drug-induced hepatotoxicity: a review. J Appl Pharm Sci.

[9] Kaplowitz N, DeLeve L D (2013). Drug-induced liver disease.

[10] Li L M, Chen L, Deng G H (2012). SLCO1B1 *15 haplotype is associated with rifampin-induced liver injury. Mol Med Rep.

[11] Ramachandran G, Swaminathan S (2012). Role of pharmacogenomics in the treatment of tuberculosis: a review. Pharmacogenomics Pers Med.

[12] Cai Y, Yi J, Zhou C, Shen X (2012). Pharmacogenetic study of drug-metabolising enzyme polymorphisms on the risk of anti-tuberculosis drug-induced liver injury: a meta-analysis. PLOS ONE.

[13] Du H, Chen X, Fang Y (2013). Slow N-acetyltransferase 2 genotype contributes to anti-tuberculosis drug-induced hepatotoxicity: a meta-analysis. Mol Biol Rep.

[14] Shi J, Xie M, Wang J, Xu Y, Liu X (2015). Susceptibility of *N*acetyltransferase 2 slow acetylators to antituberculosis drug-induced liver injury: a meta-analysis. Pharmacogenomics.

[15] Wang P, Xie S, Hao Q, Zhang C, Jiang B (2012). *NAT2* polymorphisms and susceptibility to anti-tuberculosis drug-induced liver injury: a meta-analysis. Int J Tuberc Lung Dis.

[16] Richardson M, Kirkham J, Dwan K, Sloan D, Davies G, Jorgensen A (2017). Influence of genetic variants on toxicity to anti-tubercular agents: a systematic review and meta-analysis (protocol). Syst Rev.

[17] Veritas Health Innovation (2018). Covidence systematic review software.

[18] Higgins J, Green S (2011). Cochrane handbook for systematic reviews of interventions, version 5.1.0 [updated March 2011].

[19] Little J, Higgins J (2006). The HuGENet^™^ HuGE Review Handbook, version 1.0.

[20] Jorgensen A L, Williamson P R (2008). Methodological quality of pharmacogenetic studies: issues of concern. Stat Med.

[21] StataCorp (2015). Stata Statistical Software: Release 14.

[22] DerSimonian R, Laird N (1986). Meta-analysis in clinical trials. Control Clin Trials.

[23] Mantel N, Haenszel W (1959). Statistical aspects of the analysis of data from retrospective studies of disease. J Natl Cancer Inst.

[24] Moher D, Liberati A, Tetzlaff J, Altman D G, The PRISMA Group (2009). Preferred Reporting Items for Systematic Reviews and Meta-Analyses: the PRISMA statement. PLOS Med.

[25] An H R, Wu X Q, Wang Z Y, Zhang J X, Liang Y (2012). NAT2 and CYP2E1 polymorphisms associated with antituberculosis drug-induced hepatotoxicity in Chinese patients. Clin Exp Pharmacol Physiol.

[26] Azuma J, Ohno M, Kubota R (2013). NAT2 genotype guided regimen reduces isoniazid-induced liver injury and early treatment failure in the 6-month four-drug standard treatment of tuberculosis: a randomized controlled trial for pharmacogenetics-based therapy. Eur J Clin Pharmacol.

[27] Bose P D, Sarma M P, Medhi S, Das B C, Husain S A, Kar P (2011). Role of polymorphic *N*acetyl transferase 2 and cytochrome P4502E1 gene in antituberculosis treatment-induced hepatitis. J Gastroenterol Hepatol.

[28] Çetintaş V B, Erer O F, Kosova B (2008). Determining the relation between *N*acetyltransferase-2 acetylator phenotype and antituberculosis drug induced hepatitis by molecular biologic tests. Tuberk Toraks.

[29] Chamorro J G, Castagnino J P, Musella R M (2013). Sex, ethnicity, and slow acetylator profile are the major causes of hepatotoxicity induced by antituberculosis drugs. J Gastroenterol Hepatol.

[30] Chang J, Liu E, Lee C (2012). UGT1A1 polymorphisms associated with risk of induced liver disorders by anti-tuberculosis medications. Int J Tuberc Lung Dis.

[31] Cho H-J, Koh W-J, Ryu Y-J (2007). Genetic polymorphisms of NAT2 and CYP2E1 associated with antituberculosis drug-induced hepatotoxicity in Korean patients with pulmonary tuberculosis. Tuberculosis.

[32] Costa G N, Magno L A, Santana C V (2012). Genetic interaction between NAT2, GSTM1, GSTT1, CYP2E1, and environmental factors is associated with adverse reactions to anti-tuberculosis drugs. Mol Diagn Ther.

[33] Dhoro M, Ngara B, Kadzirange G, Nhachi C, Masimirembwa C (2013). Genetic variants of drug metabolizing enzymes and drug transporter (ABCB1) as possible biomarkers for adverse drug reactions in an HIV/AIDS cohort in Zimbabwe. Current HIV Res.

[34] Feng F, Guo M, Chen Y (2014). Genetic polymorphisms in metabolic enzymes and susceptibility to anti-tuberculosis drug-induced hepatic injury. Genet Mol Res.

[35] Fredj N B, Gam R, Kerkni E (2017). Risk factors of isoniazid-induced hepatotoxicity in Tunisian tuberculosis patients. Pharmacogenomics J.

[36] Gupta V H, Amarapurkar D N, Singh M (2013). Association of N-acetyltransferase 2 and cytochrome P450 2E1 gene polymorphisms with antituberculosis drug-induced hepatotoxicity in Western India. J Gastroenterol Hepatol.

[37] Higuchi N, Tahara N, Yanagihara K (2007). NAT2* 6A, a haplotype of the *N*acetyltransferase 2 gene, is an important biomarker for risk of anti-tuberculosis drug-induced hepatotoxicity in Japanese patients with tuberculosis. World J Gastroenterol.

[38] Ho H-T, Wang T-H, Hsiong C-H (2013). The NAT2 tag SNP *rs*1495741 correlates with the susceptibility of antituberculosis drug-induced hepatotoxicity. Pharmacogenet Genom.

[39] Huang Y S, Chern H D, Su W J (2003). Cytochrome P450 2E1 genotype and the susceptibility to anti-tuberculosis drug-induced hepatitis. Hepatology.

[40] Jung J A, Kim T-E, Lee H (2015). A proposal for an individualized pharmacogenetic-guided isoniazid dosage regimen for patients with tuberculosis. Drug Des Devel Ther.

[41] Khalili H, Fouladdel S, Sistanizad M, Hajiabdolbaghi M, Azizi E (2011). Association of *N*acetyltransferase-2 genotypes and anti-tuberculosis induced liver injury: first case-controlled study from Iran. Curr Drug Saf.

[42] Kim S-H, Kim S-H, Bahn J-W (2009). Genetic polymorphisms of drug-metabolizing enzymes and anti-TB drug-induced hepatitis. Pharmacogenomics.

[43] Kim S-H, Kim S-H, Yoon H J (2011). NAT2, CYP2C9, CYP2C19, and CYP2E1 genetic polymorphisms in anti-TB drug-induced maculopapular eruption. Eur J Clin Pharmacol.

[44] Lee S, Chung L, Huang H, Chuang T, Liou Y, Wu L (2010). *NAT2* and CYP2E1 polymorphisms and susceptibility to first-line anti-tuberculosis drug-induced hepatitis. Int J Tuberc Lung Dis.

[45] Leiro-Fernandez V, Valverde D, Vázquez-Gallardo R (2011). N-acetyltransferase 2 polymorphisms and risk of anti-tuberculosis drug-induced hepatotoxicity in Caucasians. Int J Tuberc Lung Dis.

[46] Lv X, Tang S, Xia Y (2012). NAT2 genetic polymorphisms and anti-tuberculosis drug-induced hepatotoxicity in Chinese community population. Ann Hepatol.

[47] Mahmoud L B, Ghozzi H, Kamoun A (2012). Polymorphism of the *N*acetyltransferase 2 gene as a susceptibility risk factor for antituberculosis drug-induced hepatotoxicity in Tunisian patients with tuberculosis. Pathol Biol (Paris).

[48] Singla N, Gupta D, Birbian N, Singh J (2014). Association of NAT2, GST and CYP2E1 polymorphisms and anti-tuberculosis drug-induced hepatotoxicity. Tuberculosis.

[49] Ohno M, Yamaguchi I, Yamamoto I (2000). Slow *N*acetyltransferase 2 genotype affects the incidence of isoniazid and rifampicin-induced hepatotoxicity.

[50] Possuelo L, Castelan J, De Brito T (2008). Association of slow *N*acetyltransferase 2 profile and anti-TB drug-induced hepatotoxicity in patients from Southern Brazil. Eur J Clin Pharmacol.

[51] Rana S, Sharma S, Ola R (2014). N-acetyltransferase 2, cytochrome P4502E1 and glutathione S-transferase genotypes in antitubercular treatment-induced hepatotoxicity in North Indians. J Clin Pharm Ther.

[52] Ng C S, Hasnat A, Al Maruf A (2014). N-acetyltransferase 2 (NAT2) genotype as a risk factor for development of drug-induced liver injury relating to antituberculosis drug treatment in a mixed-ethnicity patient group. Eur J Clin Pharmacol.

[53] Santos N, Callegari-Jacques S, Ribeiro dos Santos A (2013). *N*acetyl transferase 2 and cytochrome P450 2E1 genes and isoniazid-induced hepatotoxicity in Brazilian patients. Int J Tuberc Lung Dis.

[54] Shimizu Y, Dobashi K, Mita Y (2006). DNA microarray genotyping of N-acetyltransferase 2 polymorphism using carbodiimide as the linker for assessment of isoniazid hepatotoxicity. Tuberculosis.

[55] Sotsuka T, Sasaki Y, Hirai S, Yamagishi F, Ueno K (2011). Association of isoniazid-metabolizing enzyme genotypes and isoniazid-induced hepatotoxicity in tuberculosis patients. In Vivo.

[56] Teixeira R L dF, Morato R G, Cabello P H (2011). Genetic polymorphisms of NAT2, CYP2E1 and GST enzymes and the occurrence of antituberculosis drug-induced hepatitis in Brazilian TB patients. Mem Inst Oswaldo Cruz.

[57] Vuilleumier N, Rossier M F, Chiappe A (2006). CYP2E1 genotype and isoniazid-induced hepatotoxicity in patients treated for latent tuberculosis. Eur J Clin Pharmacol.

[58] Wang J Y, Tsai C H, Lee Y L (2015). Gender-dimorphic impact of PXR genotype and haplotype on hepatotoxicity during antituberculosis treatment. Medicine.

[59] Wang J-Y, Liu C-H, Hu F-C (2011). Risk factors of hepatitis during anti-tuberculous treatment and implications of hepatitis virus load. J Infect.

[60] Xiang Y, Ma L, Wu W (2014). The incidence of liver injury in Uyghur patients treated for TB in Xinjiang Uyghur autonomous region, China, and its association with hepatic enzyme polymorphisms nat2, cyp2e1, gstm1 and gstt1. PLOS ONE.

[61] Yamada S, Tang M, Richardson K (2009). Genetic variations of NAT2 and CYP2E1 and isoniazid hepatotoxicity in a diverse population. Pharmacogenomics.

[62] Yuliwulandari R, Susilowati R W, Wicaksono B D (2016). NAT2 variants are associated with drug-induced liver injury caused by anti-tuberculosis drugs in Indonesian patients with tuberculosis. J Hum Genet.

[63] Zaverucha-do-Valle C, Monteiro S P, El-Jaick K B (2014). The role of cigarette smoking and liver enzymes polymorphisms in anti-tuberculosis drug-induced hepatotoxicity in Brazilian patients. Tuberculosis.

[64] Yimer G, Ueda N, Habtewold A (2011). Pharmacogenetic & pharmacokinetic biomarker for efavirenz based ARV and rifampicin based anti-TB drug induced liver injury in TB-HIV infected patients. PLOS ONE.

[65] Brito T C, Possuelo L G, Valim A R (2014). Polymorphisms in CYP2E1, GSTM1 and GSTT1 and anti-tuberculosis drug-induced hepatotoxicity. An Acad Bras Cienc.

[66] Fernandes D C R O, Santos N P C, Moraes M R (2015). Association of the CYP2B6 gene with anti-tuberculosis drug-induced hepatotoxicity in a Brazilian Amazon population. Int J Infect Dis.

[67] Huang Y S, Chern H D, Su W J (2002). Polymorphism of the *N*acetyltransferase 2 gene as a susceptibility risk factor for antituberculosis drug–induced hepatitis. Hepatology.

[68] Rana S, Ola R, Sharma S K (2012). Comparison between acetylator phenotype and genotype polymorphism of n-acetyltransferase-2 in tuberculosis patients. Hepatol Int.

[69] Singh M, Gupta V H, Amarapurkar D N (2014). Association of genetic variants with anti-tuberculosis drug induced hepatotoxicity: a high resolution melting analysis. Infect Genet Evol.

[70] Roy B, Chowdhury A, Kundu S (2001). Increased risk of antituberculosis drug-induced hepatotoxicity in individuals with glutathione S-transferase M1 ‘null’ mutation. J Gastroenterol Hepatol.

[71] Jorgensen A L, FitzGerald R J, Oyee J, Pirmohamed M, Williamson P R (2012). Influence of CYP2C9 and VKORC1 on patient response to warfarin: a systematic review and meta-analysis. PLOS ONE.

[72] Contopoulos-Ioannidis D G, Alexiou G A, Gouvias T C, Ioannidis J P (2006). An empirical evaluation of multifarious outcomes in pharmacogenetics: beta-2 adrenoceptor gene polymorphisms in asthma treatment. Pharmacogenet Genomics.

[73] Sabbagh A, Langaney A, Darlu P, Gérard N, Krishnamoorthy R, Poloni E S (2008). Worldwide distribution of NAT2 diversity: implications for NAT2 evolutionary history. BMC Genet.

[74] Little J, Higgins J P, Ioannidis J P (2009). STrengthening the REporting of Genetic Association Studies (STREGA): an extension of the STROBE statement. Hum Genet.

